# Synthesis and evaluation of designed PKC modulators for enhanced cancer immunotherapy

**DOI:** 10.1038/s41467-020-15742-7

**Published:** 2020-04-20

**Authors:** Clayton Hardman, Stephen Ho, Akira Shimizu, Quang Luu-Nguyen, Jack L. Sloane, Mohamed S. A. Soliman, Matthew D. Marsden, Jerome A. Zack, Paul A. Wender

**Affiliations:** 1 Departments of Chemistry and of Chemical and Systems Biology, Stanford University, Stanford, CA 94305 USA; 2 Department of Microbiology, Immunology, and Molecular Genetics, University of California, Los Angeles, Los Angeles, CA 90095 USA; 3 Division of Hematology and Oncology, Department of Medicine, University of California, Los Angeles, Los Angeles, CA 90095 USA

**Keywords:** Cancer immunotherapy, Diversity-oriented synthesis, Chemical libraries, Synthetic chemistry methodology

## Abstract

Bryostatin 1 is a marine natural product under investigation for HIV/AIDS eradication, the treatment of neurological disorders, and enhanced CAR T/NK cell immunotherapy. Despite its promising activity, bryostatin 1 is neither evolved nor optimized for the treatment of human disease. Here we report the design, synthesis, and biological evaluation of several close-in analogs of bryostatin 1. Using a function-oriented synthesis approach, we synthesize a series of bryostatin analogs designed to maintain affinity for bryostatin’s target protein kinase C (PKC) while enabling exploration of their divergent biological functions. Our late-stage diversification strategy provides efficient access to a library of bryostatin analogs, which per our design retain affinity for PKC but exhibit variable PKC translocation kinetics. We further demonstrate that select analogs potently increase cell surface expression of CD22, a promising CAR T cell target for the treatment of leukemias, highlighting the clinical potential of bryostatin analogs for enhancing targeted immunotherapies.

## Abstract (web summary)

Bryostatin 1 is a unique therapeutic lead, however its scarce natural sources have hampered its use in treatment of human disease. Here, the authors use a scalable synthesis of bryostatin 1 to make close-in analogs which potently induce increased cell surface expression holding potential for immunotherapy.

## Introduction

Targeted biologics and cell therapies for the treatment of cancer, including monoclonal antibodies (mAbs)^1^, antibody-drug conjugates (ADC’s)^2^, bi- and tri-specific antibodies (biAbs, triAbs)^3^, chimeric antigen receptor (CAR) T and NK cell therapies, and neoantigen-directed cell therapies^4–6^, are revolutionizing oncology. By targeting tumor-specific cell surface antigens and neoantigens, these therapies offer distinct advantages over traditional treatment options as they avoid the systemic toxicity associated with cytotoxic chemotherapies while efficiently and selectively clearing malignant cells^7,8^. Although these cell therapies rely on vastly different mechanisms of action and leverage different host biological systems for tumor clearance, each is fundamentally based on a common requirement, specifically sufficient and sustained target (neo)antigen cell surface expression. Notwithstanding the clinical promise of these recent advances in immuno-oncology, tumor escape and acquired resistance driven by decreased surface expression of target (neo)antigens limit the efficacy, durability and scope of these approaches. Indeed, poor durability of response and patient relapse associated with variable and decreased antigen density have been observed across several indications^4,5,9–12^. The identification of adjuvants that enhance and sustain surface expression of target (neo)antigens could broadly address this problem and improve the efficacy of these approaches by turning cold tumors into hot ones^13^, which could then be more effectively cleared with targeted biologics and cell therapies thereby potentially increasing not only the number of patient responders but the durability of their response.

Protein kinase C (PKC) modulators offer a potentially general solution to this antigen density and persistence problem. Among the most studied PKC modulators, plant-derived phorbol esters (PEs) have long been known to influence antigen expression in a variety of cell lines^14–18^. Prompted by such findings and with the intent to identify simplified PE analogs using a function-oriented synthesis (FOS) strategy^19–21^, we reported in 1986 a computer-based analysis of the pharmacophore of PEs and related PKC modulating ligands, which led to the first designed PKC modulator—3-(*N*-acetylamino)-5-(*N*-decyl-*N*-methylamino)-benzyl alcohol (ADMB)^22^. In 1992, Leon et al.^23^ showed that ADMB, like the PEs, enhanced tumor associated antigen (BCA-225) expression in breast carcinoma cells but, unlike PEs, it did not induce shedding of the antigen, highlighting the potential for natural product-inspired designed analogs to exhibit divergent and superior biological activity. Significantly, the immunomodulatory properties of the PEs are not unique to this structural class of PKC modulators. For example, bryostatin 1, a marine macrolide and potent PKC modulator^24,25^, also alters expression of surface antigens in tumor cell lines, making them more immunogenic and thus more susceptible to immune or other antigen-targeted clearance strategies^17,26–30^. Indeed, several pre-clinical and clinical studies have reported that bryostatin 1 can alter the immunophenotype and increase the immunogenicity of cancer cells in acute lymphoblastic leukemia (ALL), chronic lymphocytic leukemia (CLL), and non-Hodgkin’s lymphoma (NHL)^17,26–31^. These and related studies by a Spaner–Wender collaboration on CLL antigen expression and by a Zack–Marsden–Wender collaboration on CD69 expression in CD4+ T cells, the latter related to HIV eradication, indicate that PKC modulators can enhance expression and persistence of certain antigens, potentially enhancing a variety of (neo)antigen-targeted therapies^17,30,32–34^.

Among antigen-targeting strategies in clinical evaluation, CAR T cell therapy has recently emerged as an effective treatment for B-cell malignancies. Specifically, CAR T cells targeting the B cell-restricted antigen CD19 (Tisagenlecleucel (Kymriah, Novartis), Axicabtagene ciloleucel (Yescarta, Gilead)) have produced significant clinical responses in the treatment of leukemias and lymphomas^35^. However, antigen loss has been observed as a primary driver in acquired resistance and patient relapse^4,5,11,35,36^. To address this mechanism of tumor escape, researchers have expanded their target portfolio to other B cell-restricted antigens, culminating in the recent development of an anti-CD22 CAR T cell therapy^11^. Preliminary data from a phase I trial for ALL with patients who were naïve or resistant to CD19-targeted CAR immunotherapy was promising, with ~70% of patients achieving complete remission for a median duration of 6 months^11^. However, resistance driven by diminished and variable levels of CD22 surface expression was observed in a subset of patients who were unresponsive or relapsed following treatment. Importantly, bryostatin 1 has been shown to upregulate CD22 surface expression in ALL, CLL and NHL cells both in vitro and ex vivo^26,28,31,37^, and in vivo in human clinical trials^27,29^. This suggests that bryostatin 1 and by analogy its analogs could be used in combination with anti-CD22 CAR T therapy to improve patient outcomes by ensuring that malignant cells maintain sufficient levels of CD22 surface expression to be effectively cleared. Indeed, Ramakrishna et al.^31^ recently demonstrated that natural bryostatin 1 can improve CD22-targeted CAR T activity in a pre-clinical model of ALL.

Given the potential of PKC modulators to enhance targeted therapies, it is especially noteworthy that the vast majority of research in this area over the past several decades has focused on only a small number of natural products and more specifically bryostatin 1. This is especially significant because these agents, while promising clinical leads, are neither evolved nor optimized for clinical use. As evident from studies on the taxanes and avermectins, immediate synthetic precursors and derivatives of natural products, so called close-in analogs, often provide superior clinical performance^38,39^. Indeed, while many drugs are inspired by natural products, few (ca. 6%), as evident from new chemical entities reported between 1981 and 2014, are natural products themselves^40^. This has increasingly motivated the bio-inspired and synthesis-informed design of close-in or simplified structures with distinct or superior function^19–21^.

Unfortunately, synthetic access to close-in analogs of bryostatin 1 has been precluded by the limited availability of natural and synthetic material, which is further exacerbated by the inherent challenges associated with modifying the exquisitely complex bryostatin scaffold. The original hand collection of natural bryostatin 1, which provided GMP material for clinical use, produced only 18 g of material from 14 tons of the source organism *Bugula neritina* (0.00014% yield) and that is now nearly depleted^41^. Re-collection from this marine source would raise cost, sustainability and environmental concerns due to bryostatin’s poor and variable natural availability^42^. Aquaculture and engineered biosynthesis have been explored, but the former encountered capitalization and yield problems and the latter difficulties in cultivation of the symbiotic bacterium necessary for production of bryostatin 1^43^. Recently, we reported a solution to this supply problem, a scalable synthesis of bryostatin 1, that has afforded sustainable access to gram scale quantities of the natural product as needed to ensure further research and its continued clinical evaluation^44^. Additionally as reported herein, our chemical synthesis also serves as a platform for accessing bryostatin analogs and the exploration of structure–function relationships, thereby enabling the design and synthesis of potentially more accessible, more efficacious and better tolerated bryostatin-inspired leads. Given that bryostatin 1 is in pre-clinical and clinical studies for widely varied indications including the treatment of Alzheimer’s disease^45–47^, eradication of HIV/AIDS^48–50^, multiple sclerosis^51^, Niemann Pick disease^52^, Fragile X^53^ and enhanced immunotherapy^17,25,30,31^, and given that many of these indications involve different PKC isoforms, access to varied analogs avoids a one-size-fits-all single agent approach to varied therapeutic indications and offers a more disease specific optimization opportunity.

Here we report the design, synthesis and evaluation of the first close-in bryostatin 1 analogs. Our design strategy focuses on making chemical modifications to the bryostatin scaffold that would be expected to retain compound affinity to PKC, but could influence PKC function and downstream signaling outcomes while also potentially being used as needed to tune formulation and ADME characteristics. 18 analogs were prepared and their activities were compared with synthetic bryostatin 1. Based on an FOS strategy, these compounds were designed to retain the pharmacophoric functionalities proposed for PKC binding in our original pharmacophore model^22,54^. Consistent with that model, most of these close-in analogs exhibited single-digit nanomolar affinities to representative PKC isoforms, comparable to bryostatin 1^22,54^. In contrast, we observe a diverse array of activity profiles in a functional assay measuring PKC translocation in real time in living cells, suggesting that such modifications can indeed elicit differential biological functions, irrespective of cell-free binding affinities. Significantly, we also investigate the ability of these analogs to increase CD22 surface expression in in vitro models of ALL and AIDS-related lymphomas in connection with emerging antigen-targeted therapies. We found that several analogs exhibit activity similar to bryostatin 1, suggesting that these compounds could serve as more accessible and efficacious, and potentially better tolerated, adjuvants for cancer immunotherapy and other therapeutic indications.

## Results

### Design and synthesis of bryostatin analogs

**Fig. 1:**
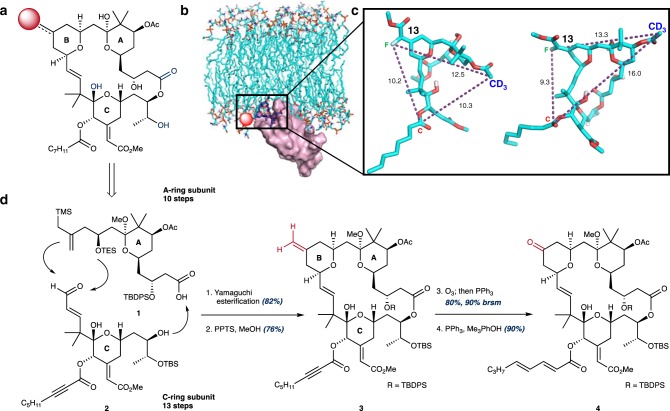
FOS design strategy for the synthesis of bryostatin analogs. **a** Retrosynthetic analysis of the bryostatin scaffold with key pharmacophoric elements highlighted in blue (C1 carbonyl, C19 hemiketal, C26 alcohol). C13 (red sphere) highlighted as a diversification node for analog synthesis. **b** Rendering of the PKC-bryostatin-membrane ternary complex. Bryostatin 1 shown in dark blue. Pharmacophoric elements of the C-ring subunit of the bryostatin scaffold (**a**) interact directly with PKC (purple) while the A and B rings are imbedded in the plasma membrane (cyan). Adapted with permission from Yang et al. (https://pubs.acs.org/doi/full/10.1021/acscentsci.7b00475) and the American Chemical Society (ACS)^56^. Further permissions related to the material excerpted should be directed to the ACS. **c** Representative conformers of the bryostatin scaffold bound to PKC that fit experimentally determined intramolecular distances determined by REDOR NMR. Adapted with permission from Yang et al. (https://pubs.acs.org/doi/full/10.1021/acscentsci.7b00475) and the ACS^56^. Further permissions related to the material excerpted should be directed to the ACS. **d** Convergent construction of the bryostatin scaffold from acid **1** and enal **2**. C13 functionality (highlighted in red) provides a versatile functionalization node for late-stage diversification, avoiding interference with the pharmacophoric elements of the C-ring (highlighted in blue, **a**).

Our FOS design strategy for bryostatin analogs was guided by our previously proposed bryostatin pharmacophore model^54^ and further supported by recent molecular dynamics simulations^55^ and REDOR NMR structural studies^56^. PKC is a family of seven homologous signaling kinases classified as conventional (*α*, *β*, *γ*) or novel (*δ*, *ε*, *η*, *θ*) based on their subdomain architectures^57^. Individual isoforms, combinations of isoforms, and mutant isoforms of PKC are implicated in a number of disease pathologies^58,59^. PKC maturation and activation are governed by a sequence of highly orchestrated phosphorylations, conformational changes, and ultimately translocation to the plasma membrane where they effect phosphorylations of numerous downstream signaling proteins^57,60^. The hallmark of ligand-induced PKC activation is formation of a ternary complex between the plasma membrane, ligand and PKC (Fig. 1b)^57,58,60^. In this context, the bryostatin scaffold can be considered as two subunits that have distinct but interdependent functions. Our computational studies revealed that hydrogen-bonding functionalities around the C-ring subunit of the bryostatin scaffold, specifically the C1 carbonyl, C26 hydroxyl and C19 hemiketal, are spatially preorganized by the A and B rings into a binding conformation that mimics PKC’s endogenous ligand, DAG (Fig. 1a)^54–56,61^. Indeed, seminal studies by our group showed that modification or deletion of any of the C1, C26 and C19 functionalities reduced or eliminated PKC binding while changes in the A- and B-rings generally had little or no effect on binding, suggesting that changes to these subunits could be used to change physical properties, biological function and biodistribution and thereby potentially improve efficacy and tolerability^20,54,61–64^. We therefore set out to determine which changes in close-in analogs would justify future progression of the better performers to more advanced in vivo studies.

In addition to spatially restricting the pharmacophoric elements of bryostatin’s C-ring subunit to a productive PKC-binding conformation, the northern subunit is also thought to influence translocation efficiency of PKC as well as the depth and orientation of the ligand-PKC complex in membranes, as suggested by recent long-timescale (400–500 μs) molecular dynamics simulations^55^. Specifically, interaction of oxygenation in the A and B-rings with water molecules at the membrane–cytosol interface putatively causes the bryostatin-PKC complex to adopt a shallow, angled orientation with respect to the membrane (Fig. 1b)^55^. This unique orientation of the bryostatin 1-PKC complex was not populated when other potent PKC ligands such as phorbol dibutyrate (PDBu) and prostratin were modeled, providing an explanation for their competitive PKC binding but often contrasting downstream activities^55^. In a separate study, a combination of REDOR NMR and molecular dynamics simulations identified a distribution of PKC-bound bryostatin conformers (Fig. 1c), a feature that was unique to the bryostatin scaffold and not observed in a phorbol ester derivative^56^. Together, these studies suggest that the multiple PKC-bound conformers available to the bryostatin scaffold could generate differential orientations of the activated PKC-ligand complex and influence PKC’s interactions with downstream effector proteins, thereby explaining bryostatin’s unique biological activity. This in turn suggests that modifications to the A and B rings of the bryostatin scaffold could influence compound function by affecting the orientation of the active PKC signaling complex. With this decades-derived guiding framework in mind, we designed a series of compounds that retain the pharmacophoric elements of the bryostatin C-ring but incorporate modifications to the bryostatin B-ring, focusing on C13 as a diversification node. This site was selected because our computational studies suggest that modifications at this non-pharmacophoric position would preserve PKC-binding affinity while enabling exploration of its influence on compound function (e.g. translocation, activity and tolerability). By leveraging the unique reactivity at this position found in late-stage synthetic intermediates we were able to generate a set of close-in bryostatin analogs with diverse chemical functionality at this position to explore how various substitution patterns in the B-ring fragment can affect compound function by influencing membrane association, trafficking and downstream signaling outcomes.

Recently we reported a scalable, step-economical synthesis of bryostatin 1, which allows for the convergent assembly of a diversifiable bryostatin macrocyclic skeleton (exo-olefin **3**, Fig. 1d) from two synthetically accessible building blocks (Fig. 1d)^44^. This study provided a synthetic supply (>2 g) of bryostatin 1 that is required to support synthesis studies. The bryostatin fragments, carboxylic acid **1** and enal **2**, are brought together in a Yamaguchi esterification that proceeds in 82% yield. Formation of bryostatin’s B-ring and closure of the macrocycle is then accomplished by employing our previously reported Prins-driven macrocyclization strategy (Fig. 1d), which generates exo-olefin **3**. Drawing on the richness of alkene and ketone chemistry, exo-olefin **3** can be selectively modified to directly install new B-ring functionality or converted into the corresponding ketone via a pi-selective stoichiometric ozonolysis procedure developed by our group (Fig. 1d). These two functional groups afford chemical reactivity that is largely orthogonal to the remaining functionality and provide starting points for exploring how modifications to bryostatin’s B-ring can influence PKC function (affinity, translocation, and signaling). Owing to the extremely high cost of natural bryostatin (ca. $1500/mg), the limited current amount of synthetic bryostatin and its precursors until a production scale-up synthesis is completed, and the intrinsic challenges of modifying such a complex scaffold with limited materials, we intentionally chose to use chemistries that would carry less risk and thus loss of precious materials. Furthermore, as we needed to work on multi-milligram scales (~5–10 mg) to enable compound characterization and biological evaluation, we limited our initial focus to the B-ring as our modeling and prior studies suggested that region tolerated change.

**Fig. 2:**
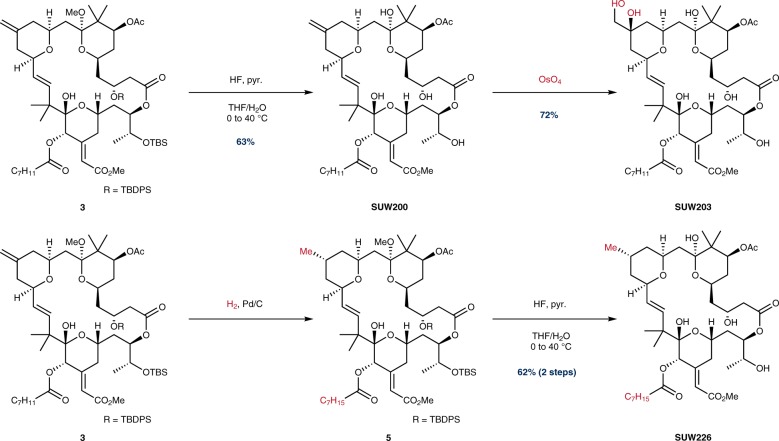
Chemoselective functionalization of the C13 exo-olefin. Detailed procedures for specific transformations can be found in the Supplementary Methods. C_7_H_11_ = 2-heptynyl.

In our initial exploration of chemical space at C13, we sought to leverage the differentiated reactivity of the C13 exo-olefin over other pi-systems to generate a series of close-in structural analogs. For steric and electronic reasons, the C13 olefin was expected to be the most reactive of the four pi-systems found in this advanced intermediate (Fig. 2, olefin **3**)^44^. As previously described, global deprotection of exo-olefin **3** afforded **SUW200**^44^. Gratifyingly, di-hydroxylation of **SUW200** selectively afforded vicinal diol **SUW203** in the presence of the C16/C17 alkene, C20 alkynoate and C21 enoate. In contrast, and in line with our concerns about the selectivity of even routine reactions on such a complex scaffold, epoxidations of the exo-olefin **3** and bryostatin 1 with DMDO or mCPBA proved challenging. Furthermore, attempts to reduce the C13 olefin using H_2_ and catalytic Pd/C were also difficult to control, often providing mixtures of reduction products. However, we were able to eventually achieve clean reduction of the C13 exo-olefin and C20 alkynoate without reducing either the C21 enoate or the C16/17 olefin by decreasing reaction time and concentration, generating compound **5**, which was subsequently deprotected to afford **SUW226**.

**Fig. 3:**
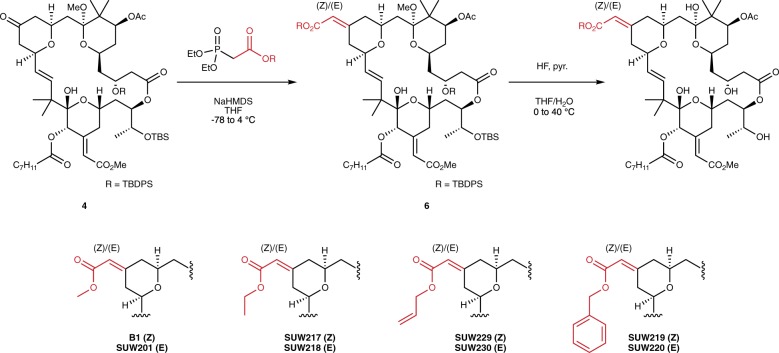
Synthesis of C13 enoates via HWE olefination of the C13 ketone. Detailed procedures for specific transformations can be found in the Supplementary Methods.

We next focused on modification of the C13 ketone **4**. This advanced intermediate served as an ideal diversification node as the C13 ketone presents chemical reactivity that is orthogonal to the rest of the functionality found in this series of advanced intermediates (Fig. 3). To conserve material, we began with the synthesis of a limited series of C13 alkyl enoates. From ketone **4**, we generated compounds with various ester groups to explore how substituent size and enoate geometry influence compound function. Ester derivatives were accessed via a Horner–Wadsworth–Emmons (HWE) olefination of the densely functionalized ketone **4** using HWE reagents that were prepared via straightforward coupling of the desired alcohol to diethyl phosphonoacetic acid. Contrasting the use of a chiral HWE reagent, which controls double bond geometry as reported in our synthesis of bryostatin 1, we chose to perform these reactions using simple, non-stereoselective HWE reagents to access both the (*E*) and (*Z*) isomers of enoate **6**. Subsequent global deprotection and HPLC separation of the enoate diastereomers afforded the indicated analogs (Fig. 3).

**Fig. 4:**
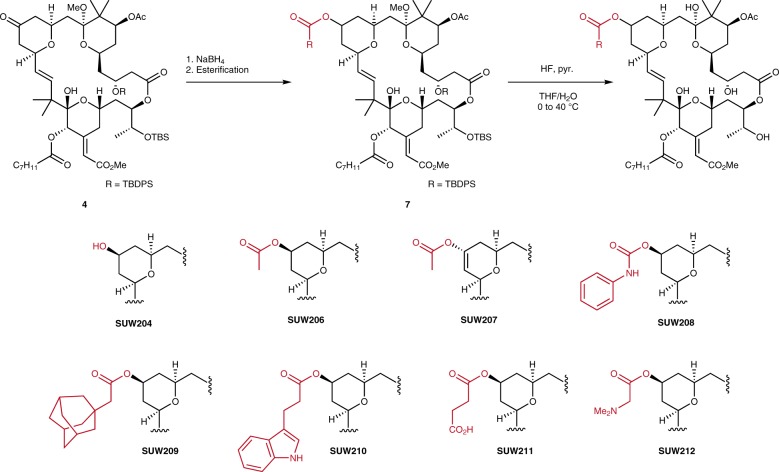
Synthesis of C13 esters via reduction and esterification of C13 ketone. Esters were formed using standard esterification chemistries, including acylation with anhydrides, carbodiimide coupling, and carbamoylation with phenyl isocyanate (**SUW208**). Detailed procedures for specific transformations can be found in the Supplementary Methods.

In addition, installation of an alcohol at C13 was expected to provide a convenient functional handle for the late-stage diversification through esterification and an opportunity to explore how C13 hybridization affects function. This was readily accomplished via borohydride reduction of ketone **4** and esterification of the resultant alcohol (Fig. 4), giving rise to a structurally diverse set of compounds, which included the C13 alcohol, aliphatic esters, an indolyl ester, a phenyl carbamate, and both cationic and anionic moieties.

### Biological evaluation of C13-modified bryostatin analogs

**Fig. 5:**
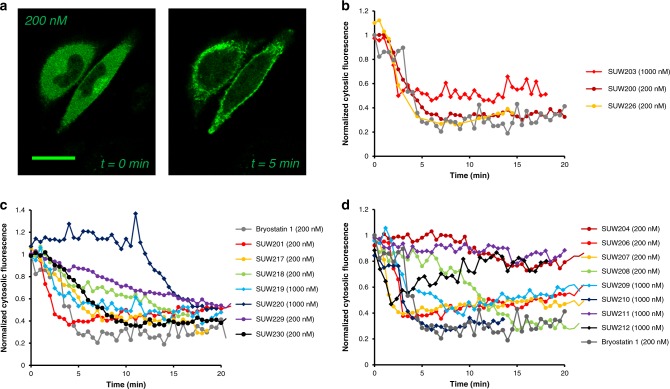
Bryostatin-induced PKC translocation. **a** Bryostatin-induced translocation of PKC from the cytosol to the membrane was determined in real time by monitoring cytosolic fluorescence of a PKCδ-GFP fusion protein using confocal microscopy. Scale bar represents 25 μm. Experiments performed in *n* = 3 biological replicates. **b**–**d** Cytosolic fluorescence normalized to *t* = 0 (time immediately prior to addition of compound to media) and plotted against time. Error bars excluded for clarity (data presented as the mean of *n* = 3 biological replicates). Individual curves with error bars included in Supplementary Information. Maximum translocation of PKCδ-GFP to the plasma membrane reported in Table 1.

With a panel of compounds bearing diverse functionality at C13, we began to explore how these modifications affect PKC binding and biological function. Because an obvious prerequisite for PKC pathway involvement is binding to PKC, compounds were initially evaluated for PKC affinity in a cell-free competitive binding assay with tritiated phorbol dibutyrate ([^3^H]-PDBu). Assays were performed with representative members of both the conventional (PKCα) and novel (PKCδ) PKC families. Subsequently, compounds were assayed for PKC isoform translocation in real time in living cells using an optical assay. Translocation to the plasma membrane is the hallmark of PKC activation^18,57,60^ and therefore monitoring the subcellular localization of a PKCδ-GFP fusion protein via confocal microscopy offers a convenient method for determining whether a compound enters a cell and engages its PKC isoform target^64^. Finally and of clinical significance, we also determined the effect of compounds on CD22 surface expression in vitro in NALM6 cells, an ALL cell line previously studied in connection with CD22-targeted CAR T cell therapy, and two additional lymphoma cell lines^11,31,65^. PKC binding, translocation and CD22 surface expression data are summarized in Fig. 5 and Table 1.

**Table 1:** Biological evaluation of bryostatin analogs.

Compound	*K*_i_ PKCα (nM)	*K*_i_ PKCδ (nM)	% Translocation (concentration^a^)	Fold increase CD22 expression^b^
**Bryostatin 1**	0.8	1.1	70% (200 nM)	2.10
**SUW200**	4.1	7.6	70% (200 nM)	1.73
**SUW201**	0.54	1.4	65% (200 nM)	1.59
**SUW203**	1.0	4.1	50% (1000 nM)	1.16
**SUW204**	1.4	3.7	25% (200 nM)	0.98
**SUW206**	1.0	1.3	60% (200 nM)	1.63
**SUW207**	2.0	5.4	60% (200 nM)	1.33
**SUW208**	1.0	4.7	75% (200 nM)	1.68
**SUW209**	1.6	3.5	60% (1000 nM)	1.45
**SUW210**	5.6	7.3	75% (1000 nM)	1.49
**SUW211**	98	27	20% (1000 nM)	ND
**SUW212**	40	24	50% (1000 nM)^c^	ND
**SUW217**	9.2	9.4	75% (200 nM)	1.97
**SUW218**	7.1	6.6	55% (200 nM)	1.92
**SUW219**	37	25	60% (1000 nM)	ND
**SUW220**	16	15	50% (1000 nM)	ND
**SUW226**	5.8	6.6	75% (200 nM)	1.76
**SUW229**	1.8	1.2	50% (200 nM)	2.35
**SUW230**	4.2	9.4	65% (200 nM)	1.62

Compounds were evaluated for PKC-binding affinity in a competitive binding assay with [^3^H]-phorbol dibutyrate in PKCα and PKCδ, members of the conventional and novel PKC isoform families, respectively. Cell entry and PKC activation in living cells was determined by monitoring translocation of a PKCδ-GFP fusion protein from the cytosol to the plasma membrane. Representative images included in Fig. 5.

*ND* not determined.

^a^Indicates minimum effective concentration required to induce translocation of PKC-GFP to the membrane.

^b^In NALM6 cells at 1 nM relative to DMSO control (*n* = 3 biological replicates).

^c^Indicates only brief translocation observed (Fig. 5d).

Our goal was to determine whether PKC affinity and in vitro PKC activation (measured via intracellular PKC translocation) correlate, and how binding and translocation influence downstream function (CD22 expression). Consistent with our computer modeling studies, nearly all of the compounds designed using our pharmacophore model retained potent PKC-binding affinity (<10 nM), comparable to bryostatin 1 (Table 1). However, certain C13 functionalities decreased PKC affinity due to putative differences in membrane partitioning or ligand steric effects. For example, charged substituents at C13 (**SUW211**, **SUW212**) decreased affinity to PKC by ~20-100-fold (Table 1), consistent with the less effective partitioning of these groups in the phosphatidyl serine (PS) vesicle complex (membrane surrogate used in the cell-free binding assay). In addition, substitution of the C13 methyl-(*Z*)-enoate with a benzyl-(*Z*)-enoate resulted in a ~30-fold decrease in affinity, suggesting a size limit to groups at this site. Similar decreases in binding affinity were not observed in C13 esters with more conformationally flexible linkers bearing large, relatively hydrophobic substituents (**SUW209**, **SUW210**, Table 1). Aside from these exceptions, the majority of compounds tested exhibited single-digit nanomolar binding affinities to PKC, further supporting the predictive value of our proposed pharmacophore model and prompting the progression of these compounds to more advanced in vitro PKC translocation assays.

Cell-free ligand binding to PKC is a necessary but not sufficient prerequisite to elicit function, as a ligand must enter the cell and engage PKC to form a functional PKC-ligand membrane complex. Both experimental studies and molecular dynamics simulations suggest that the PKC-ligand complex can assume multiple bound states, putatively influencing the differential association of scaffolding proteins and phosphorylation of downstream effector proteins, thereby resulting in diverse biological outcomes^18,55–57,60^. Therefore, our next goal was to determine whether the most potent PKC binders enter cells and show similar or different dynamic interactions with PKC.

Significantly, our studies show that the thermodynamics of PKC binding and the kinetics of ligand cell entry and PKC translocation are often decoupled, a finding that could be exploited to control isoform-selective signaling. As our positive comparator, synthetic bryostatin 1, a single-digit nanomolar binder of all PKC isoforms, translocates ~70% of cytosolic PKCδ-GFP to the plasma membrane within 5 min at a concentration of 200 nM (Table 1, Fig. 5). While introducing hydrophilic substituents at C13 (**SUW203**, **SUW204**) generated compounds that bind both conventional and novel PKC isoforms with single-digit nanomolar affinity, these analogs exhibited different behavior than bryostatin 1 in the translocation assay (Fig. 5b, c). At 200 nM **SUW204** translocated only ~25% of cytosolic PKC to the plasma membrane at 20 min while **SUW203** required a concentration of 1000 nM to translocate ~50% of cytosolic PKC. Similarly, compounds with charged substituents at C13 (**SUW211**, **SUW212**) did not achieve sustained translocation of PKC at up to 1000 nM (Table 1, Fig. 5d). This suggests that interaction of the membrane with the functionality on bryostatin’s B-ring is critical for compound function and that PKC binding is necessary, but not sufficient for compound activity in vitro. Compounds with hydrophilic or charged modifications at C13 would putatively inefficiently cross or embed in the plasma membrane, and thus would exhibit attenuated activity. This changes how one thinks about PKC modulators from an emphasis on the thermodynamics of binding to the additional issue of translocation kinetics.

We also examined a series of C13 esters of different sizes, hydrophobicities, and varied structural motifs (aryl, heteroaryl, alkyl, adamantyl), all of which could be efficiently accessed from the C13 alcohol (Fig. 4). Although we found that diverse functionality was tolerated at this position with respect to PKC-binding affinity, our analogs exhibited vastly different translocation dynamics. In contrast to the free alcohol **SUW204**, capping the C13 hydroxyl group with smaller hydrophobic substituents generated compounds that were comparable to bryostatin 1 at translocating PKC (Fig. 5d). The two diastereomers of the C13 acetate (**SUW206** and **SUW207**) exhibited almost identical behavior both to each other and to bryostatin 1. Although C13 phenyl carbamate **SUW208** effectively translocated PKC to the plasma membrane at 200 nM, it exhibited a delayed time course, requiring ~15 min for translocation, relative to other analogs that were active in this assay (Fig. 5d), indicating that small changes in size and/or polarity at this position can influence ligand-mediated PKC activation and signaling and/or the kinetics and efficacy of cell entry.

To complement the synthesis of compounds with relatively similar sized substituents at C13, we also installed larger substituents with vastly different functionalities than are found in the natural product. C13 adamantyl ester **SUW209** and C13 indoyl ester **SUW210** were designed to enhance potential membrane interactions via increased localized hydrophobicity or to pick up cation-pi contacts with cationic lipid headgroups on the membrane, respectively. We found that while both compounds are high affinity PKC binders (Table 1), they are inactive at 200 nM in the PKC translocation assay and require 1000 nM to translocate ~70% of cytosolic PKC to the plasma membrane at ~5 min (Fig. 5d).

This finding, made possible by this collection of close-in analogs, prompted us to examine how C13 functionality can influence bulk compound properties and the correlation between compound hydrophobicity (measured using cLogP) and activity (PKC translocation). Plotting cLogP vs. percentage of membrane associated PKCδ-GFP at 200 nM (Supplementary Figure 56) revealed an effective window of cLogP values required for efficient translocation. Compounds with cLogP values between 1.0 and 4.0 were active at 200 nM, suggesting that lipophilicity must be effectively balanced to retain activity. It is also noteworthy that among compounds within the effective range of lipophilicity, variable dynamics of PKC activation are observed. Although most compounds exhibit logarithmic activation curves, select compounds exhibit delayed activation patterns (**SUW208**, Fig. 5d), or more sustained linear activation dynamics (**SUW218**, **SUW229**, Fig. 5c). These observations of the kinetics of PKC-ligand association and intracellular distribution of the complex can be rationalized by the timing of ligand entry into the cell and post-entry ligand-controlled partitioning of the PKC-ligand complex between the plasma membrane and cytosol, with more polar compounds having slower entry and a limited ability to form a stable complex with the plasma membrane. This is of potential clinical consequence as it is well known that the dynamics of PKC activation can have profound consequences for downstream signaling outcomes^47,58^.

C13 alkyl enoates in both the (*Z*) and (*E*) geometries were well tolerated with the exception of benzyl enoates **SUW219** and **SUW220**. Interestingly, benzyl enoate **SUW219** binds PKC with a ~2-fold decrease in affinity, suggesting that perhaps the (*Z*) olefin geometry can position C13 substituents in an orientation that impacts the conformation of pharmacophoric elements in the C-ring. In general however, smaller, linear alkyl enoates are potent binders of PKC and active ligands in vitro (Table 1, Fig. 5c), although they do affect PKC translocation dynamics.

**Fig. 6:**
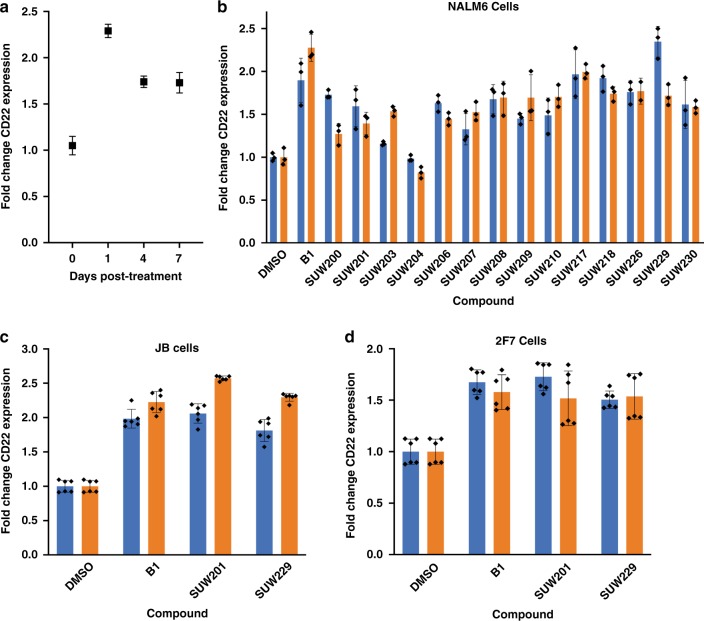
Bryostatin-promoted cell surface expression of CD22. **a** Synthetic bryostatin 1 promotes increased surface expression of CD22. NALM6 cells were incubated with 10 nM bryostatin 1 for 24 h. Compound was washed out and cells were sub-cultured for the indicated times. CD22 surface expression was then assayed by flow cytometry (*n* = 3 biological replicates; data presented as mean values ± SE). **b** NALM6 cells were incubated with 1 nM (blue bars) or 10 nM (orange bars) compound for 24 h. Compound was washed out and cells were sub-cultured for 24 h. CD22 surface expression was then assayed by flow cytometry (*n* = 3 biological replicates; data presented as mean values ± SE). **c** JB cells were incubated with 1 nM (blue bars) or 10 nM (orange bars) of compound for 48 h. CD22 surface expression was then assayed by flow cytometry (*n* = 6 biological replicates; data presented as mean values ± SE). **d** 2F7 cells were incubated with 1 nM (blue bars) or 10 nM (orange bars) of compound for 48 h. CD22 surface expression was then assayed by flow cytometry (*n* = 6 biological replicates; data presented as mean values ± SE).

Having established the potent affinities of members of our analog library and their PKC translocation kinetics, we next sought to evaluate our close-in analogs in an assay pertinent to the clinical use of bryostatin and its analogs as adjuvants to enhance targeted cancer immunotherapies. Our approach focused on CD22-targeted CAR T cell therapy, which has been reported to cure patients with ALL while those who fail this treatment are thought to have a lower surface density of CD22^11,31,65^. Fry et al.^65^ demonstrated that a critical threshold of CD22 surface density is required for activation of anti-CD22 CAR T cells in vitro and tumor clearance in a murine tumor xenograft model^11^. More recently, Ramakrishna et al.^31^ demonstrated that natural bryostatin 1-promoted increases in CD22 surface expression can improve the efficacy and durability of CAR T-mediated tumor clearance in in vivo models of ALL, suggesting that bryostatin and as yet unexplored analogs could be used to improve patient outcomes when used in combination with anti-CD22 CAR T therapy. To investigate synthetic bryostatin 1 and bryostatin analogs as adjuvant leads for CD22-targeted CAR T therapy, we developed an in vitro assay for bryostatin-induced increased CD22 surface expression in ALL using NALM6 cells. Using this assay, we sought to determine whether synthetic bryostatin 1 and bryostatin analogs could achieve sufficient increase in CD22 surface density as required for CD22-targeted CAR T cell-mediated tumor clearance. NALM6 cells were incubated with bryostatin 1 and bryostatin analogs for 24 h, at which point compounds were washed out of the media and cells were analyzed for CD22 surface expression by flow cytometry. We found that bryostatin 1 induces a >2-fold increase in CD22 surface density (Fig. 6). Intriguingly, bryostatin-promoted increases in CD22 surface expression are sustained for up to 7 days following treatment (Fig. 6a), a finding consistent with recent work^31^. This further suggests that sequential administration of bryostatin 1 or a bryolog to enhance the CD22 target density followed by an anti-CD22 CAR T cell infusion could be a viable strategy for clearing CD22^lo^ tumor cells which appear to be driving patient relapse^11^.

Importantly, as C13-modified bryostatin analogs displayed a range of activities in our CD22 induction assay, select compounds proved highly effective and displayed activities comparable to bryostatin 1 (Fig. 6b). Surprisingly, we observed a pronounced effect in C13 enoate geometry on compound function in both the C13 methyl and allyl enoates (Fig. 6b). (*Z*)-enoates were considerably more active than the corresponding (*E*)-enoates, further suggesting that PKC signaling dynamics can be modulated by modifications to the B-ring of the bryostatin scaffold. As previously noted, top performer **SUW229** displays a different PKC activation profile relative to bryostatin 1, suggesting that it is possible that differential PKC activation dynamics can influence biologically significant downstream signaling outcomes.

Finally, to determine whether the results observed in NALM6 cells apply to other cell lines, we incubated the AIDS-related lymphoma cell lines JB and 2F7 cells with synthetic bryostatin 1, **SUW201** and **SUW229** (Fig. 6c, d). JB is an Epstein–Barr virus (EBV)-negative AIDS-lymphoma cell line originally grown out of a bone marrow sample derived from an HIV+ individual, which harbors the Burkitt lymphoma translocation^66^. 2F7 is an AIDS-associated non-Hodgkin’s lymphoma cell line of the Burkitt subtype, which is positive for EBV^67^. Burkitt lymphoma is one of the most common subtypes of AIDS non-Hodgkin’s lymphoma and along with Hodgkin’s lymphoma represents a significantly greater risk to AIDS patients relative to the general population due to their impaired cellular immunity^68^. Significantly, synthetic bryostatin 1 and analogs **SUW201** and **SUW229** upregulated CD22 surface expression by ~2-fold in each cell line tested (Fig. 6c, d), further highlighting the potential generality of using PKC modulators for enhancing CD22-targeted cancer immunotherapies.

## Discussion

In summary, we report the design, synthesis and biological evaluation of close-in analogs of bryostatin 1, a natural product of current clinical interest. Our step economical and scalable access to the bryostatin scaffold has provided sufficient material to enable a late-stage diversification strategy, leading to the next generation of bryostatin-derived lead compounds and highlighting the importance of chemical synthesis in natural product-inspired drug discovery. Using an FOS strategy, we designed and prepared a set of structurally diverse analogs and evaluated how variations in their structures influence their binding to PKC, their in vitro translocation of a PKC fusion protein, and their enhancement of CD22 antigen density in in vitro models of ALL and AIDS-related NHL. Significantly, while almost all analogs show bryostatin-like affinity to PKC in accord with our pharmacophore model, several exhibit notably different PKC isoform translocation kinetics and thus serve as potential leads for isoform-selective PKC modulation connected to different clinical indications and for the identification of potentially better tolerated leads. Of further potential clinical significance, these synthetic studies have enabled access to a class of promising leads for enhancing antigen-targeted immunotherapy, including enhanced CD22-targeted CAR T therapy. Given the towering importance of natural product-inspired analogs in human therapy, these compounds can serve as leads for further therapeutic studies across numerous indications that have thus far focused for decades principally on bryostatin 1 alone. More generally, this FOS approach calls attention to the use of nature’s treasure trove of natural products to inspire the design of potentially superior, better tolerated and synthetically more accessible agents for the prevention, diagnosis and treatment of disease.

## Methods

### Synthesis of bryostatin analogs

Experimental details for the synthesis of all previously unreported compounds, including experimental procedures, characterization and spectral data are provided in the Supplementary Information (Supplementary Methods).

### Biological evaluation of bryostatin analogs

Assay protocols for the PKC competitive binding assay, PKC-GFP translocation assay and CD22 expression assays are also provided in the Supplementary Information (Supplementary Methods).

### Reporting summary

Further information on research design is available in the Nature Research Reporting Summary linked to this article.

## Supplementary information

Supplementary Information

Reporting Summary

## Data Availability

All data supporting the findings of this study are available within the Article and its Supplementary Information, or from the corresponding author upon request. Raw data associated with Figs. 5 and 6 are also available upon request.
