# Supplementary material for: Synthesis and evaluation of designed PKC modulators for enhanced cancer immunotherapy
Source: Nat Commun. 2020 Apr 20;11:1879. doi: 10.1038/s41467-020-15742-7 (PMC7170889; doi:10.1038/s41467-020-15742-7)
Supplement: Supplementary file 1 — Supplementary Information [file 41467_2020_15742_MOESM1_ESM.pdf]

SUPPLEMENTARY INFORMATION

Synthesis and Evaluation of Designed PKC Modulators for Enhanced Cancer Immunotherapy

Authors: Clayton Hardman[1], Stephen Ho[1], Akira Shimizu[1], Quang Luu-Nguyen[1], Jack L. Sloane[1], Mohamed S. A. Soliman[2], Matthew D. Marsden[3*], Jerome A. Zack[2,3*], Paul A. Wender[1*]

[1]Departments of Chemistry and of Chemical and Systems Biology, Stanford University, Stanford, California, 94305

[2]Department of Microbiology, Immunology, and Molecular Genetics, University of California Los Angeles, Los Angeles, California, 90095

[3]Department of Medicine, Division of Hematology and Oncology, University of California Los Angeles, Los Angeles, California, 90095

Table of Contents

Supplementary Methods – 2

Supplementary Figures – 58

Supplementary Methods

All reactions were conducted in oven- or flame-dried glassware under a nitrogen or argon atmosphere unless otherwise noted. Reactions were concentrated under reduced pressure with a rotary evaporator unless otherwise noted. Commercial reagents were used as received or purified using the methods indicated herein. Dichloromethane, diethyl ether, dimethylformamide, pentane, tetrahydrofuran, and toluene were passed through an alumina-drying column (Solv-Tek Inc.) using nitrogen pressure; ethyl acetate, hexanes, and petroleum ether were obtained from Fisher Scientific. Analytical thin-layer chromatography (TLC) was carried out on 250 µm silica gel 60G plates with fluorescent indicator F254 (EMD Millipore). Plates were visualized with UV light and treated with *p*-anisaldehyde, ceric ammonium molybdate, or potassium permanganate stain with gentle heating. Flash column chromatography was performed using silica gel (230-400 mesh, grade 60, particle size 40 to 63 µm) purchased from Fischer Scientific. pH 7 buffered silica gel was prepared by adding 10% weight pH 7 phosphate buffer to silica and rotating for ~12 hrs. NMR spectra were acquired on a Varian INOVA 600, Varian INOVA 500, or Varian 400 magnetic resonance spectrometer. ^{1}H chemical shifts are reported relative to the residual solvent peak ($CHCl_3$ = 7.26 ppm, C_6H_6 = 7.16 ppm) as follows: chemical shift (δ), multiplicity (app = apparent, b = broad, s = singlet, d = doublet, t = triplet, q = quartet, m = multiplet, or combinations thereof), coupling constant(s) in Hz, integration. ^{13}C chemical shifts are reported relative to the residual solvent peak ($CHCl_3$ = 77.16 ppm, C_6H_6 = 128.06 ppm). Infrared spectra were acquired on a Nicolet iS 5 FT-IR Spectrometer (ThermoFisher). Optical rotations were acquired on a P-2000 Digital Polarimeter (Jasco). High-resolution mass spectra (HRMS) were acquired at the Vincent Coates Foundation Mass Spectrometry Laboratory at Stanford.

SUW200

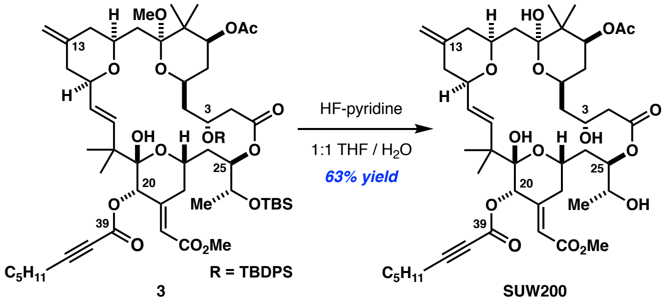

To a 15mL polypropylene conical centrifuge tube equipped with magnetic stir bar was added compound **3** (15 mg, 0.0124 mmol, 1 equiv) and 3:1 THF / H_2O (1 mL). The centrifuge tube was transferred to a 4 °C cold room. HF-pyridine (0.32 mL) was added (final concentration ~0.01M). After 96h, the reaction mixture was warmed to room temperature. After an additional 64h (~6.5 days in total), the reaction mixture was quenched by slowly syringing the solution into a separatory funnel containing saturated aqueous $NaHCO_3$ (20mL) and EtOAc (20 mL). The layers were separated, and the aqueous layer was extracted with EtOAc (4x20mL). The combined organic layers were washed with 0.5M HCl (10 mL) to remove pyridine, and aqueous layer back-extracted with EtOAc (2x20mL). The combined organic layers were dried over Na_2SO_4, filtered, and concentrated. Purification was accomplished by silica gel flash column chromatography (25-65% EtOAc/Hex) affording **SUW200** (6.6 mg, 63% yield) as a white solid. Compound purity was established by TLC (one spot) analysis.

Characterization data for SUW200

TLC R_f = 0.56 (60% EtOAc/Hex, UV active, dark purple spot in *p*-anisaldehyde)

$[\alpha]^{22.3}_D$ = -1.8° (*c* = 0.18, CH_2Cl_2); **IR** (thin film): 3460, 3331, 2929, 2855, 2234, 1738, 1716, 1666, 1435, 1408, 1366, 1244, 1157, 1098, 1055, 982 cm^{-1}

^{1}H-NMR (600 MHz, $CDCl_3$) δ 5.98 (d, *J* = 2.0 Hz, 1H, $C_{34}H$), 5.77 (d, *J* = 15.8 Hz, 1H, $C_{17}H$), 5.30 (dd, *J* = 15.8, 8.6 Hz, 1H, $C_{16}H$), 5.28 (s, 1H, C_{19}-OH), 5.21 (ddd, *J* = 12.0, 5.5, 3.0 Hz, 1H, $C_{25}H$), 5.16 (dd, *J* = 11.9, 4.8 Hz, 1H, C_7H), 5.14 (s, 1H, $C_{20}H$), 4.76 (bs, 1H, $C_{30}H_a$), 4.74 (bs, 1H, $C_{30}H_b$), 4.27 (d, *J* = 12.1 Hz, 1H, C_3-OH), 4.27 – 4.19 (m, 1H), 4.20 – 4.13 (m, 1H), 4.07 – 3.99 (m, 2H), 3.85 – 3.76 (m, 1H), 3.72 – 3.67 (m, 1H, $C_{22}H_a$), 3.68 (s, 3H, CO_2Me), 3.66 – 3.61 (m, 1H), 2.50 (dd, *J* = 12.5, 2.4 Hz, 1H, C_2H_a), 2.45 (app. t, *J* = 12.0 Hz, 1H, C_2H_b), 2.35 (s, 1H, C9-OH), 2.32 (t, *J* = 7.2 Hz, 2H, $C_{42}H_2$), 2.14 – 2.05 (m, 5H), 2.05 (s, 3H, C7-OAc), 2.03 – 1.90 (m, 4H), 1.86 (ddd, *J* = 14.0, 11.6, 3.0 Hz, 1H, $C_{24}H_b$), 1.77 (ddd, *J* = 12.2, 4.6, 2.7 Hz, 1H, C_6H_{eq}), 1.67 (d, *J* = 15.1 Hz, 1H, $C_{10}H_b$), 1.64 – 1.55 (m, 3H, C_4H_b, $C_{43}H_2$), 1.47 (app. q, *J* = 12.0 Hz, 1H, C_6H_b), 1.41 – 1.28 (m, 4H, $C_{44}H_2$, $C_{45}H_2$), 1.22 (d, *J* = 6.5 Hz, 3H, $C_{27}H_3$), 1.20 (s, 3H), 1.01 (s, 3H), 1.00 (s, 3H), 0.95 (s, 3H), 0.90 (t, *J* = 7.2 Hz, 3H, $C_{46}H_3$)

^{13}C-NMR (125 MHz, $CDCl_3$) δ 172.3, 170.9, 167.0, 152.4, 151.1, 143.5 (C13), 138.8, 130.0, 120.4, 109.2 (C30), 102.0, 98.9, 91.3 (C41), 80.2, 75.4, 73.8, 73.1, 72.9, 72.1, 70.3, 68.7, 65.9, 64.8, 51.3, 45.0, 42.8, 42.6, 42.1, 41.3, 41.1, 40.1, 35.8, 33.5, 31.3, 31.1 (C44), 29.9, 27.3 (C43), 24.6, 22.2(C45), 21.3, 21.2, 19.9, 18.9 (C42), 17.0, 14.1 (C46)

HRMS calculated for $C_{45}H_{66}NaO_{15}$ $[M+Na]^+$: 869.4294; found 869.4290 (TOF ESI+).

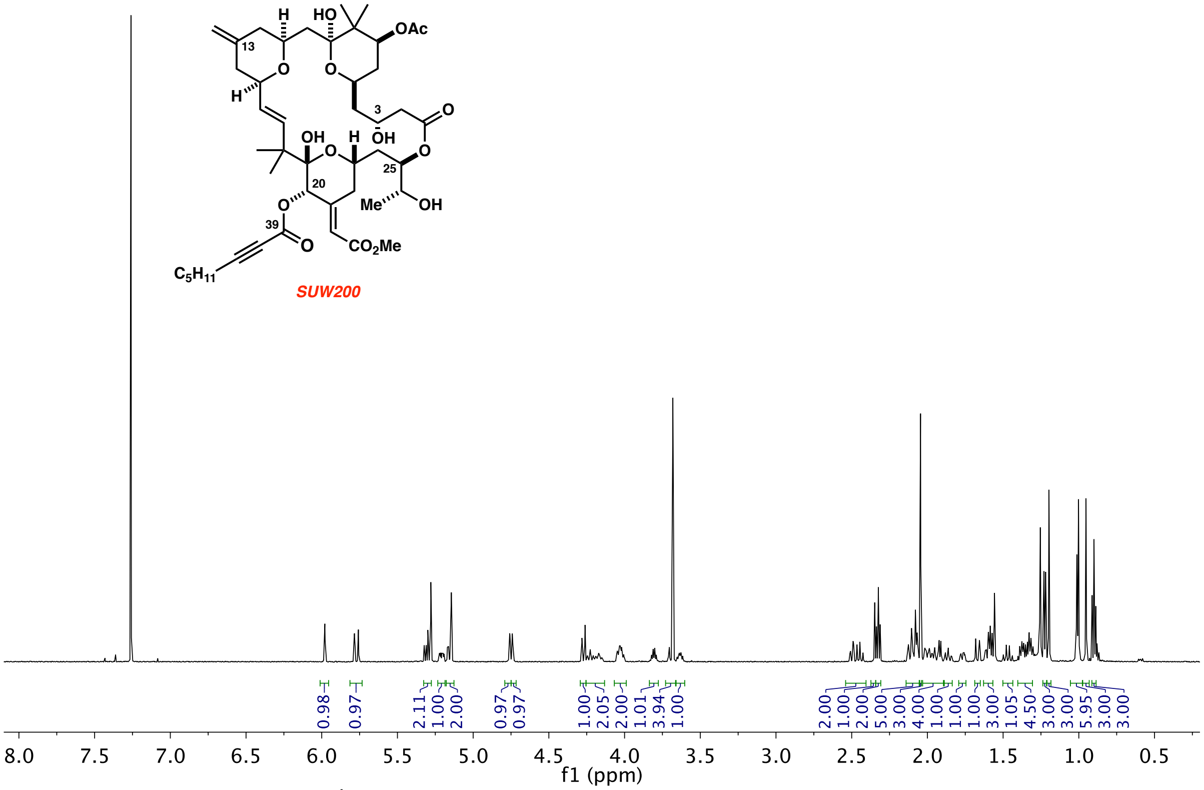

Supplementary Figure 1. ^{1}H-NMR (600 MHz, $CDCl_3$) of SUW200.

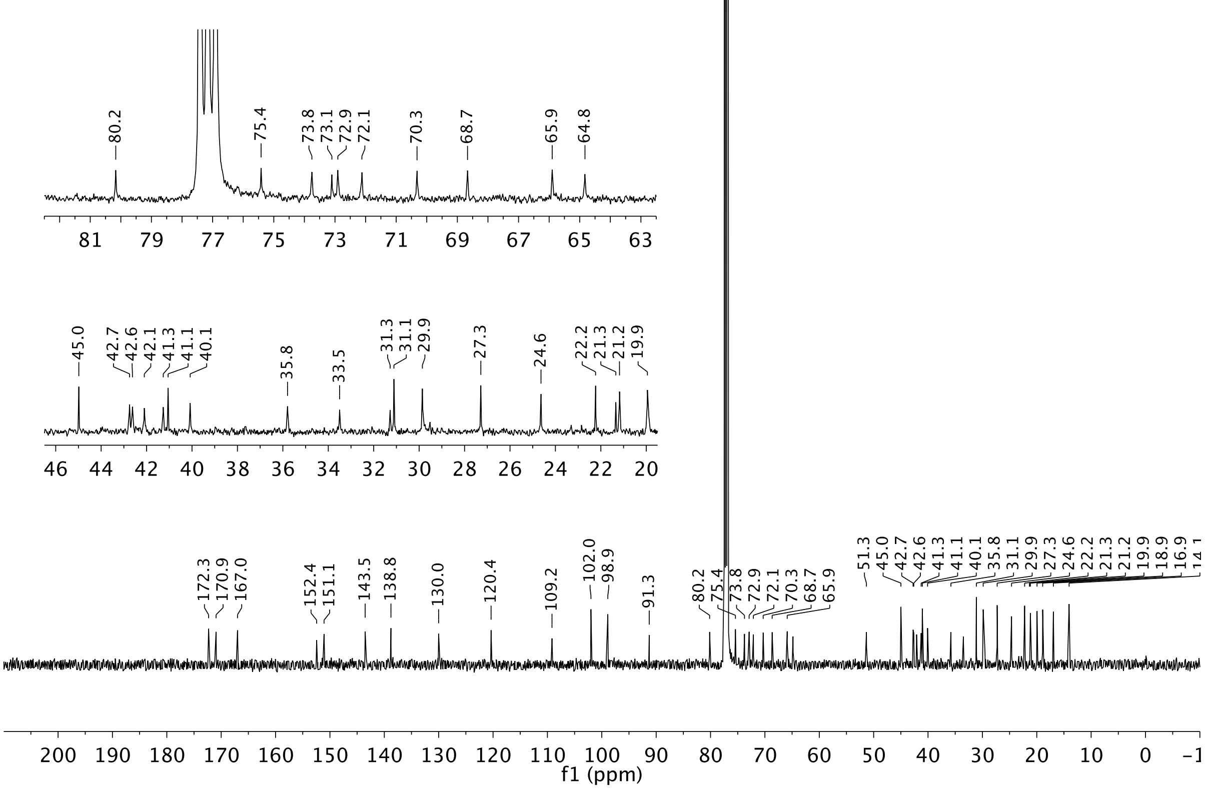

Supplementary Figure 2. ^{13}C-NMR (125 MHz, $CDCl_3$) of SUW200.

SUW203

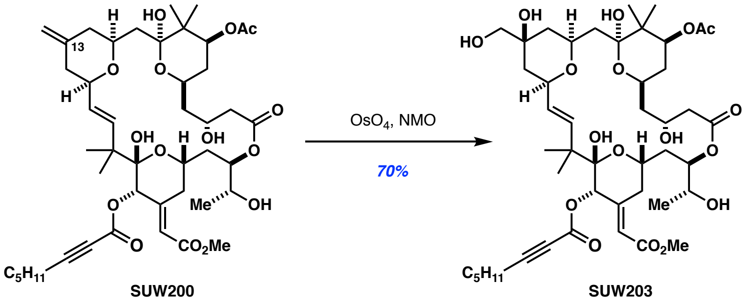

To a 1-dram vial was added **SUW200** (6.6 mg, 0.0078 mmol, 1 equiv) in acetone (0.27 mL). NMO (1.4 mg, 0.0117 mmol, 1.5 equiv) and OsO_4 (30 µL of a 0.01 M solution in water, 0.00031 mmol, 0.04 equiv) were added sequentially and the resulting mixture was stirred for 24 h. The reaction mixture was diluted with EtOAc and sat. $Na_2S_2O_3$ and extracted with EtOAc (2x). The combined organic layers were dried over Na_2SO_4 and concentrated to a white paste. Purification by flash chromatography (pipette column, 5 – 10% MeOH/DCM) gave diol **SUW203** as a white powder (4 mg, 60% yield).

TLC: R_f = 0.1 (80% EtOAc/hexane, UV active, purple spot in *p*-anisaldehyde)

$[\alpha]^{23.9}_D$ = -9.8° (*c* = 0.14, CH_2Cl_2)

IR (thin film): 3447, 2928, 2858, 2234, 1735, 1718, 1654, 1412, 1366, 1245, 1158, 1106, 1080, 1063 cm^{-1}

^{1}H NMR (600 MHz, $CDCl_3$) δ 5.96 (s, 1H), 5.71 (d, *J* = 15.9 Hz, 1H), 5.26 – 5.18 (m, 2H), 5.12 (m, 3H), 4.22 (t, *J* = 11.9 Hz, 1H), 4.14 (m, 1H), 4.06 (m, 1H), 3.99 (m, 2H), 3.86 – 3.76 (m, 2H), 3.75 (m, 1H), 3.68 – 3.66 (m, 4H), 2.49 (t, *J* = 14.7 Hz, 2H), 2.30 (t, *J* = 7.2 Hz, 2H), 2.05 – 1.94 (m, 6H), 1.92 – 1.51 (m, 22H), 1.48 – 1.34 (m, 6H), 1.33 – 1.26 (m, 6H), 1.23 (s, 3H), 1.21 (d, *J* = 6.4 Hz, 3H), 1.16 (s, 3H), 0.98 (s, 3H), 0.97 (s, 3H), 0.91 (s, 3H), 0.87 (m 6H).

^{13}C NMR (126 MHz, $CDCl_3$) δ 167.13, 151.20, 144.87, 142.48, 128.74, 128.66, 126.07, 120.58, 110.77, 102.10, 99.06, 95.85, 75.47, 73.27, 70.47, 68.81, 68.61, 68.18, 66.26, 66.01, 64.91, 51.44, 46.96, 45.11, 42.31, 41.85, 41.33, 40.14, 38.98, 32.46, 31.25, 30.61, 30.26, 27.43, 26.95, 24.74, 22.98, 22.36, 21.46, 21.29, 19.05, 17.15, 14.41, 14.17.

HRMS calculated for $C_{45}H_{68}O_{17}$ [M+Na]+: 903.4349; found 903.4334 (TOF ESI+)

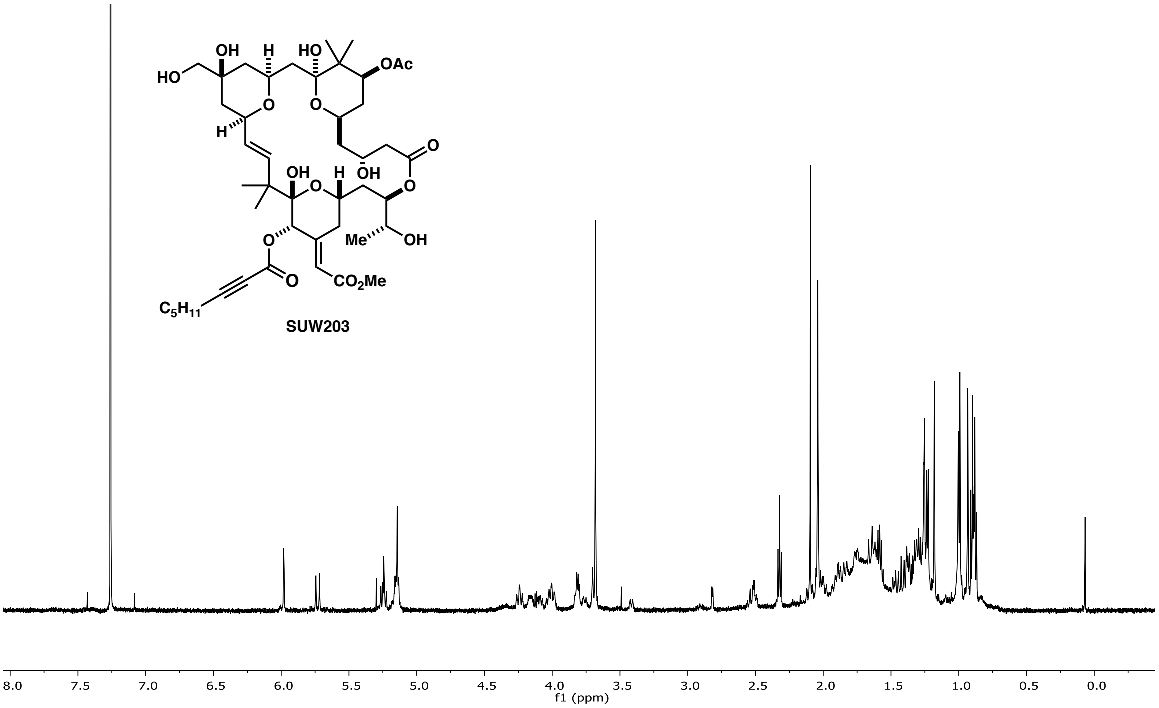

Supplementary Figure 3. ^{1}H NMR (600 MHz, $CDCl_3$) of SUW203.

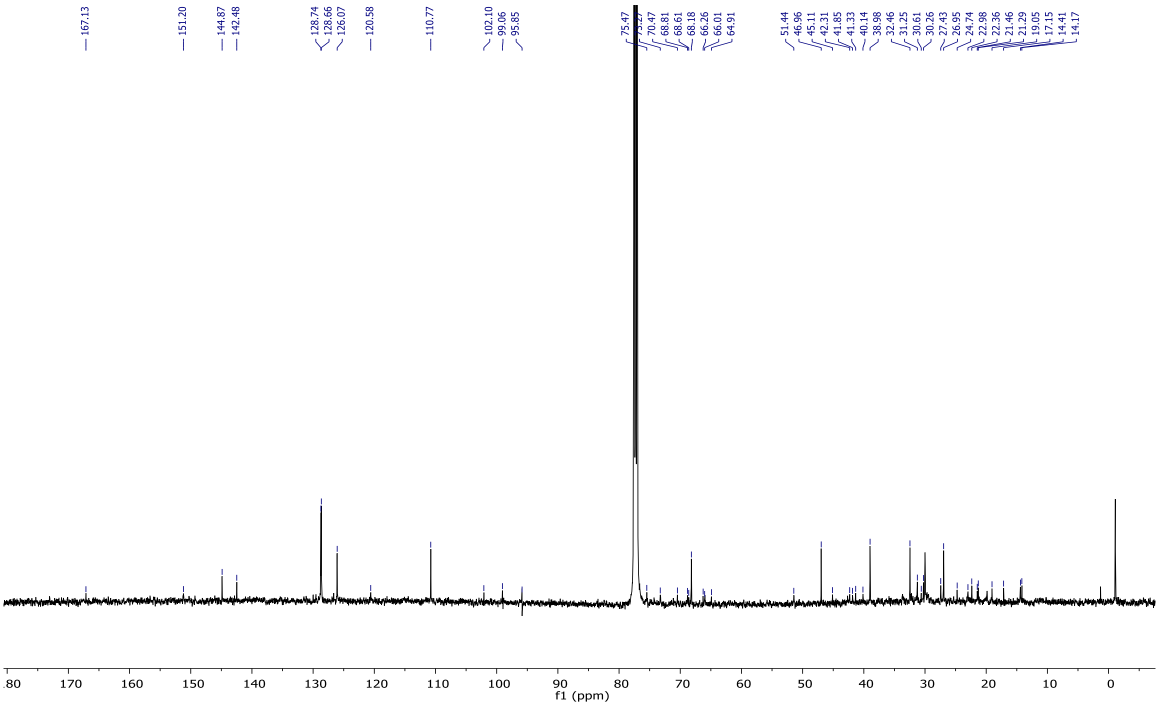

Supplementary Figure 4. ^{13}C NMR (126 MHz, $CDCl_3$) of SUW203.

SUW226

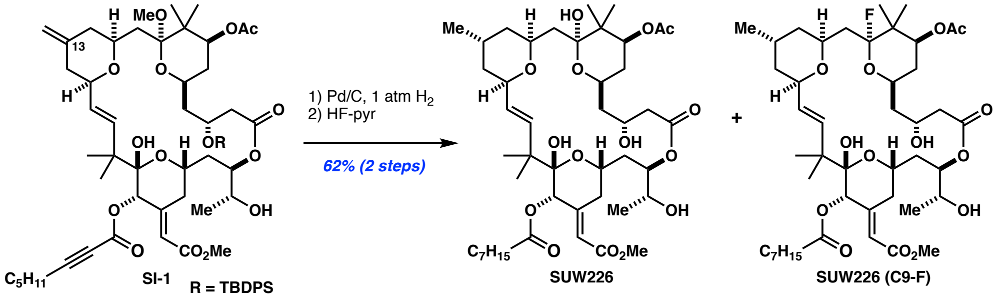

Hydrogenation: To an 8-dram vial equipped with magnetic stir bar was added Prins product **SI-1** (*Science*, **2017**, *358*, 218 – 223) (50 mg), EtOAc (500 uL), and palladium on carbon (50 mg). The vial was placed under a hydrogen atmosphere (balloon). After 2h, TLC analysis indicated complete conversion of starting material. The reaction mixture was filtered over a pipette containing celite and directly concentrated. Purification was accomplished by silica gel flash column chromatography (25% EtOAc/pentane) affording 40 mg of material.

Global deprotection: To a cooled (0 °C) solution of this material (40 mg) in 1:1 THF / pyridine (800 uL) was added 70% HF-pyridine (400 uL), affording a 1:2:2 HF-pyr / THF / pyridine solution. The reaction mixture was allowed to warm to room temperature. After 36h, H_2O (400 uL) was added, and the reaction mixture was heated to 40 °C. After 3h at 40 °C, the reaction mixture was cooled to 0 °C, diluted with EtOAc (10 mL), and slowly quenched by adding saturated aqueous $NaHCO_3$ dropwise until bubbling ceased. The layers were separated, and the aqueous layer was extracted with 80% EtOAc/Hex (5x10 mL). The combined organic layers were dried over $NaSO_4$, filtered, and concentrated. Purification was accomplished by silica gel flash column chromatography (33-67% EtOAc/Hex) affording a mixture of **SUW226** and its C9-fluoride (24 mg, 62% combined yield over 2 steps). 15 mg of this diastereomeric mixture was then subjected to RP-HPLC (70-100% MeCN/H_2O) affording pure samples of **SUW226** and its C9-fluoride. The stereochemistry at C13 was assigned from 2D-ROESY data (cross-peaks observed between $C_{13}CH_3$ and $C_{11}H$, and between $C_{13}CH_3$ and $C_{15}H$).

Characterization data for SUW226

TLC R_f = 0.31 (50% EtOAc/Hex, UV active, dark purple spot in *p*-anisaldehyde)

$[\alpha]^{23}_D$ = 18.2° (*c* = 0.18, CH_2Cl_2)

IR (thin film): 3450 (bs), 2928, 1738, 1720, 1437, 1366, 1237, 1157, 1099, 1060, 1002 cm^{-1}

^{1}H-NMR (600 MHz, $CDCl_3$) δ 6.46 (d, *J* = 2.0 Hz, 1H, $C_{34}H$), 6.24 (d, *J* = 15.8 Hz, 1H, $C_{17}H$), 5.84 (s, 1H, C_{19}-OH), 5.74 (s, 1H, $C_{20}H$), 5.55 (dd, *J* = 15.8, 8.2 Hz, 1H, $C_{16}H$), 5.40 – 5.36 (m, 1H, $C_{25}H$), 5.35 (dd, *J* = 11.9, 4.9 Hz, 1H, C_7H), 4.72 – 4.65 (m, 1H, $C_{15}H$), 4.62 (d, *J* = 12.3 Hz, 1H, C_3-OH), 4.53 – 4.45 (m, 1H, $C_{23}H$), 4.34 (dd, *J* = 13.6, 2.2 Hz, 1H, $C_{22}H_{eq}$), 4.12 (app. t, *J* = 11.6 Hz, 1H, C_3H), 4.08 – 4.01 (m, 1H, $C_{11}H$), 3.90 – 3.82 (m, 1H, C_5H), 3.68 (app. p, *J* = 6.3 Hz, 1H, $C_{26}H$), 3.21 (s, 3H), 2.48 – 2.37 (m, 2H, C_2H_a, $C_{22}H_{ax}$), 2.20 – 2.10 (m, 3H, C_2H_b, $C_{40}H_2$), 2.07 (dd, *J* = 15.0, 7.1 Hz, 1H, $C_{10}H_a$), 1.94 – 1.87 (m, 1H, $C_{13}H$), 1.81 – 1.70 (m, 2H, $C_{24}H_2$), 1.68 (s, 3H), 1.55 (s, 3H), 1.28 (s, 3H), 1.24 (d, *J* = 7.3 Hz, 3H, $C_{13}CH_3$), 1.00 (d, *J* = 6.4 Hz, 3H, $C_{27}H_3$), 0.93 (s, 3H), 0.90 (s, 3H), 0.86 (t, *J* = 7.2 Hz, 3H, $C_{46}H_3$)

^{13}C-NMR (125 MHz, C_6D_6) δ 172.9, 171.8, 170.0, 166.8, 152.7, 138.6, 131.2, 120.5, 102.2, 99.8, 75.0, 74.4, 74.0, 73.0, 70.4, 68.8, 65.7, 65.5, 65.2, 50.6, 45.3, 42.8, 42.6, 41.2, 39.9, 39.8, 38.2, 36.2, 34.8, 33.7, 32.1, 32.0, 29.33, 29.27, 26.4, 25.4, 25.1, 23.0, 21.2, 20.7, 20.0, 19.8, 18.0, 17.0, 14.3

HRMS calculated for $C_{45}H_{72}NaO_{15}$ $[M+Na]^+$: 875.4764; found 875.4737 (TOF ESI+).

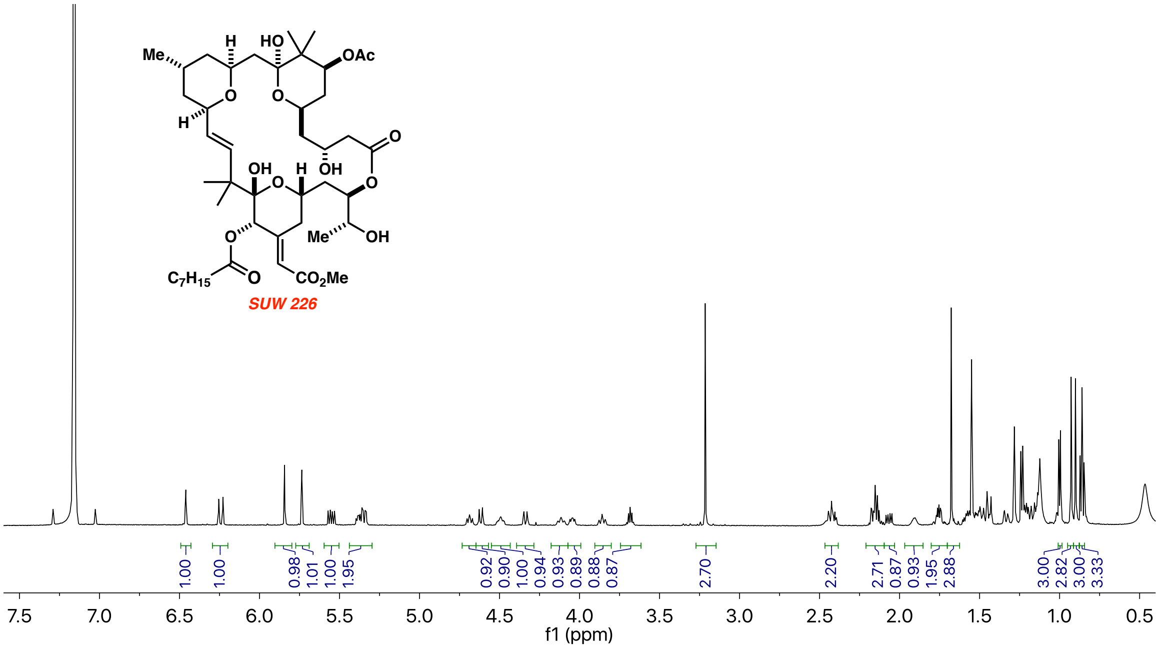

Supplementary Figure 5. ^{1}H NMR (600 MHz, C_6D_6) of SUW226.

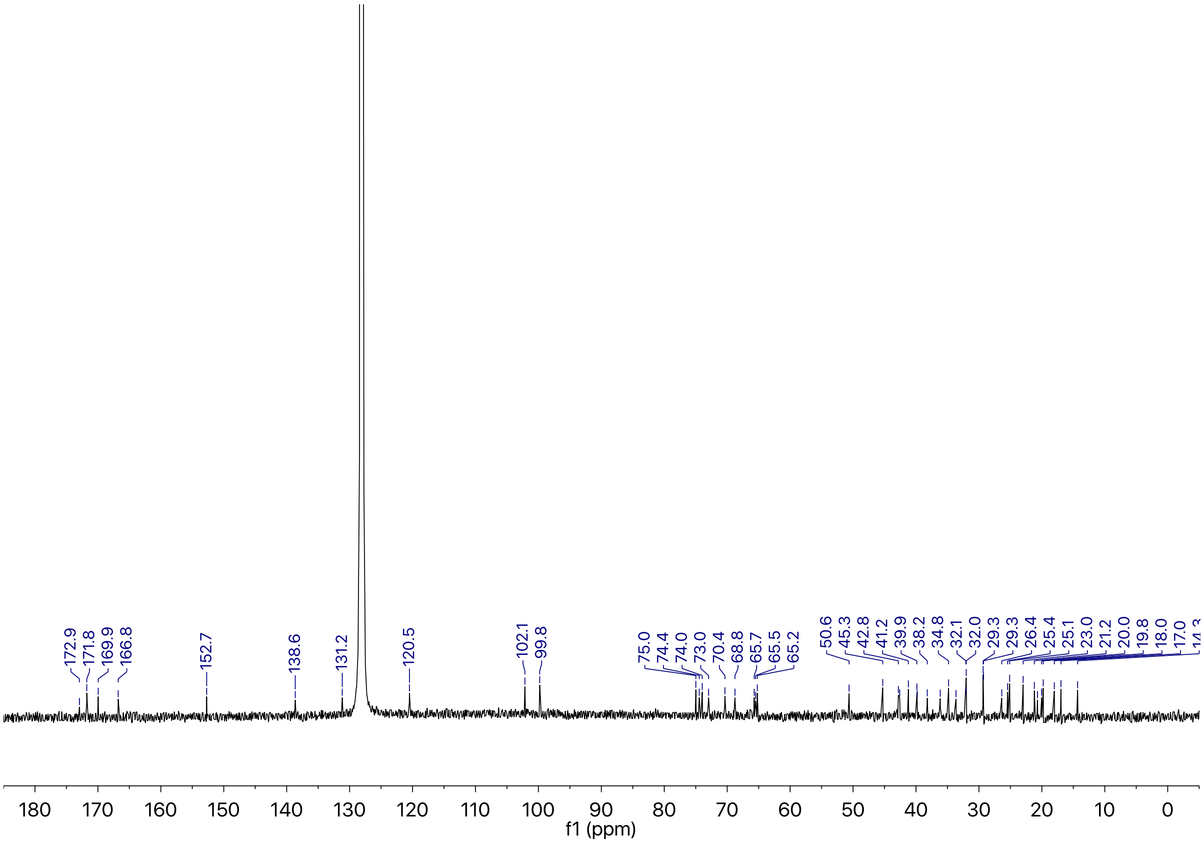

Supplementary Figure 6. ^{13}C-NMR (125 MHz, C_6D_6) of SUW226.

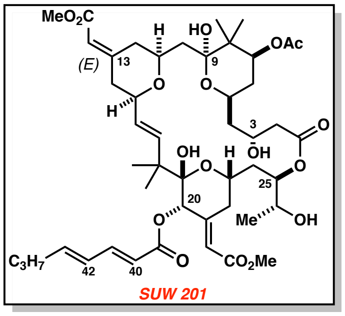

SUW201 is the C13 (*E*)-enoate isomer of bryostatin 1. Because the HWE olefination produces a ~10:1 mixture of C13 isomers, **SUW201** can be separated from bryostatin 1 via RP-HPLC. See *Science*, **2017**, *358*, 218-223 for synthesis and purification conditions.

[α]$^{23.2}_{D}$ = -35.5° (*c* = 0.26, CH_2Cl_2)

IR (thin film): 3464, 3336, 2951, 2928, 1716, 1657, 1643, 1615, 1435, 1408, 1366, 1284, 1242, 1156, 1098, 1078, 1057, 1002, 859 cm^{-1}

^{1}H-NMR (500 MHz, $CDCl_3$) δ 7.30 – 7.23 (dd obscured by chloroform peak, 1H, $C_{41}H$), 6.21 – 6.11 (m, 2H, $C_{42}H$, $C_{43}H$), 6.00 (d, *J* = 1.9 Hz, 1H, $C_{34}H$), 5.81 (d, *J* = 15.8 Hz, 1H), 5.80 (d, *J* = 15.3 Hz, 1H), 5.71 (s, 1H, $C_{30}H$), 5.30 (dd, *J* = 15.9, 8.4 Hz, 1H, $C_{16}H$), 5.24 (s, 1H, C_{19}-OH), 5.24 – 5.18 (m, 1H, $C_{25}H$), 5.20 (s, 1H, $C_{20}H$), 5.15 (dd, *J* = 11.8, 4.7 Hz, 1H, C_7H), 4.27 (d, *J* = 12.1 Hz, 1H, C_3-OH), 4.28 – 4.20 (m, 1H, C_5H), 4.21 – 4.09 (m, 2H, C_3H, $C_{15}H$), 4.02 (app. t, *J* = 11.4 Hz, 1H, $C_{23}H$), 3.86 – 3.78 (m, 1H, $C_{11}H$), 3.79 – 3.73 (m, 1H, $C_{26}H$), 3.74 – 3.67 (m, 2H, $C_{22}H_{eq}$, $C_{14}H_{eq}$), 3.69 (s, 3H, CO_2Me), 3.66 (s, 3H, CO_2Me), 2.67 (bs, 1H, C_9-OH), 2.53 – 2.38 (m, 2H, C_2H_2), 2.19 – 1.90 (m, 10H), 2.05 (s, 3H, C7-OAc), 1.86 – 1.79 (m, 1H, $C_{24}H_b$), 1.79 – 1.72 (m, 2H, C_6H_{eq}, $C_{10}H_b$), 1.63 – 1.56 (m, 1H, C_4H_b), 1.54 – 1.41 (m, 3H, C_6H_{ax}, $C_{45}H_2$), 1.23 (d, *J* = 6.5 Hz, 3H, $C_{27}H_3$), 1.14 (s, 3H), 1.004 (s, 3H), 1.001 (s, 3H), 0.95 (s, 3H), 0.92 (t, *J* = 7.4 Hz, 3H, $C_{46}H_3$)

^{13}C-NMR (125 MHz, $CDCl_3$) δ 172.4, 171.0, 167.2, 167.1, 165.7, 156.9, 152.0, 146.5, 145.7, 139.7, 129.2, 128.5, 119.8, 118.7, 114.5, 101.9, 99.1, 79.9, 74.2, 73.7, 73.0, 71.5, 70.3, 68.7, 65.9, 64.8, 51.2 (2C), 45.0, 43.2, 42.5, 42.2, 41.2, 40.0, 37.6, 35.9, 35.2, 33.5, 31.4, 24.7, 22.0, 21.3, 21.2, 19.9, 19.8, 17.0, 13.8

HRMS calculated for $C_{47}H_{68}NaO_{17}$ $[M+Na]^+$: 927.4349; found 927.4338 (TOF ESI+)

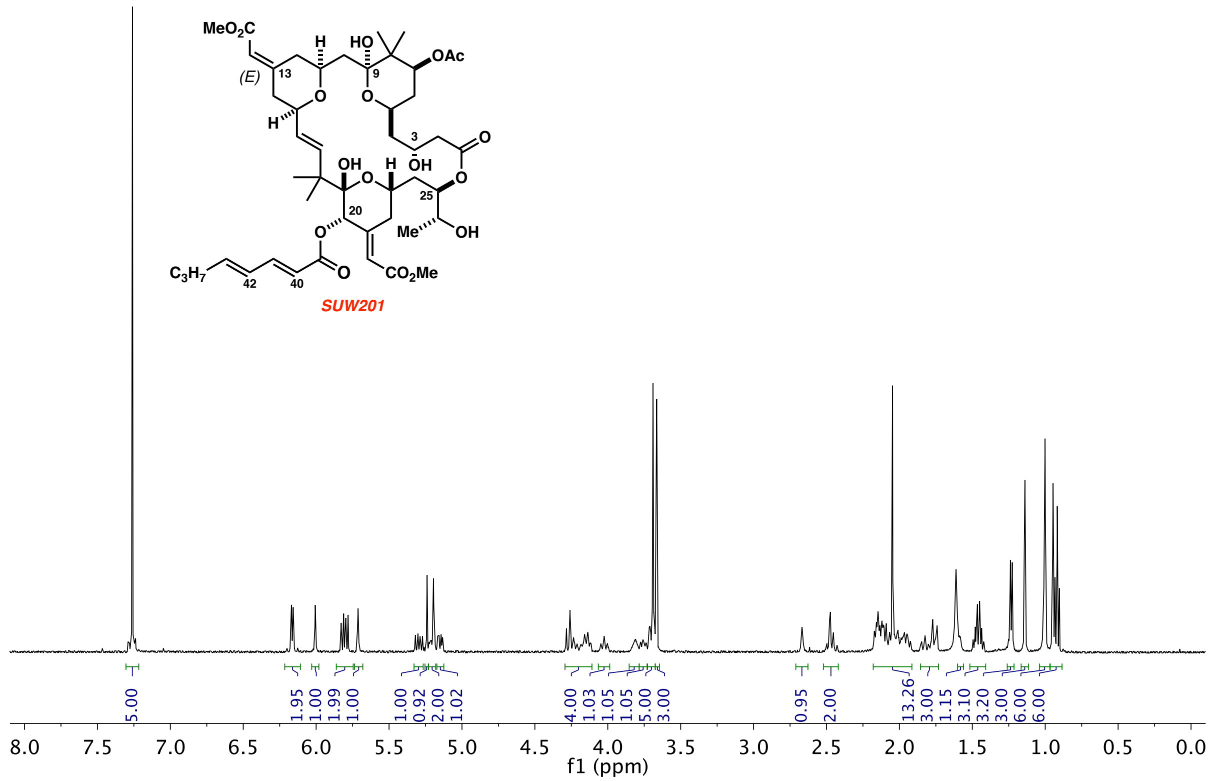

Supplementary Figure 7. ^{1}H-NMR (500 MHz, $CDCl_3$) of SUW201.

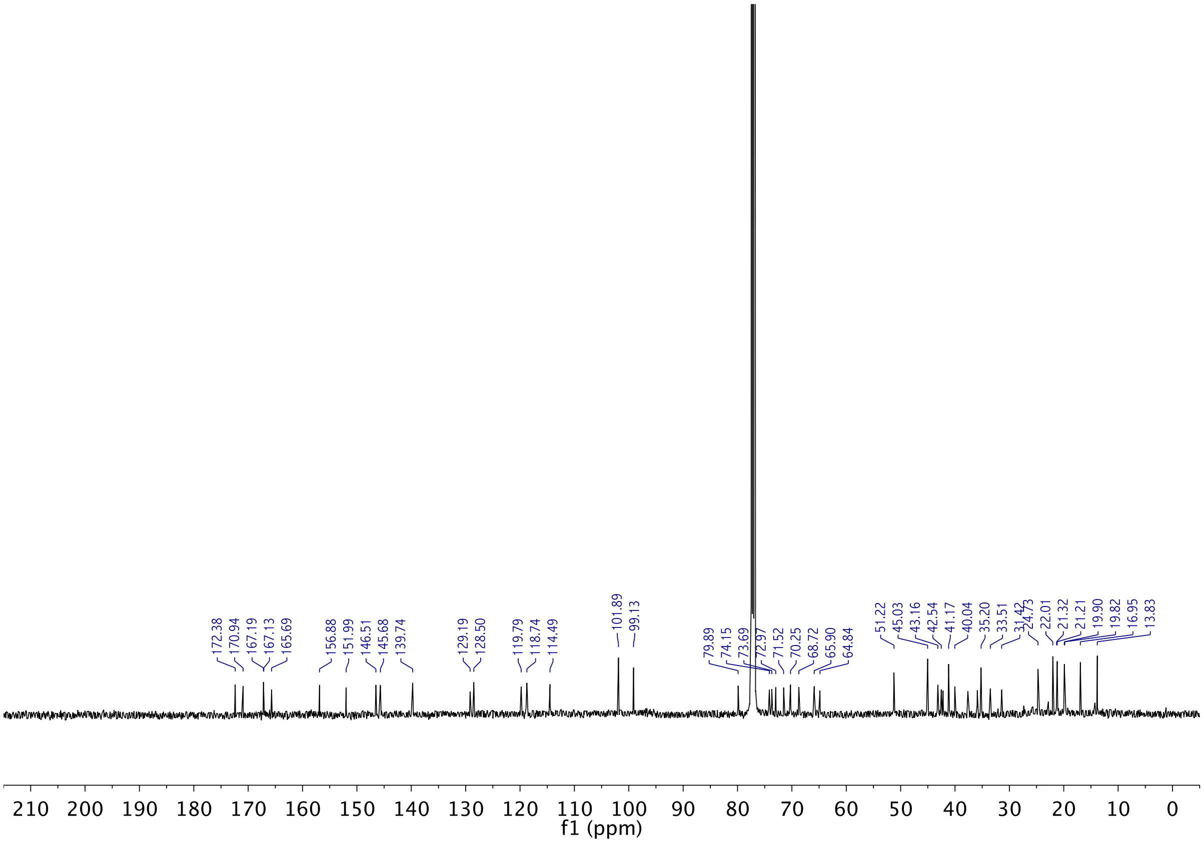

Supplementary Figure 8. ^{13}C-NMR (125 MHz, $CDCl_3$) of SUW201.

Synthesis of modified C13 enoates: HWE olefination of C13 ketone

Preparation of HWE reagents from diethylphosphonoacetic acid:

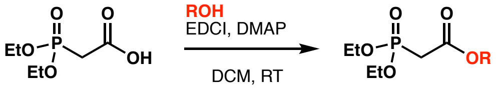

Chemicals:
Diethylphosphonoacetic acid (Aldrich, used without purification)
DMAP (Aldrich): recrystallized from hexanes
EDCI (Chem-Impex): used without purification

To a flame-dried 8-dram vial equipped with magnetic stir bar was added diethylphosphonoacetic acid (1.0 equiv) in DCM (~0.2 M). The corresponding alcohol (1.1 equiv) was added, followed by DMAP (0.5 equiv) in a single portion. EDCI (2.0 equiv) was then added in a single portion and the reaction was stirred at RT for 30 minutes, at which point TLC indicated complete conversion of starting material. The reaction mixture was directly diluted with water and extracted with 3 x EtOAc. The combined organic layers were washed with brine and dried over Na_2SO_4, filtered, and concentrated. The crude residue was purified by silica gel flash chromatography (80-90% EtOAc in hexanes) to afford the desired phosphonate, which was then used in the subsequent HWE olefination. Compound purity was established by TLC (one spot) analysis. Characterization data matched literature values reported by Lloyd *et al*. (allyl phosponate, *Organic and Biomolecular Chemistry* **2016**, *14*, 8971 – 8988.) and O'Leary *et al.* (benzyl phosphonate, *JACS* **2001**, *123*, 11519-33).

Note that exact amounts of phosphonates prepared varied, but procedure was generally carried out on a ~200 mg scale.

Synthesis of modified C13 enoates: HWE olefination of C13 ketone

HWE olefination of C13 ketone:

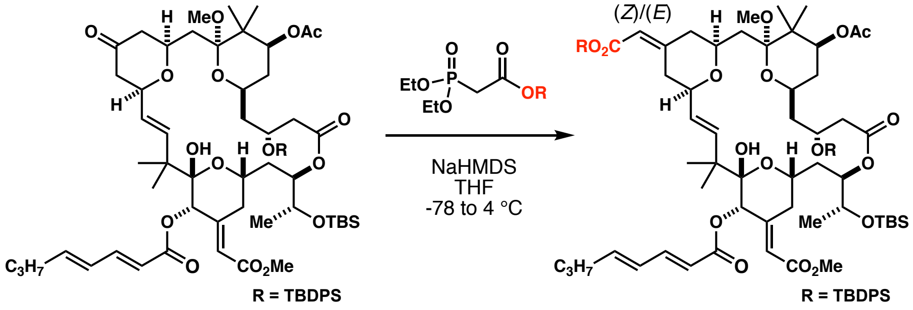

4 7

Chemicals:
NaHMDS (1M in THF, Aldrich): used without purification
Triethylphosphonate (Aldrich): used without purification
Alternative phosphoates prepared via procedure presented above

To a flame-dried 8-dram vial equipped with a magnetic stir bar was added the corresponding phosphonate (azeotroped in benzene x 2) in THF. The solution was cooled to 0 °C and NaHMDS was added dropwise. The reaction mixture was allowed to stir at 0 °C for 30 minutes, at which point it became bright yellow. Separately, ketone **4** (azeotroped in benzene x 2) was added to a flame-dried 8-dram vial equipped with a magnetic stir bar in THF. The solution was cooled to -78 °C and an aliquot of the deprotonated phosphonate was added dropwise as a solution in THF. The reaction was allowed to stir at -78 °C for 5 minutes, at which point it was transferred to a cold room and stirred at 4 °C for ~2 hours, after which TLC indicated complete consumption of starting material. The reaction mixture then was pipetted in to sat. aq. NH_4Cl and extracted with 3 x Et_2O. The combined organic layers were dried over Na_2SO_4, filtered, and concentrated. The crude residue was purified by silica gel flash chromatography (15-25% EtOAc in hexanes; TLC R_f = 0.52 in 60% EtOAc/hexanes, purple spot *p*-anisaldehyde) to afford the desired C13 enoates as a mixture of (*Z*) and (*E*) isomers which are inseparable on silica gel. The mixture of geometric isomers was moved directly in to the subsequent global deprotection.

Synthesis of modified C13 enoates: Global Deprotection

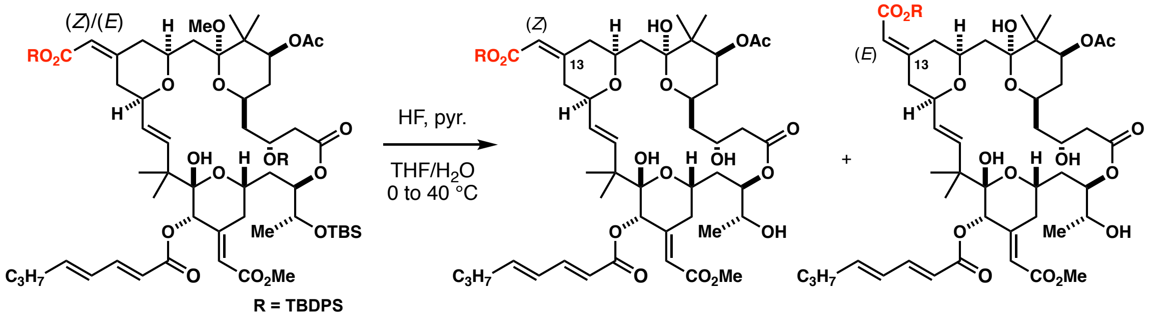

7

Chemicals
70% HF-pyridine (Sigma-Aldrich): used without purification
Pyridine (Sigma-Aldrich): distilled from CaH_2 before use

To a 15 mL polypropylene falcon tube equipped with a magnetic stir bar was added enoate **7** in THF. The solution was cooled to 0 °C and pyridine was added dropwise. HF-pyridine was then added dropwise, affording a 0.0075 M solution of enoate **7** in a 1:2:2 mixture of HF-pyridine/THF/pyridine, and the resulting reaction mixture was directly transferred to a 40 °C oil bath. The reaction was allowed to stir for 20 hrs., at which point H_20 (equal volume to HF-pyridine) was added dropwise. The reaction mixture was allowed to stir for and additional 2 hrs at 40 °C. The reaction mixture was then directly syringed in to the aqueous layer of a pre-chilled mixture of EtOAc/sat. aq. $NaHCO_3$. The aqueous layer was extracted with 3 x EtOAc and the combined organic layers were washed with 1 M HCl followed by brine. The combined organic layers were then dried over Na_2SO_4, filtered, and concentrated. The product was purified by silica gel flash chromatography (10-35-45-50-60% EtOAc in hexanes), affording a mixture of C13 geometric isomers, which were subsequently separated by HPLC.

Preparative HPLC

~1:1 Z:E mixture of C13 enoate isomers was further purified by preparative HPLC using a Shimadzu LC-20AP Prominence preparative liquid chromatograph system with detectors set to 254 and 280 nm. Separations were performed using a Restek Ultra C18 column (5 µm particle size, 250 mm x 10 mm). The mobile phase was a gradient elution from 75% MeCN/H2O to 100% MeCN/H2O over 30 min, followed by 100% MeCN for 10 min (flow rate of 5 mL/min). The sample was dissolved in 1:1 MeCN/MeOH for loading. Two fractions were collected, affording diastereomerically pure samples of the (*Z*)- and (*E*)-enoate isomers as a fluffy white powder (fraction 1: (*Z*) enoate, fraction 2: (*E*) enoate). Sterochemistry of the C13 enoate was confirmed by ROESY NMR on SUW218 (more details below). Additionally, HPLC retention times and relative C30 1H chemical shifts matched pattern observed for C13 (*Z*) and (*E*)-methyl enoates of bryostatin 1/SUW201 (*Science* **2017**, *358*, 218-223).

Characterization data for SUW217 – SUW220, SUW229, and SUW230 provided below.

Characterization data for SUW217

TLC R_f = 0.31 (60% EtOAc/pentane, purple spot in p-anisaldehyde)

$[\alpha]^{25.0}_D$ = 24.0° (c = 0.08, CH_2Cl_2)

IR (thin film) 3463, 3341, 2926, 2854, 1653, 1436, 1365, 1247, 1003, 860 cm^{-1}

^{1}H NMR (600 MHz, $CDCl_3$) δ 7.29 – 7.24 (m, 1H) 6.19 – 6.15 (m, 2H), 6.01 (d, *J* = 2.1 Hz, 1H), 5.81 (d, *J* = 3.8 Hz, 1H), 5.79 (d, *J* = 4.3 Hz, 1H), 5.67 (s, 1H), 5.31 (dd, *J* = 15.7, 8.4 Hz, 1H), 5.24 – 5.18 (m, 3H), 5.15 (dd, *J* = 11.9, 4.7 Hz, 1H), 4.22 (t, *J* = 11.6 Hz, 1H), 4.16 (q, *J* = 7.1 Hz, 3H), 4.08 (app t, *J* = 9.7 Hz, 1H), 4.02 (app t, *J* = 11.3 Hz, 1H), 3.84 – 3.74 (m, 2H), 3.71– 3.64 (m, 1H), 3.67 (s, 3H), 2.49 (d, *J* = 10.8 Hz, 1H), 2.43 (t, *J* = 11.8 Hz, 1H), 2.34 (br s, 1H), 2.21 (t, *J* = 12.4 Hz, 1H), 2.18 – 2.12 (m, 2H), 2.12 – 2.06 (m, 2H), 2.05 (s, 3H), 2.05 – 1.92 (m, 1H), 1.91 (t, *J* = 12.8 Hz, 1H), 1.83 (t, *J* = 12.3 Hz, 1H), 1.79 – 1.74 (m, 1H), 1.67 (d, *J* = 15.1 Hz, 1H), 1.60 (app d, *J* = 15.1 Hz, 1H), 1.51 – 1.42 (m, 3H), 1.28 (t, *J* = 7.1 Hz, 4H), 1.27 – 1.21 (m, 12H), 1.15 (s, 3H), 1.01 (s, 6H), 0.95 (s, 3H), 0.92 (t, *J* = 7.4 Hz, 3H).

^{13}C NMR (126 MHz, $CDCl_3$) δ 172.31, 170.97, 167.16, 166.43, 165.74, 156.15, 152.11, 146.52, 145.67, 139.37, 129.56, 128.51, 119.72, 118.74, 115.08, 101.94, 99.14, 79.28, 74.18, 73.82, 72.86, 71.60, 70.31, 68.60, 65.97, 64.83, 59.93, 51.23, 45.05, 44.25, 42.53, 42.12, 41.09, 39.98, 36.43, 36.00, 35.21, 33.46, 32.08, 31.43, 24.73, 22.02, 21.20, 19.97, 16.96, 14.44, 13.85, 1.18.

HRMS calculated for $C_{48}H_{70}NaO_{17}$ $[M+Na]^+$: 941.4505; found 941.4478 (TOF ESI+)

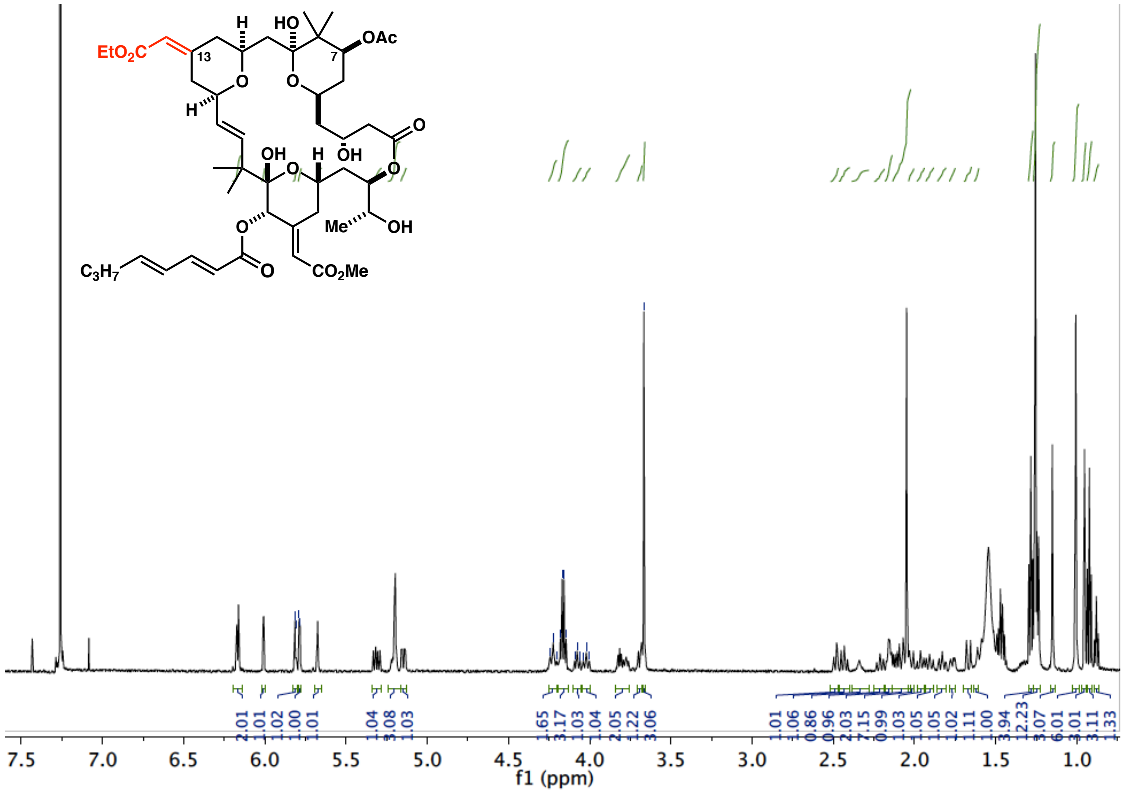

Supplementary Figure 9. ^{1}H NMR (600 MHz, $CDCl_3$) of SUW217.

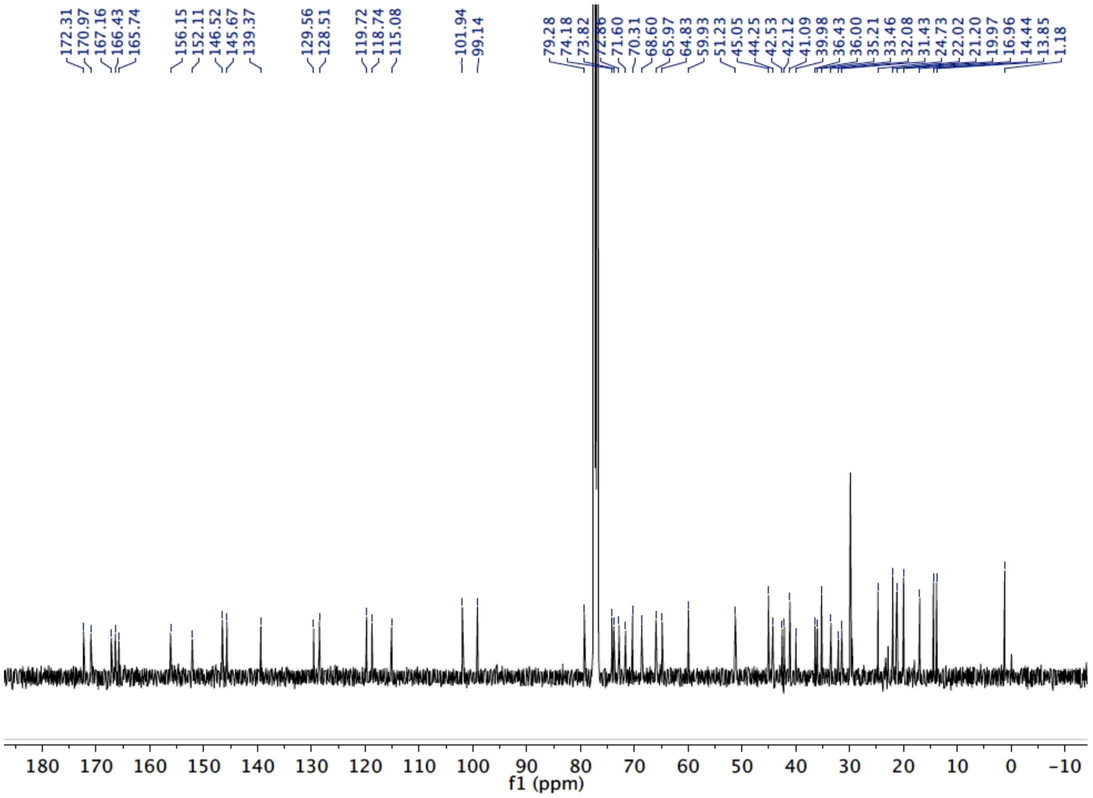

Supplementary Figure 10. ^{13}C NMR (125 MHz, $CDCl_3$) of SUW217.

Characterization data for SUW218

TLC R_f = 0.31 (60% EtOAc/pentane, purple spot in *p*-anisaldehyde)

$[\alpha]^{24.7}_D$ = 6.66 (c = 0.13, CH_2Cl_2)

IR (thin film) 3463, 3343, 2927, 2854, 1734, 1716, 1644, 1435, 1241, 1146, 1003 cm^{-1}

^{1}H NMR (600 MHz, $CDCl_3$) δ 7.30 – 7.23 (dd obscured by chloroform peak, 1H), 6.19 – 6.13 (m, 2H), 6.01 (d, *J* = 1.9 Hz, 1H), 5.82 (d, *J* = 9.8 Hz, 1H), 5.79 (d, *J* = 9.2 Hz, 1H), 5.70 (s, 1H), 5.30 (dd, *J* = 15.7, 8.5 Hz, 1H), 5.23 (s, 1H), 5.23 – 5.20 (m, 1H), 5.20 (s, 1H), 5.15 (dd, *J* = 11.8, 4.8 Hz, 1H), 4.25 (m, 2H), 4.19 – 4.10 (m, 4H), 4.03 (t, *J* = 11.2 Hz, 1H), 3.82 (p, *J* = 6.3 Hz, 1H), 3.76 – 3.67 (m, 3H), 3.67 (s, 3H), 2.49 (dd, *J* = 12.4, 2.8 Hz, 1H), 2.45 (q, *J* = 12.5, 11.4 Hz, 1H), 2.19 – 2.06 (m, 5H), 2.04 (s, 3H), 2.03 – 1.91 (m, 3H), 1.87 – 1.79 (m, 1H), 1.79 – 1.75 (m, 1H), 1.76 (d, *J* = 15.2 Hz, 1H), 1.51 – 1.41 (m, 3H), 1.28 (t, *J* = 7.1 Hz, 3H), 1.26 (s, 3H), 1.23 (d, *J* = 6.5 Hz, 3H), 1.14 (s, 3H), 1.01 (s, 3H), 1.00 (s, 3H), 0.95 (s, 3H), 0.92 (t, *J* = 7.4 Hz, 3H), 0.88 (t, *J* = 7.0 Hz, 1H).

^{13}C NMR (126 MHz, $CDCl_3$) δ 172.39, 170.93, 167.14, 166.79, 165.70, 156.47, 152.00, 146.52, 145.70, 139.70, 129.22, 128.50, 119.79, 118.73, 114.96, 101.88, 99.13, 79.91, 74.15, 73.70, 72.95, 71.50, 70.24, 68.72, 65.91, 64.84, 59.97, 51.24, 45.03, 43.14, 42.56, 42.20, 41.16, 40.04, 37.58, 35.90, 35.21, 33.51, 31.42, 24.73, 22.02, 21.33, 21.20, 19.91, 19.83, 16.94, 14.43, 13.85.

HRMS calculated for $C_{48}H_{70}NaO_{17}$ $[M+Na]^+$: 941.4505; found 941.4485 (TOF ESI+)

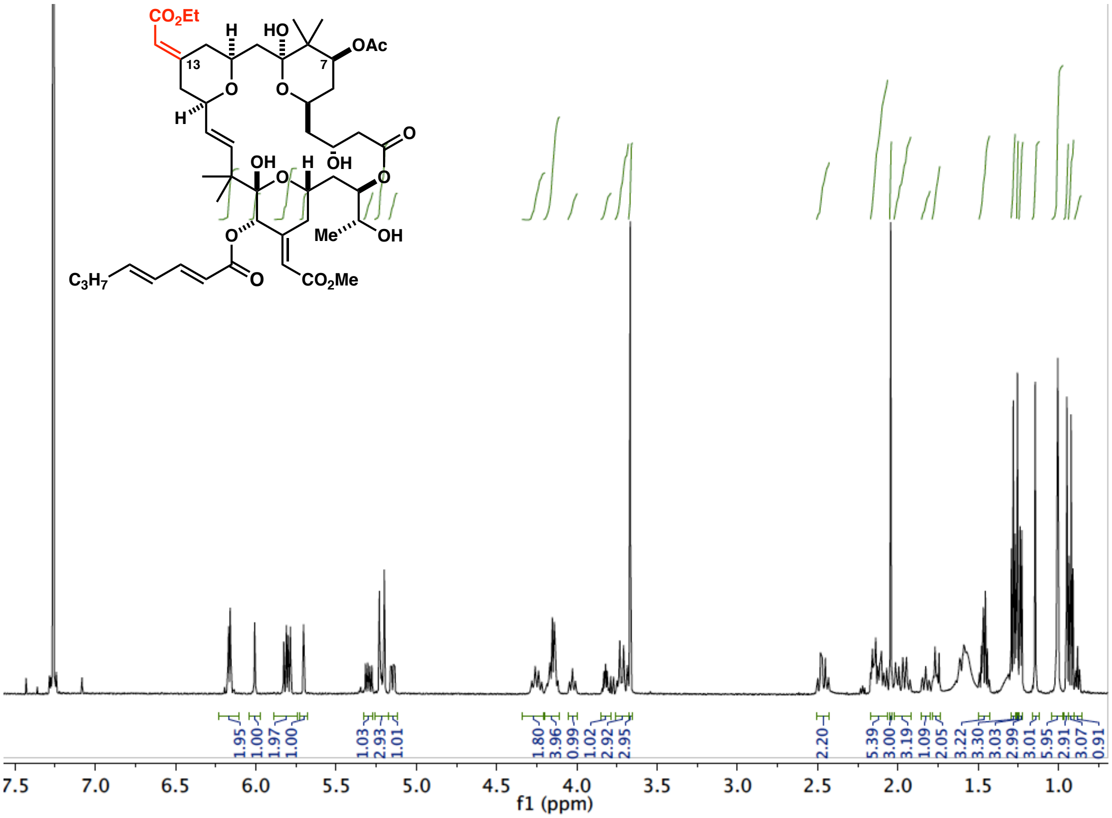

Supplementary Figure 11. ^{1}H NMR (600 MHz, $CDCl_3$) of SUW218.

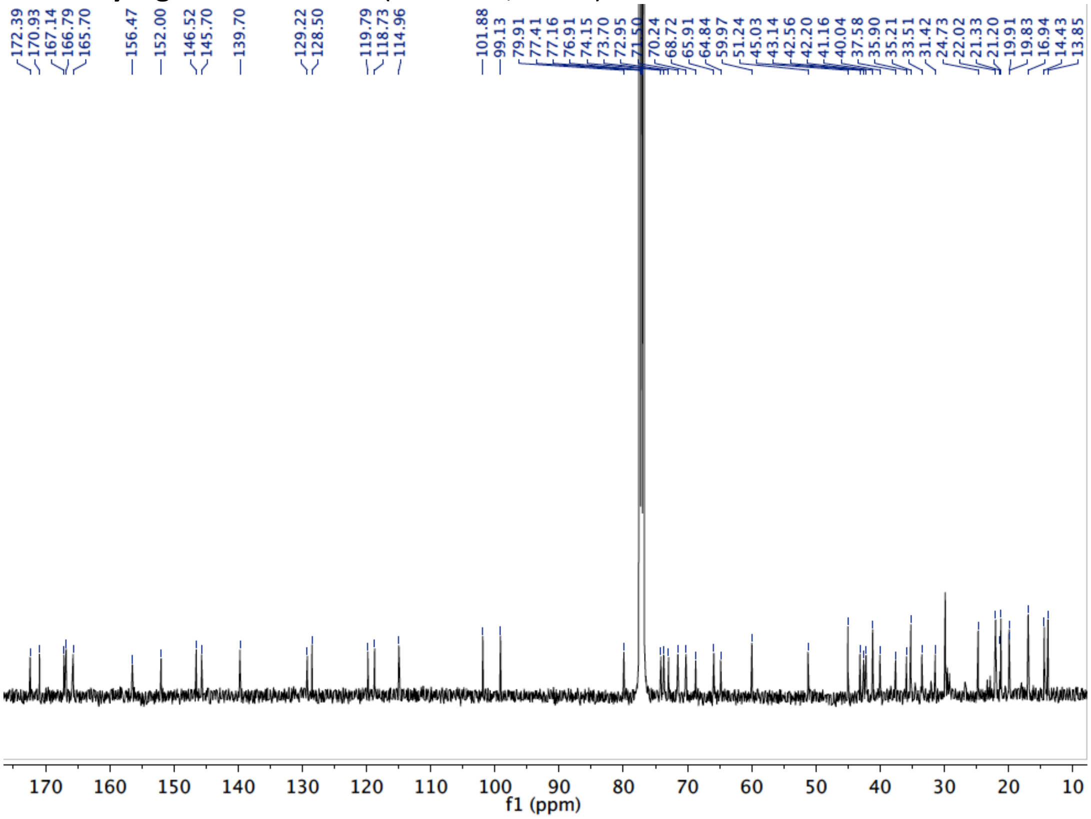

Supplementary Figure 12. ^{13}C NMR (125 MHz, $CDCl_3$) of SUW218.

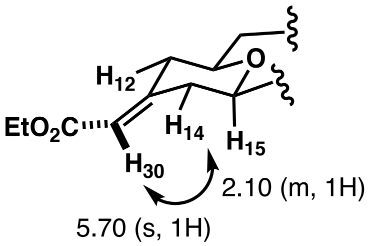

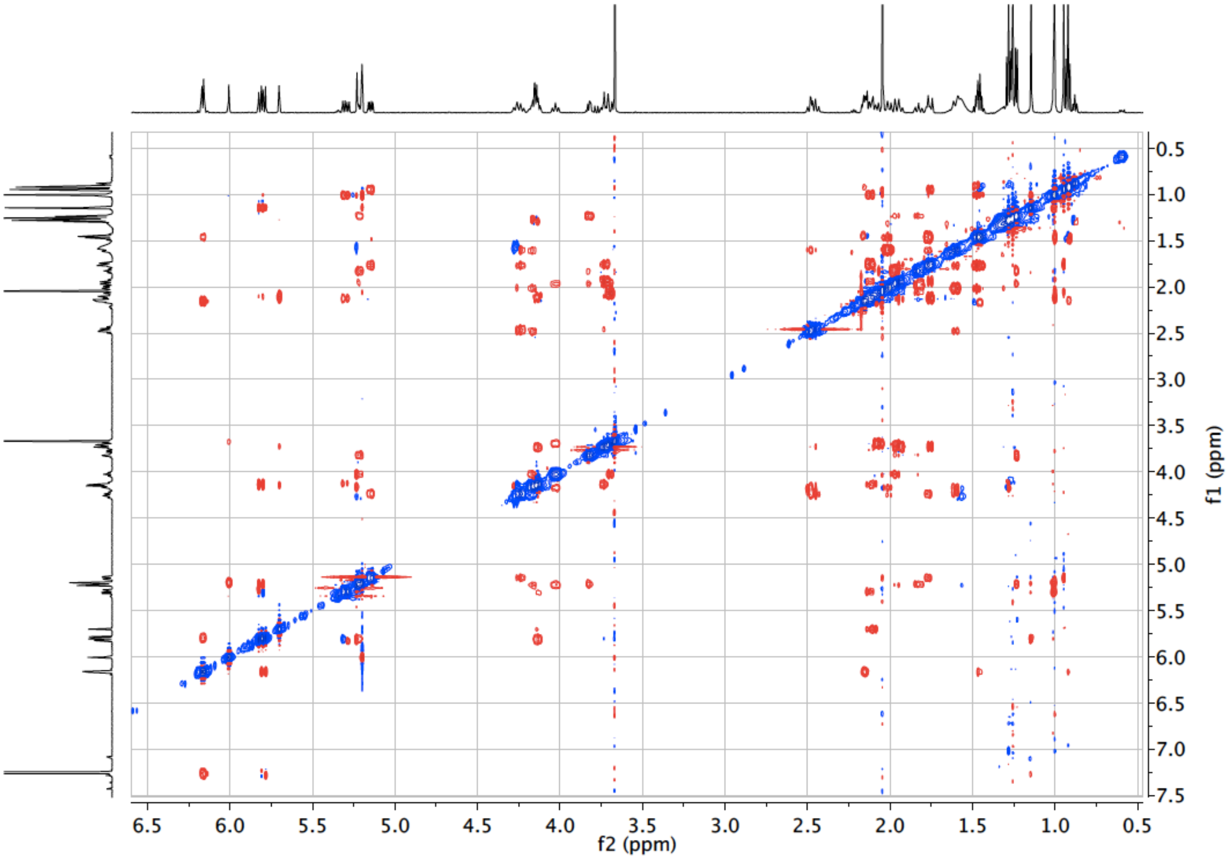

Supplementary Figure 13. Observed ROE between H_{30} and H_{14}(eq) confirms C13 enoate geometry. ROESY NMR (600 MHz, $CDCl_3$) of SUW218. ^1H peaks assigned via COSY NMR (not shown).

Characterization data for SUW219

TLC: R_f = 0.32 (50 % EtOAc/pentane, UV active, purple spot by *p*-anisaldehyde stain)

$[\alpha]^{23.2}_D$ = + 5.9 ° (c = 0.1, CH_2Cl_2).

IR (thin film): 3467, 3314, 2927, 1717, 1654, 1647, 1458, 1437, 1364, 1287, 1246, 1161, 1079, 1058, 1027, 1004, and 860 cm^{-1}.

^{1}H-NMR (600 MHz, $CDCl_3$)**:** δ 7.37 – 7.31 (m, 5H, phenyl), 7.29 – 7.25 (m, 1H, obscured by residual chloroform peak, $C_{41}H$), 6.18 – 6.16 (m, 2H, $C_{42}H$, and $C_{43}H$), 6.01 (d, 1H, J = 1.8 Hz, $C_{34}H$), 5.81 (d, 1H, J = 15.2 Hz, $C_{40}H$), 5.80 (d, 1H, J = 15.8 Hz, $C_{17}H$), 5.74 (t, 1H, J = 1.6 Hz, $C_{30}H$), 5.31 (dd, 1H, J = 15.8, 8.4 Hz, $C_{16}H$), 5.23 – 5.11 (m, 6H), 4.25 – 4.15 (m, 3H, C_3-OH, C_3H, C_5H), 4.09 (ddd, 1H, J = 11.0, 8.5, 2.2 Hz, $C_{15}H$), 4.02 (tt, 1H, J = 11.5, 2.2 Hz, $C_{22}H$), 3.83 – 3.74 (m, 2H), 3.71 (s, 1H), 3.68 (s, 1H), 3.67 (s, 3H, CO_2Me), 2.50 – 2.41 (m, 2H), 2.36 (s, 1H), 2.13 (t, 1H, J = 12.4 Hz), 2.18 – 2.14 (m, 2H), 2.12 (dd, 1 H, J = 7.8, 15.2 Hz), 2.07 (bs, 1H), 2.05 (s, 3H, C_7OAc), 2.03 – 1.97 (m, 2H), 1.94 – 1.89 (m, 2H), 1.86 – 1.81 (m, 1H), 1.76 (ddd, 1H, J = 2.7, 4.5, 12.5 Hz), 1.59 (dt, 1H, J = 3.1, 14.9 Hz), 1.51 – 1.43 (m, 4H), 1.24 (d, 3H, J = 6.5 Hz, $C_{27}H$), 1.15 (s, 3H), 1.01 (s, 6H), 0.95 (s, 3H), and 0.93 (t, 3H, J = 7.4 Hz, $C_{46}H$) ppm.

^{13}C-NMR (125 MHz, $CDCl_3$): δ 172.41, 171.00, 167.25, 166.23, 165.84, 156.96, 152.20, 146.63, 145.78, 139.54, 136.35, 129.60, 128.81, 128.62, 128.41, 128.40, 128.39, 128.17, 119.83, 118.84, 114.93, 102.03, 99.24, 79.36, 74.23, 73.94, 72.90, 71.65, 70.41, 68.71, 66.11, 65.98, 64.93, 51.33, 45.16, 44.35, 42.65, 42.24, 41.18, 40.06, 36.58, 36.09, 35.32, 33.55, 31.52, 24.83, 22.12, 21.42, 21.29, 20.09, 20.03, 17.06, and 13.96 ppm.

HRMS: calculated for $C_{53}H_{72}O_{17}Na$ [M+Na^+]: 1003.4662; found 1003.4649.

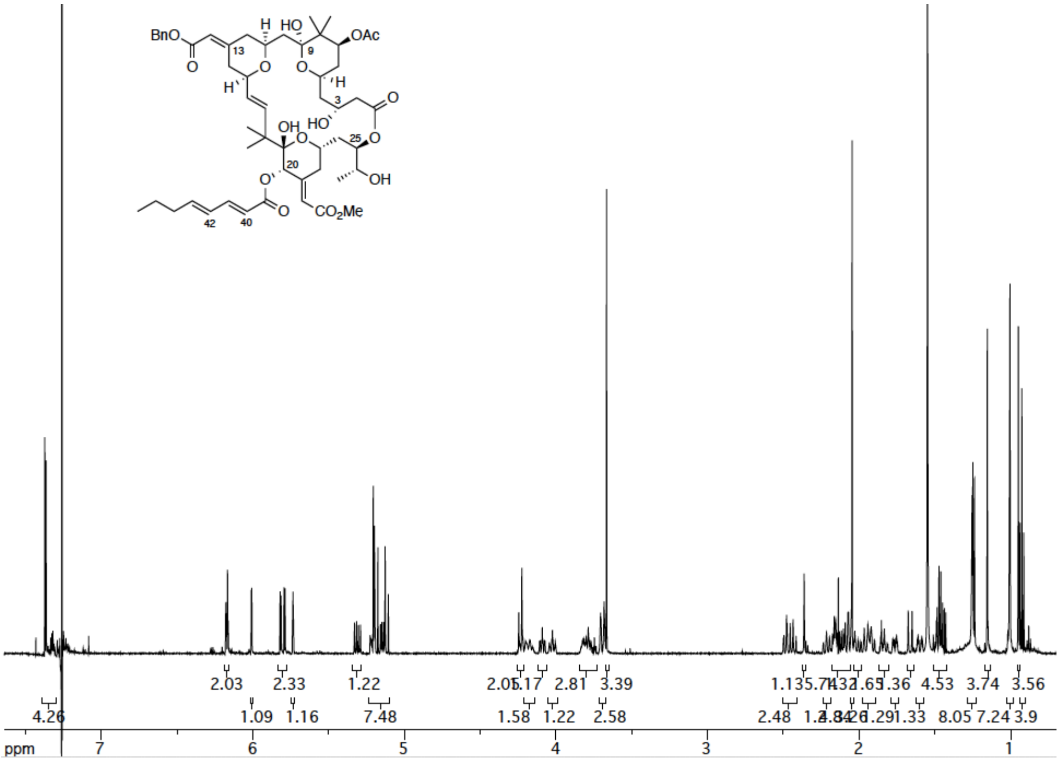

Figure 14. ^{1}H NMR (600 MHz, $CDCl_3$) of SUW219.

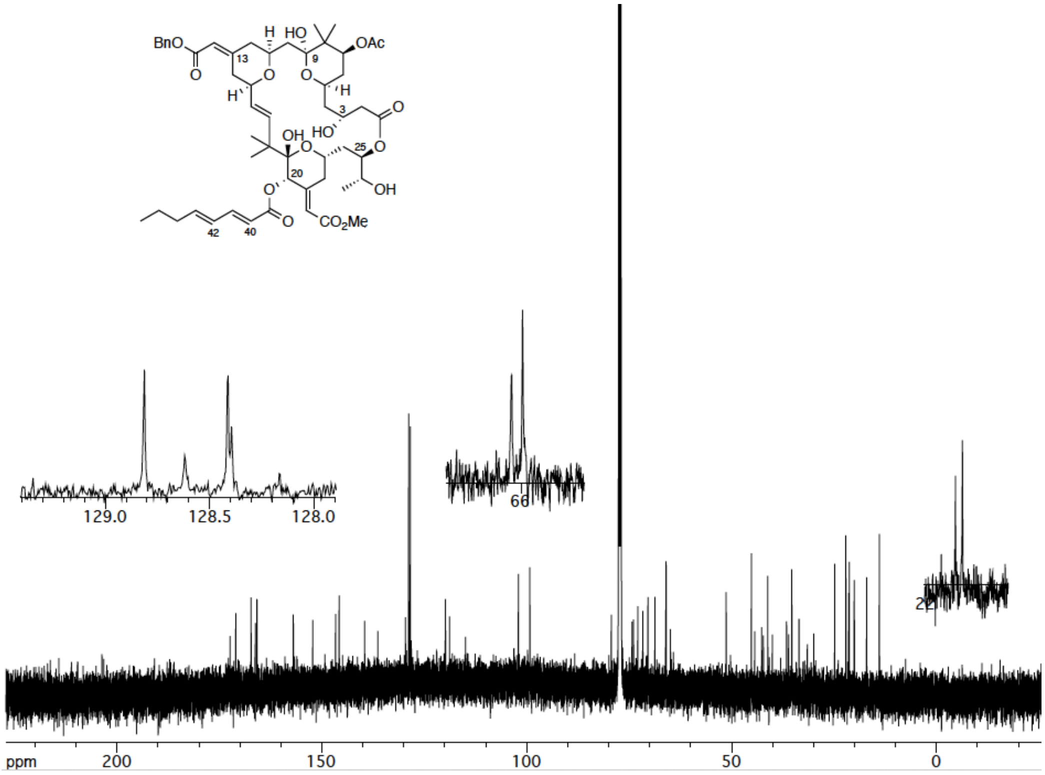

Supplementary Figure 15. ^{13}C NMR (125 MHz, $CDCl_3$) of SUW219.

Characterization data for SUW220

TLC: R_f = 0.38 (50 % EtOAc/pentane, UV active, purple spot by *p*-anisaldehyde stain)

$[\alpha]^{23.2}_D$ = - 6.8 ° (c = 0.1, CH_2Cl_2).

IR (thin film): 3466, 3352, 2961, 2928, 1715, 1642, 1455, 1435, 1408, 1366, 1283, 1241, 1260, 1141, 1098, 1078, 1056, 1027, and 1004 cm^{-1}.

^{1}H-NMR (600 MHz, $CDCl_3$): δ 7.37 – 7.30 (m, 5H, Ph), 7.28 – 7.24 (m, 1H, obscured by the chloroform peak, $C_{41}H$), 6.17 – 6.16 (m, 2H, $C_{42}H$, $C_{43}H$), 6.01 (s, 1H, $C_{34}H$), 5.81 (d, 1H, J = 15.7 Hz), 5.79 (d, 1H, J = 15.3 Hz), 5.77 (s, 1H, $C_{30}H$), 5.30 (dd, 1H, J = 8.1, 15.7 Hz, $C_{16}H$), 5.24 – 5.11 (m, 6H), 4.26 (d, 1H, J = 12.0 Hz, C_3-OH), 4.24 (tt, 1H, J = 3.0, 12.0 Hz, C_5H), 4.22 (m, 2H), 4.19 – 4.12 (m, 2H), 4.02 (t, 1H, J = 11.2 Hz, $C_{23}H$), 3.82 (app. hextet, 1H, J = 6.0, $C_{26}H$), 3.76 – 3.68 (m, 2H), 3.67 (s, 3H, CO_2Me), 2.49 – 2.44 (m, 3H), 2.17 – 2.06 (m, 6H), 2.05 (s, 3H, C_7-OAc), 2.03 – 1.93 (m, 2H), 1.91 – 1.73 (m, 4H), 1.62 – 1.59 (m, 2H), 1.31 – 1.25 (m, 2H), 1.20 (d, J = 6.4 Hz, 3H, $C_{27}H$), 1.11 (s, 3H), 0.98 (bs, 6H), 0.92 (s, 3H), and 0.89 (t, 3H, J = 7.4 Hz) ppm.

^{13}C-NMR (125 MHz, $CDCl_3$): δ 172.51, 171.03, 167.24, 166.58, 165.80, 157.39, 152.09, 146.63, 145.81, 139.88, 136.35, 129.26, 128.82, 128.60, 128.42, 128.39, 119.89, 118.83, 114.73, 101.99, 99.23, 80.00, 77.47, 74.25, 73.80, 73.04, 71.58, 70.34, 68.82, 66.03, 65.98, 64.95, 51.35, 45.14, 43.31, 42.65, 42.29, 41.27, 40.13, 37.77, 35.99, 33.60, 32.19, 31.51, 24.82, 22.60, 22.12, 21.44, 21.31, 20.02, 19.93, 17.05, and 13.95 ppm.

HRMS: calculated for $C_{53}H_{72}O_{17}Na^+$ [$M+Na^+$]: 1003.4662, found 1003.4650.

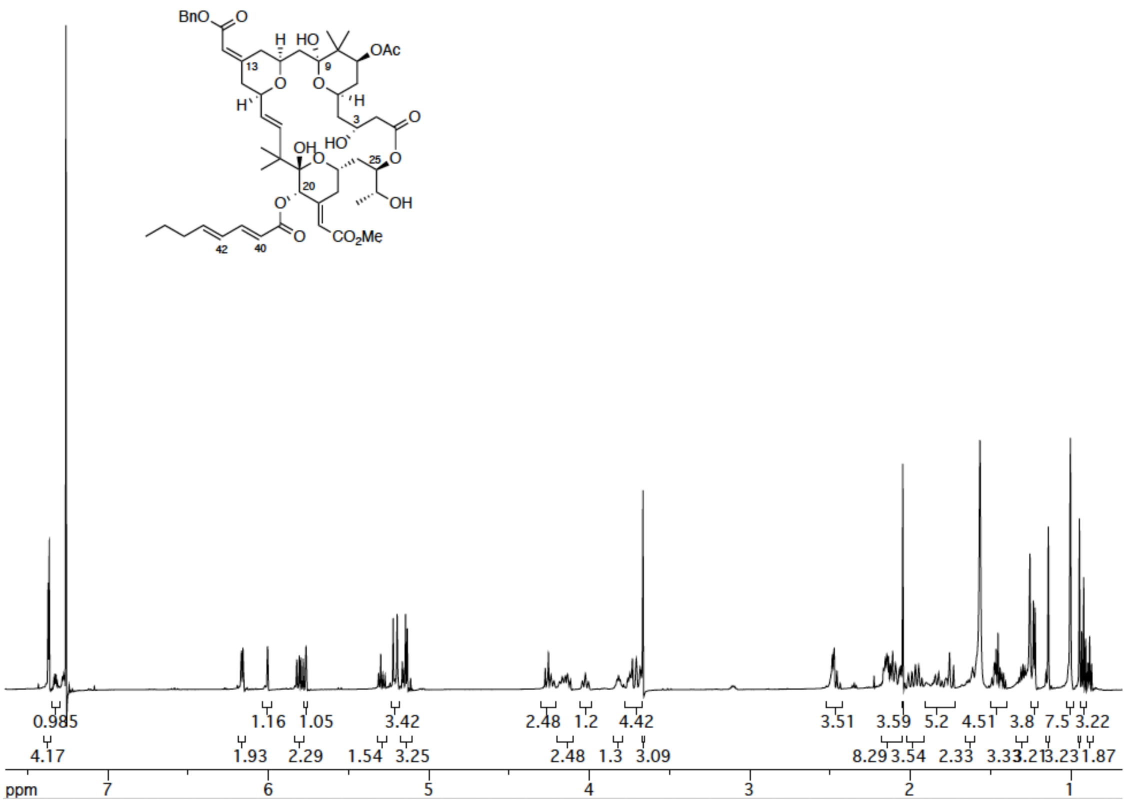

Supplementary Figure 16. ^{1}H NMR (600 MHz, $CDCl_3$) of SUW220.

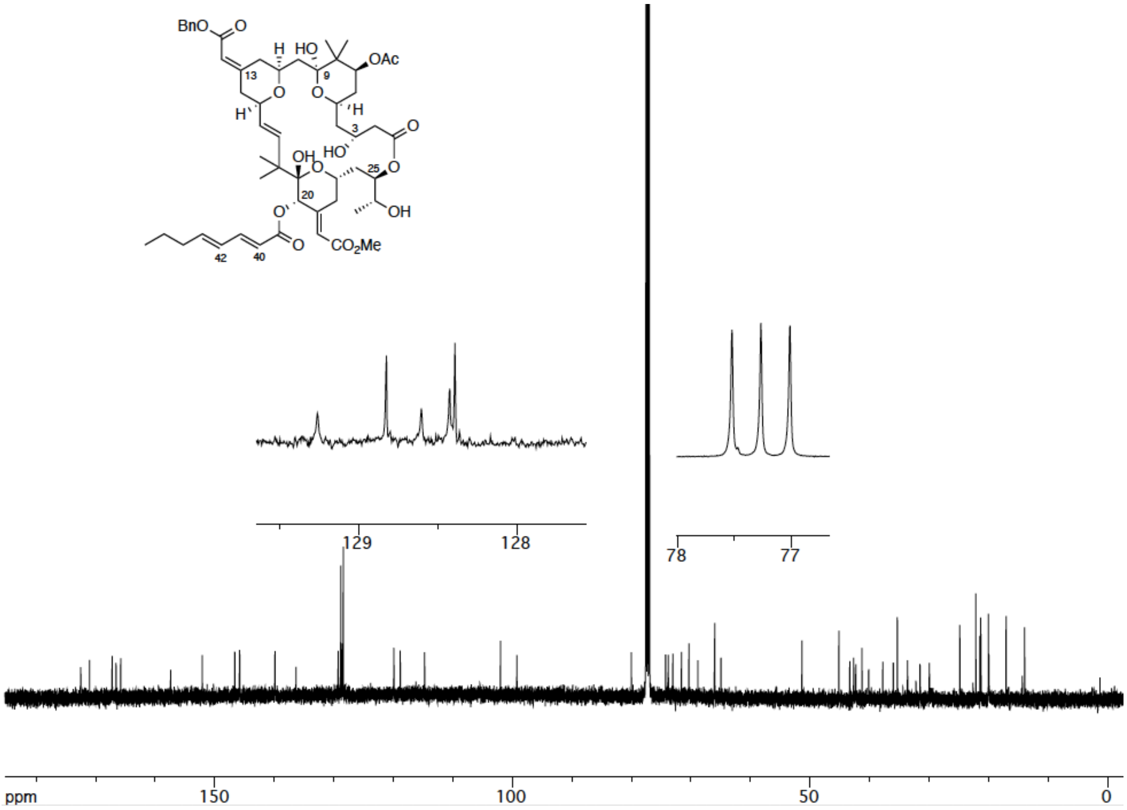

Supplementary Figure 17. ^{13}C NMR (125 MHz, $CDCl_3$) of SUW220.

Characterization data for SUW229

TLC R_f = 0.38 (60% EtOAc/hexanes, purple spot in *p*-anisaldehyde)

$[\alpha]^{24.7}_D$ = 7.66 (c = 0.07, CH_2Cl_2)

IR (thin film) 3460, 3355, 3312, 2959, 2919, 2580, 1717, 1662, 1634, 1261, 1157, 1023 cm^{-1}

^{1}H NMR (600 MHz, $CDCl_3$) δ 7.30 – 7.23 (dd obscured by chloroform peak, 1H), 6.19 – 6.15 (m, 2H), 6.01 (s, 1H), 5.95 (m, 1H), 5.81 (s, 1H), 5.79 (s, 1H), 5.72 (s, 1H, C30 H), 5.37 – 5.28 (m, 2H), 5.27 – 5.17 (m, 3H), 5.20 (s, 1H) 5.14 (d, *J* = 12.1 Hz, 1H), 4.62 (appf s, 2H), 4.26 – 4.13 (m, 3H), 4.08 (app. t, *J* = 10.0 Hz, 1H), 4.02 (app. t, *J* = 12.8 Hz, 1H), 3.82 (m, 1H), 3.77 (m, 1H), 3.70 (m, 1H), 3.69 – 3.66 (m, 1H) 3.67 (s, 3H), 2.49 (app d, *J* = 12.5 Hz), 2.43 (app t, *J* = 11.5 Hz, 1H), 2.05 (s, 3H),z 1.15 (s, 3H), 1.01 (s, 6H), 0.96 (s, 3H), 0.92 (t, *J* = 7.4 Hz, 3H).

^{13}C NMR (126 MHz, $CDCl_3$) δ 172.39, 170.93, 167.11, 165.99, 165.73, 152.09, 146.52, 139.43, 132.49, 129.50, 128.51, 128.42, 119.73, 118.74, 118.22, 114.73, 101.94, 99.14, 79.29, 74.17, 73.87, 72.83, 71.58, 70.29, 68.59, 65.99, 64.82, 64.70, 51.31, 51.23, 45.06, 44.26, 42.52, 42.13, 41.08, 39.95, 36.47, 35.22, 32.08, 29.85, 24.72, 22.85, 22.02, 21.33, 21.19, 19.96, 16.96, 14.29, 13.85.

HRMS calculated for $C_{49}H_{70}O_{17}$ [M + Na^+]: 953.4505; found 953.4475 (TOF ESI+).

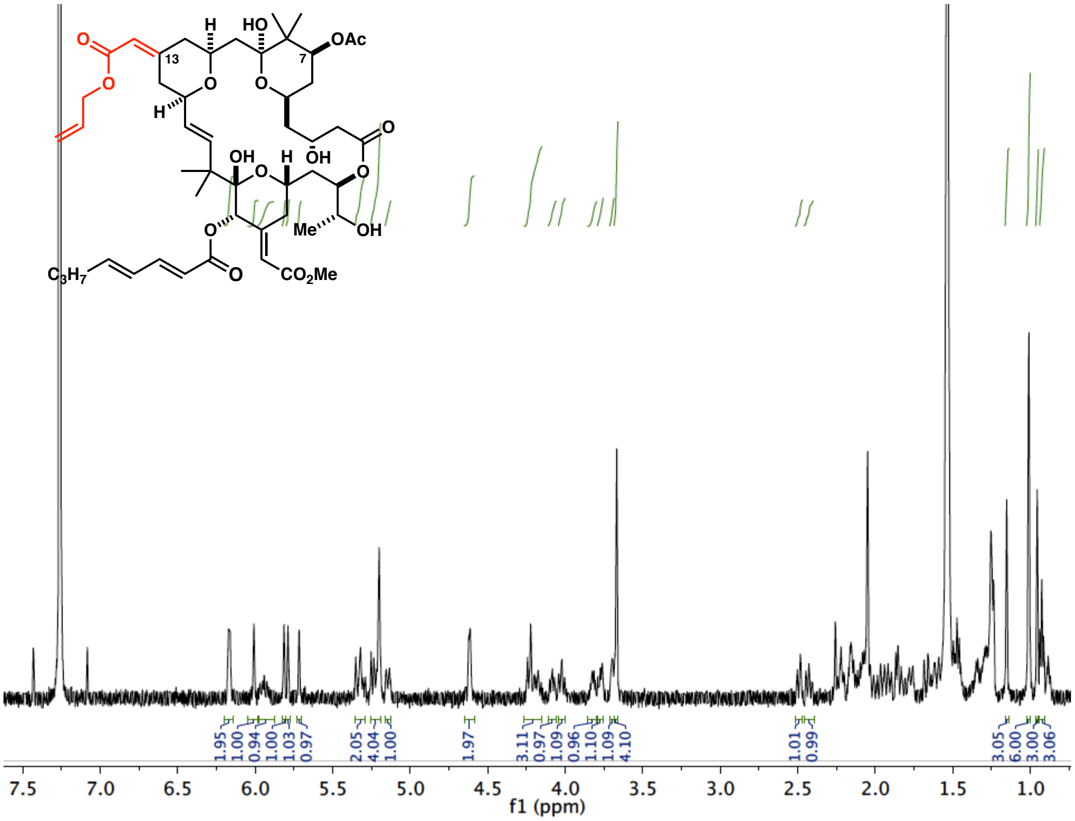

Supplementary Figure 18. ^{1}H NMR (600 MHz, $CDCl_3$) of SUW229.

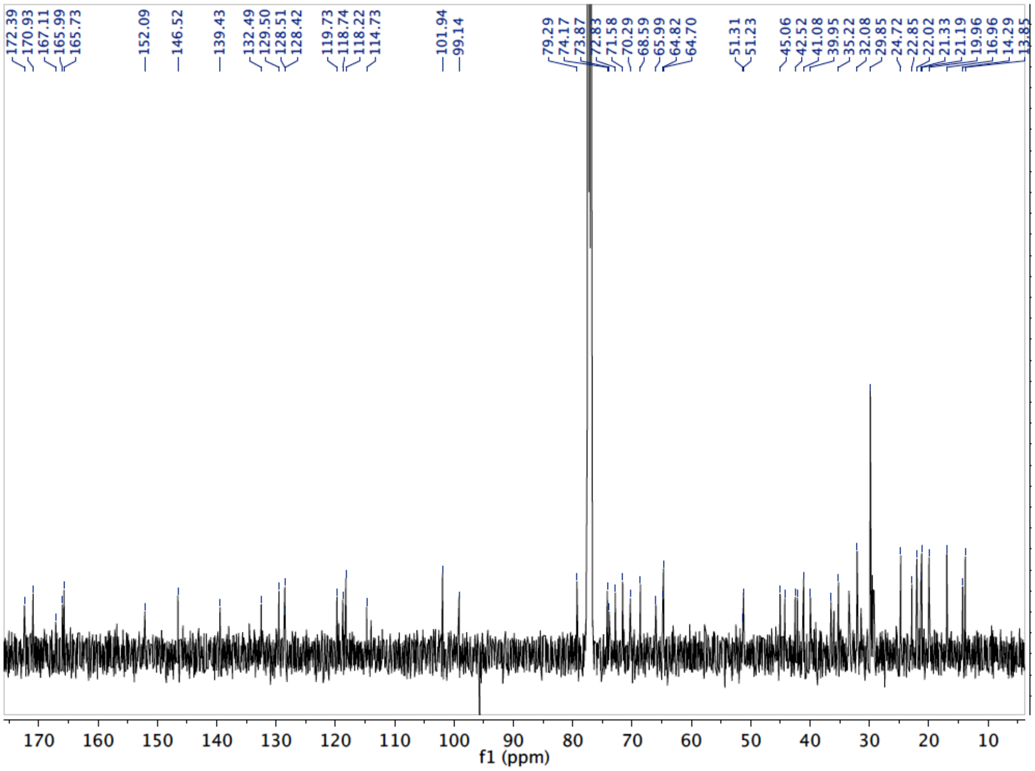

Supplementary Figure 19. ^{13}C NMR (126 MHz, $CDCl_3$) of SUW229.

Characterization data for SUW230

TLC R_f = 0.46 (60% EtOAc/hexanes, purple spot in *p*-anisaldehyde)

$[\alpha]^{25.0}_{D}$ = -30.10 (c = 0.07, CH_2Cl_2)

IR (thin film) 3463, 3358, 2962, 2918, 2850, 1719, 1653, 1559, 1540, 1261, 1080, 1026 cm^{-1}

^{1}H NMR (600 MHz, $CDCl_3$) δ 7.30 – 7.23 (dd obscured by chloroform peak, 1H), 6.18 – 6.14 (m, 2H), 6.01 (s, 1H), 5.98 – 5.88 (m, 1H), 5.81 (app t, *J* = 14.0 Hz, 2H), 5.75 (s, 1H, C30 H), 5.33 (d, *J* = 17.4 Hz, 1H), 5.31 – 5.18 (m, 3H), 5.22 (s, 1H), 5.20 (s, 1H), 5.15 (d, *J* = 13.4 Hz, 1H), 4.60 (app s, 2H), 4.29 – 4.21 (m, 2H), 4.21 – 4.12 (m, 2H), 4.03 (t, *J* = 10.7 Hz, 1H), 3.82 (m, 1H), 3.76 – 3.68 (m, 3H), 3.67 (s, 3H), 2.52 – 2.41 (m, 2H), 2.33 (s, 1H), 2.04 (s, 3H), 1.15 (s, 3H), 1.01 (s, 6H), 0.95 (s, 3H), 0.92 (t, *J* = 7.8 Hz, 3H).

^{13}C NMR (126 MHz, $CDCl_3$) δ 172.41, 170.91, 167.14, 166.36, 165.70, 157.20, 151.99, 146.52, 145.70, 139.76, 132.45, 129.17, 128.50, 119.79, 118.73, 118.26, 114.57, 101.88, 99.13, 74.14, 73.71, 72.93, 71.50, 70.24, 68.72, 65.93, 64.84, 64.73, 51.24, 45.03, 43.18, 42.55, 42.18, 41.17, 40.02, 37.62, 35.88, 35.21, 33.49, 31.41, 29.86, 24.72, 22.02, 21.33, 21.19, 19.91, 19.83, 16.94, 13.85.

HRMS calculated for $C_{49}H_{70}O_{17}$ [M + Na^+]: 953.4505; found 953.4479 (TOF ESI+).

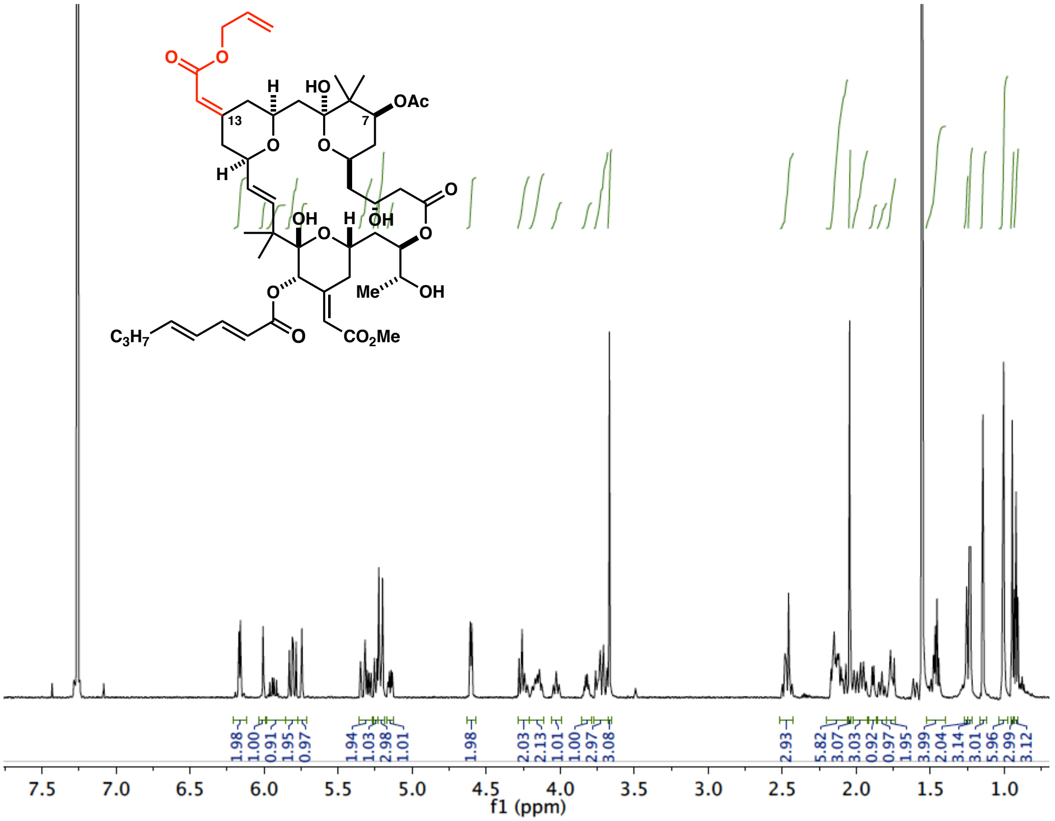

Supplementary Figure 20. ^{1}H NMR (600 MHz, $CDCl_3$) of SUW230.

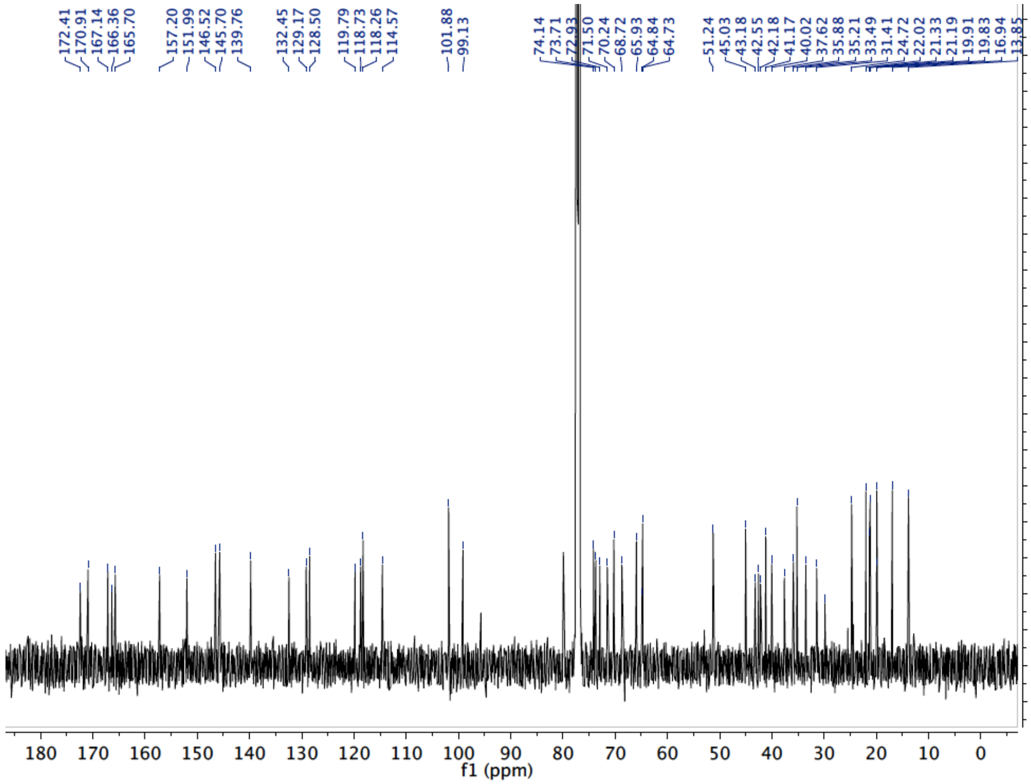

Supplementary Figure 21. ^{13}C NMR (126 MHz, $CDCl_3$) of SUW230.

SUW204

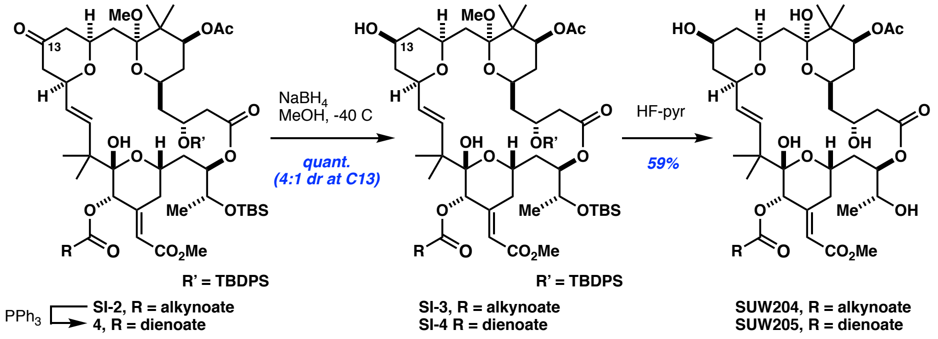

Reduction: To a cooled (-20 °C) solution of **SI-2** (14 mg, 0.012 mmol, 1 equiv) in MeOH (1.15 mL, 0.01M) was added sodium borohydride (0.5 mg, 0.013 mmol, 1.15 equiv). After 2h, TLC analysis indicated full conversion of starting material. The now yellow reaction mixture was quenched at -20 °C by adding saturated aqueous NH_4Cl (2 mL). The layers were separated, and the aqueous layer was extracted with 50% EtOAc/Hex (5x3 mL). The combined organic layers were dried over $NaSO_4$, filtered, and concentrated. Purification was accomplished by flash column chromatography using a 1.7x6 cm silica gel column, eluting with 15-25% EtOAc/Hex, and collecting 4 mL fractions. Frxns #20-26 afforded 11.6 mg (83% yield) of **SI-3** and frxns #27-31 afforded 2 mg (14% yield) of its C13 diastereomer (structure not shown). The relative stereochemistry at C13 was assigned after C13-acetylation to afford **SUW206** and **SUW207**, respectively. **TLC** of **SI-3**: R_f = 0.44 (30% EtOAc/Hex, UV active); **TLC** of C13 diastereomer of **SI-3**: R_f = 0.63 (30% EtOAc/Hex, UV active).

Global deprotection: To a cooled (0 °C) solution of **SI-3** (11.6 mg, 0.01 mmol, 1 equiv) in 3:1 THF/H_2O (1 mL, 0.01M) was added 70% HF-pyridine (300 uL). The reaction mixture was allowed to warm to room temperature. After 48h, TLC analysis indicated incomplete deprotection so additional 70% HF-pyridine (150 uL; total of 450 uL) was added. After an additional 24h (total reaction time of 72h), the reaction mixture was cooled to 0 °C, diluted with 50% EtOAc/Hex (5 mL), and slowly quenched by adding saturated aqueous $NaHCO_3$ dropwise until bubbling ceased. The layers were separated, and the aqueous layer was extracted with 80% EtOAc/Hex (5x5 mL). The combined organic layers were dried over $NaSO_4$, filtered, and concentrated. Purification was accomplished by silica gel flash column chromatography (25% EtOAc/Hex to remove silanol, then 50-100% EtOAc/Hex), followed by RP-HPLC (70-100% MeCN/H_2O) affording **SUW204** (4.8 mg, 59% yield). The same reduction/deprotection sequence was repeated with dienoate **4** to afford **SI-4** and ultimately **SUW205** (data not shown)

Characterization data for SUW204

TLC R_f = 0.27 (100% EtOAc, not UV active, stains in CAM)

$[\alpha]^{24}_D$ = 6.8° (*c* = 0.15, CH_2Cl_2)

IR (thin film): 3454 (bs), 2927, 2855, 1715, 1366, 1286, 1160, 1058 cm^{-1}

^{1}H-NMR (600 MHz, C_6D_6) δ 6.43 (s, 1H, $C_{34}H$), 6.19 (d, *J* = 15.8 Hz, 1H, $C_{17}H$), 5.75 (s, 1H, $C_{20}H$), 5.50 (dd, *J* = 15.8, 8.3 Hz, 1H, $C_{16}H$), 5.39 – 5.30 (m, 2H, $C_{25}H$, C_7H), 4.42 (app. t, *J* = 11.8 Hz, 1H, $C_{23}H$), 4.30 (app. t, *J* = 10.4 Hz, 1H, $C_{15}H$), 4.26 (app. d, *J* = 13.9 Hz, 1H, $C_{22}H_{eq}$), 4.11 – 4.04 (m, 1H, C_3H), 3.86 (app. t, *J* = 12.1 Hz, 1H, C_5H), 3.75 – 3.68 (m, 1H, $C_{13}H$), 3.68 – 3.61 (m, 2H, $C_{11}H$, $C_{26}H$), 3.21 (s, 3H), 2.47 – 2.37 (m, 2H, C_2H_a, $C_{22}H_a$), 2.18 (d, *J* = 12.2 Hz, 1H, C_2H_b), 2.07 (dd, *J* = 15.0, 6.9 Hz, 1H, $C_{10}H_a$), 1.69 (s, 3H), 1.66 (s, 3H), 1.03 (d, *J* = 6.3 Hz, 3H, $C_{27}H_3$), 0.90 (s, 3H), 0.86 (s, 3H), 0.71 (t, *J* = 6.4 Hz, 3H, $C_{46}H_3$)

^{13}C-NMR (125 MHz, C_6D_6) δ 173.1, 170.2, 166.6, 152.5, 151.7, 139.0, 130.2, 121.1, 102.1, 99.7, 91.0, 77.8, 76.2, 74.5, 74.0, 73.0, 70.4, 69.6, 68.7, 67.5, 65.7, 65.4, 50.7, 45.4, 43.6, 42.5, 42.4, 42.4, 41.2, 39.8, 36.0, 33.6, 32.0, 31.8, 31.1, 27.2, 25.4, 22.3, 21.2, 20.7, 19.9, 18.6, 16.9, 14.0

HRMS calculated for $C_{44}H_{66}NaO_{16}$ $[M+Na]^+$: 873.4243; found 873.4215 (TOF ESI+)

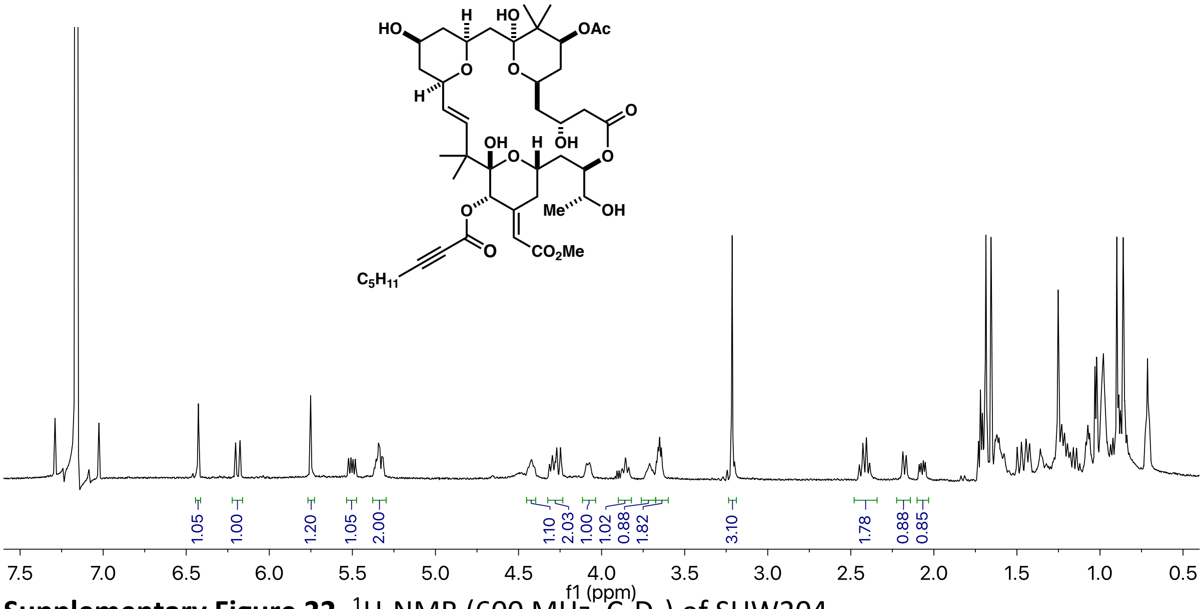

Supplementary Figure 22. ^{1}H-NMR (600 MHz, C_6D_6) of SUW204.

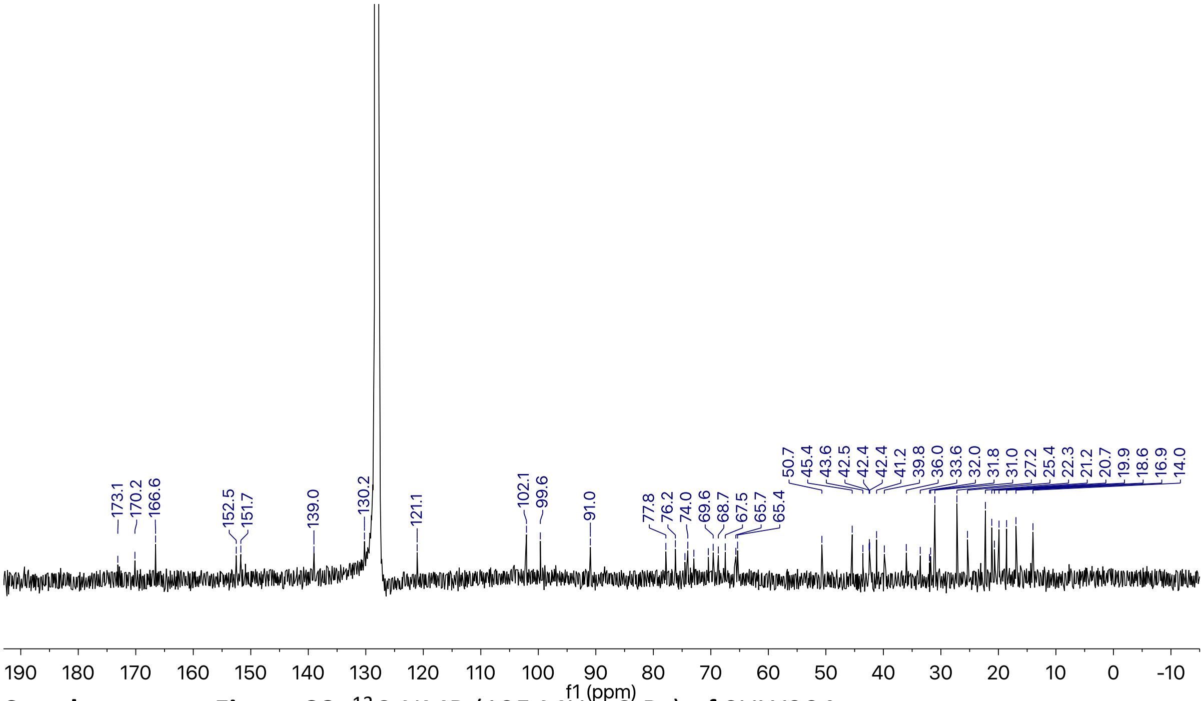

Supplementary Figure 23. ^{13}C-NMR (125 MHz, C_6D_6) of SUW204.

SUW206 and SUW207

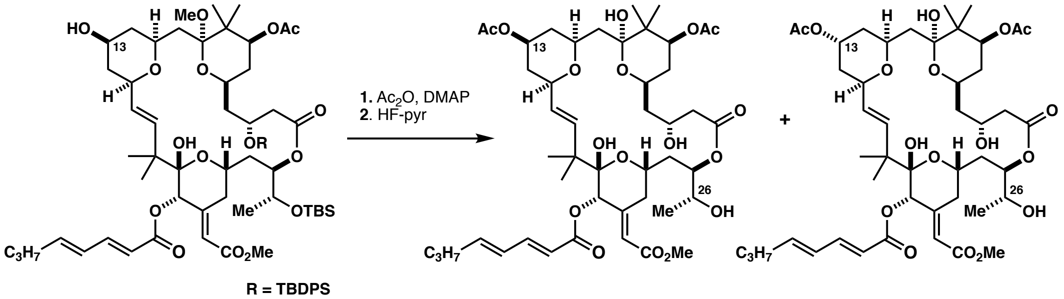

SI-4 (4:1 dr at C13) SUW206 SUW207

Acylation: To a solution of alcohol **SI-4** (19 mg, ~4:1 dr at C13, 0.016 mmol, 1 equiv) in CH_2Cl_2 (156 uL, 0.1M) was sequentially added DMAP (1 crystal) and acetic anhydride (2 drops). After 3h, TLC analysis indicated complete conversion of starting material. The reaction mixture was directly flashed via silica gel flash column chromatography (20-30% EtOAc/Hex) affording the C13-OAc (quant., ~4:1 dr at C13).

Global deprotection: To a cooled (0 °C) solution of this ester (19 mg, ~4:1 dr at C13, 0.016 mmol, 1 equiv) in 1:1 THF / pyridine was added 70% HF-pyridine (~0.0075M solution of 1:2:2 HF-pyr / THF / pyridine). The reaction mixture was allowed to warm to room temperature. After 20h, H_2O (equal volume as HF-pyridine) was added, and the reaction mixture was heated to 40 °C. After 3h, the reaction mixture was cooled to 0 °C, diluted with EtOAc, and slowly quenched by adding saturated aqueous $NaHCO_3$ dropwise until bubbling ceased. The layers were separated, and the aqueous layer was extracted with 50% EtOAc/Hex. The combined organic layers were dried over $NaSO_4$, filtered, and concentrated. Purification was accomplished by silica gel flash column chromatography (20% EtOAc/Hex to remove silanol, then 50-80% EtOAc/Hex), followed by RP-HPLC (70-100% MeCN/H_2O) affording **SUW206** (6.6 mg, 47% yield) and **SUW207** (1.4 mg, 10% yield). The relative stereochemistry at C13 was based on numerous literature examples demonstrating that *axial protons are upfield of equatorial ones*.

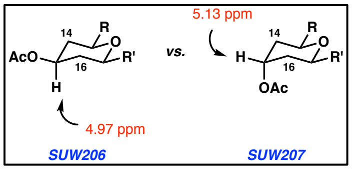

Characterization data for SUW206 (major C13 diastereomer)

TLC R_f = 0.58 (75% EtOAc/Hex, UV active, dark blue spot in *p*-anisaldehyde)

$[\alpha]^{23}_D$ = 3.4° (*c* = 0.13, CH_2Cl_2); **IR** (thin film): 3452 (bs), 2927, 1735, 1718, 1246, 1157, 1075 cm^{-1}

^{1}H-NMR (600 MHz, $CDCl_3$) δ 6.21 – 6.11 (m, 2H, $C_{42}H$, $C_{43}H$), 6.00 (s, 1H, $C_{34}H$), 5.79 (app. d, *J* = 15.6 Hz, 2H, $C_{40}H$, $C_{17}H$), 5.26 (dd, *J* = 15.9, 8.2 Hz, 1H, $C_{16}H$), 5.24 – 5.16 (m, 1H, $C_{25}H$), 5.19 (app. s, 2H, C_{19}-OH, $C_{20}H$), 5.17 – 5.12 (m, 1H, C_7H), 5.02 – 4.94 (m, 1H, $C_{13}H$), 4.32 – 4.20 (m, 2H, C_3-OH, C_5H), 4.19 – 4.10 (m, 2H, C_3H, $C_{15}H$), 4.03 (app. t, *J* = 11.2 Hz, 1H, $C_{23}H$), 3.87 – 3.78 (m, 2H, $C_{26}H$, $C_{11}H$), 3.73 – 3.64 (m, 1H, $C_{22}H_{eq}$), 3.67 (s, 3H), 2.04 (s, 3H), 2.02 (s, 3H), 1.25 (d, *J* = 6.0 Hz, 3H, $C_{27}H_3$), 1.12 (s, 3H), 0.99 (s, 6H), 0.95 – 0.89 (m, 6H)

^{13}C-NMR (125 MHz, $CDCl_3$, 1 signal is obscured by solvent) δ 172.5, 170.9, 170.6, 167.1, 165.7, 152.1, 146.5, 145.7, 139.3, 129.1, 128.5, 119.8, 118.8, 101.9, 99.1, 74.1, 73.8, 72.9, 70.2, 69.9, 69.3, 68.6, 65.9, 64.8, 51.2, 45.0, 42.5, 42.0, 41.1, 40.0, 39.3, 37.9, 35.9, 35.2, 33.5, 31.4, 24.7, 22.0, 21.4, 21.3, 21.1, 19.9, 19.8, 17.0, 13.8

HRMS calculated for $C_{46}H_{68}NaO_{17}$ $[M+Na]^+$: 915.4349; found 915.4322 (TOF ESI+).

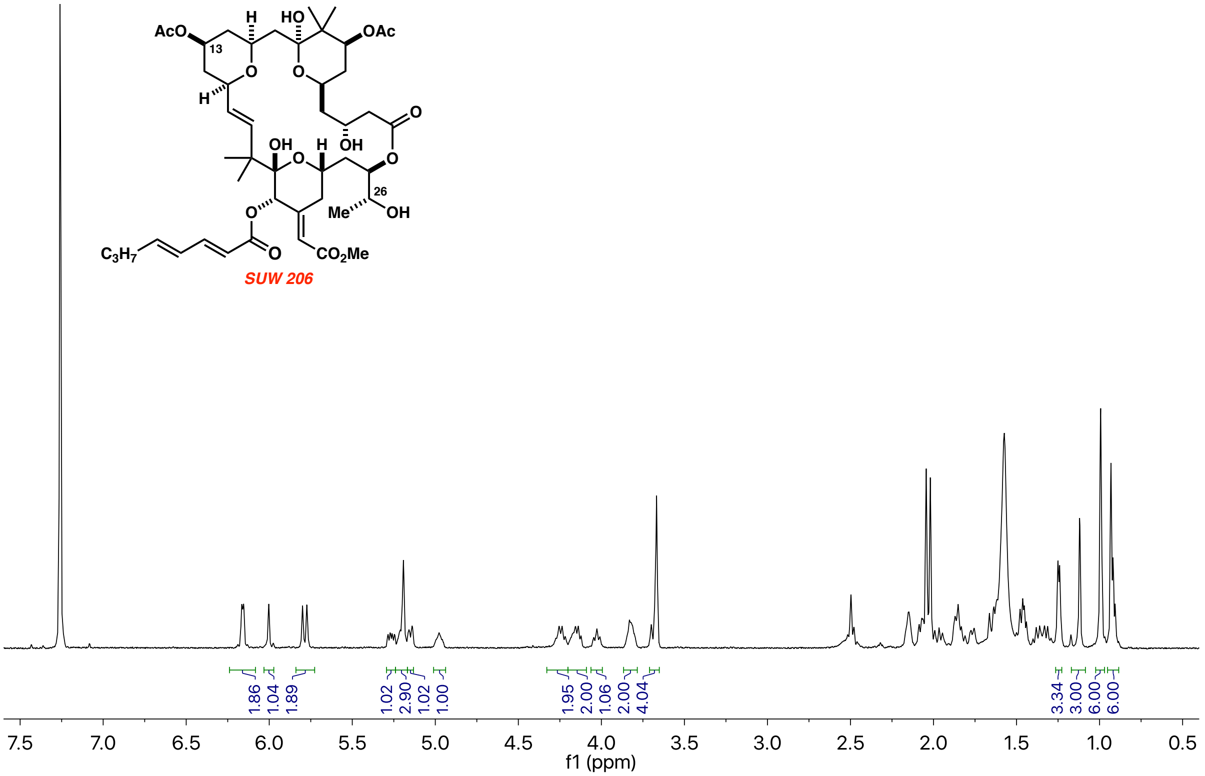

Supplementary Figure 24. ^{1}H NMR (600 MHz, $CDCl_3$) of SUW206.

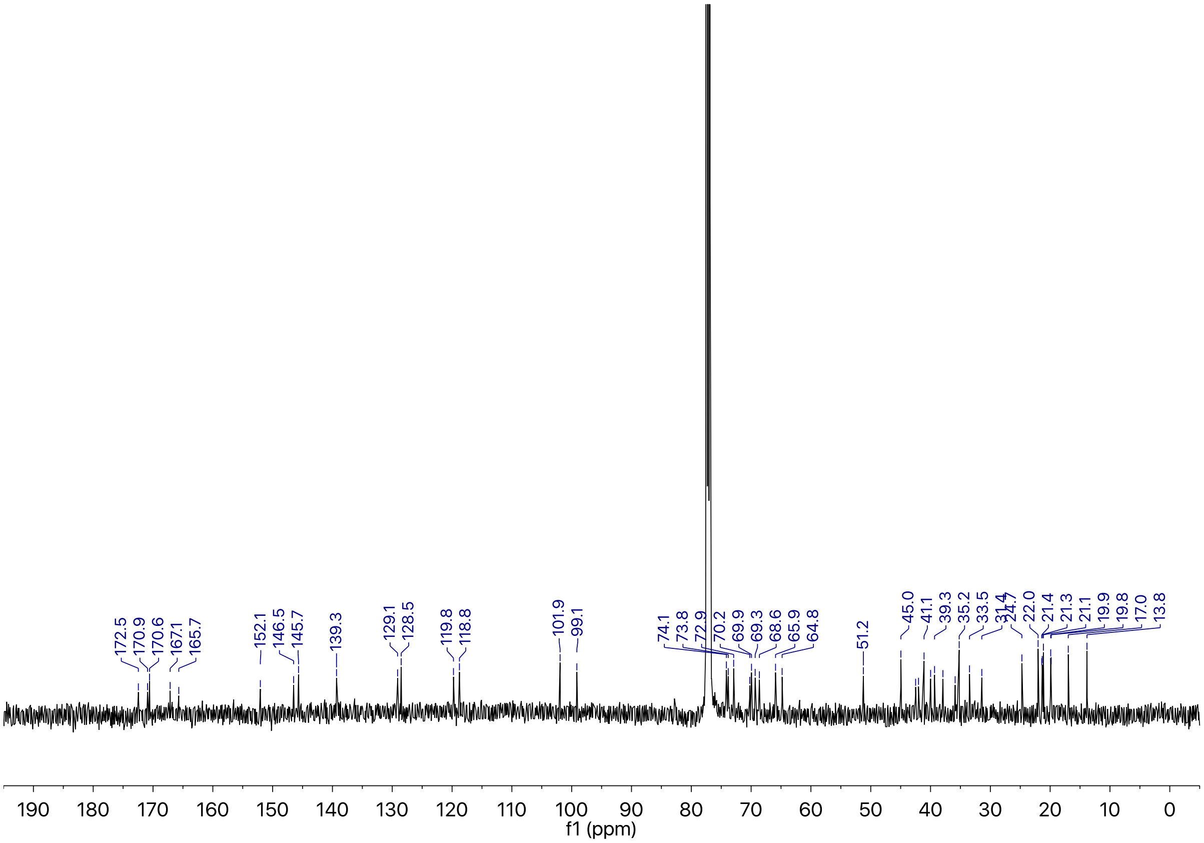

Supplementary Figure 25. ^{13}C-NMR (125 MHz, $CDCl_3$) of SUW206.

Characterization data for SUW207 (minor C13 diastereomer)

TLC R_f = 0.43 (75% EtOAc/Hex, UV active, dark blue spot in *p*-anisaldehyde)

$[\alpha]^{24}_{D}$ = 3.1° (*c* = 0.07, CH_2Cl_2)

IR (thin film): 3463 (bs), 2931, 1743, 1719, 1244, 1158, 1099, 1057, 1029, 1003 cm^{-1}

^{1}H-NMR (600 MHz, $CDCl_3$) δ 6.20 – 6.12 (m, 2H, $C_{42}H$, $C_{43}H$), 6.01 (s, 1H, $C_{34}H$), 5.80 (d, *J* = 15.1 Hz, 1H), 5.75 (d, *J* = 15.8 Hz, 1H), 5.32 – 5.07 (m, 6H), 4.38 (app. t, *J* = 10.3 Hz, 1H), 4.34 – 4.29 (m, 1H), 4.26 (app. t, *J* = 11.4 Hz, 1H), 4.22 – 4.15 (m, 1H), 4.09 – 4.00 (m, 2H), 3.88 – 3.80 (m, 1H), 3.72 – 3.68 (m, 1H), 3.67 (s, 3H), 2.17 (s, 3H), 2.05 (s, 3H), 1.24 (d, *J* = 5.7 Hz, 3H, $C_{27}H_3$), 1.13 (s, 3H), 1.00 (s, 6H), 0.96 – 0.89 (m, 6H)

^{13}C-NMR (125 MHz, C_6D_6, 2 signals are obscured by solvent) δ 173.1, 169.9, 169.8, 166.8, 165.6, 152.7, 146.6, 145.1, 139.5, 120.6, 119.3, 102.0, 99.9, 74.9, 74.5, 74.3, 72.8, 70.5, 68.8, 67.8, 66.0, 65.8, 65.3, 50.6, 45.5, 42.5, 42.3, 41.2, 39.7, 37.5, 36.4, 36.1, 35.1, 33.6, 32.1, 25.3, 22.0, 21.2, 21.1, 20.7, 20.1, 19.8, 17.0, 13.7

HRMS calculated for $C_{46}H_{68}NaO_{17}$ $[M+Na]^+$: 915.4349; found 915.4320 (TOF ESI+).

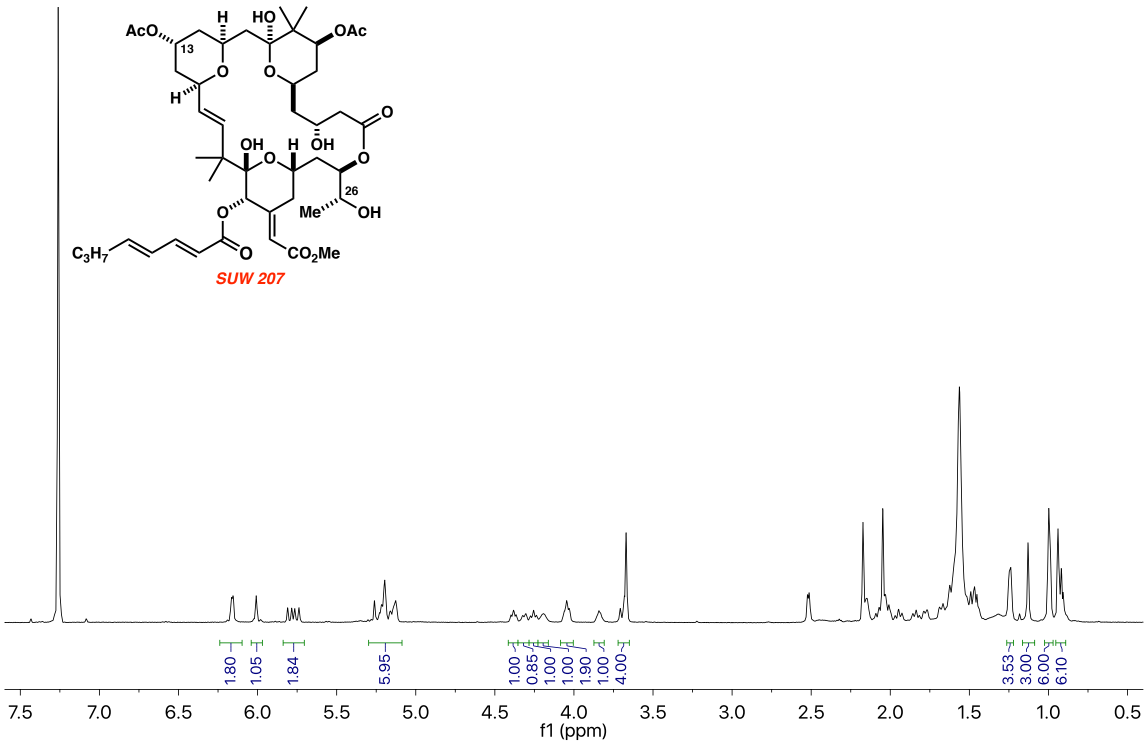

Supplementary Figure 26. ^{1}H NMR (600 MHz, $CDCl_3$) of SUW207.

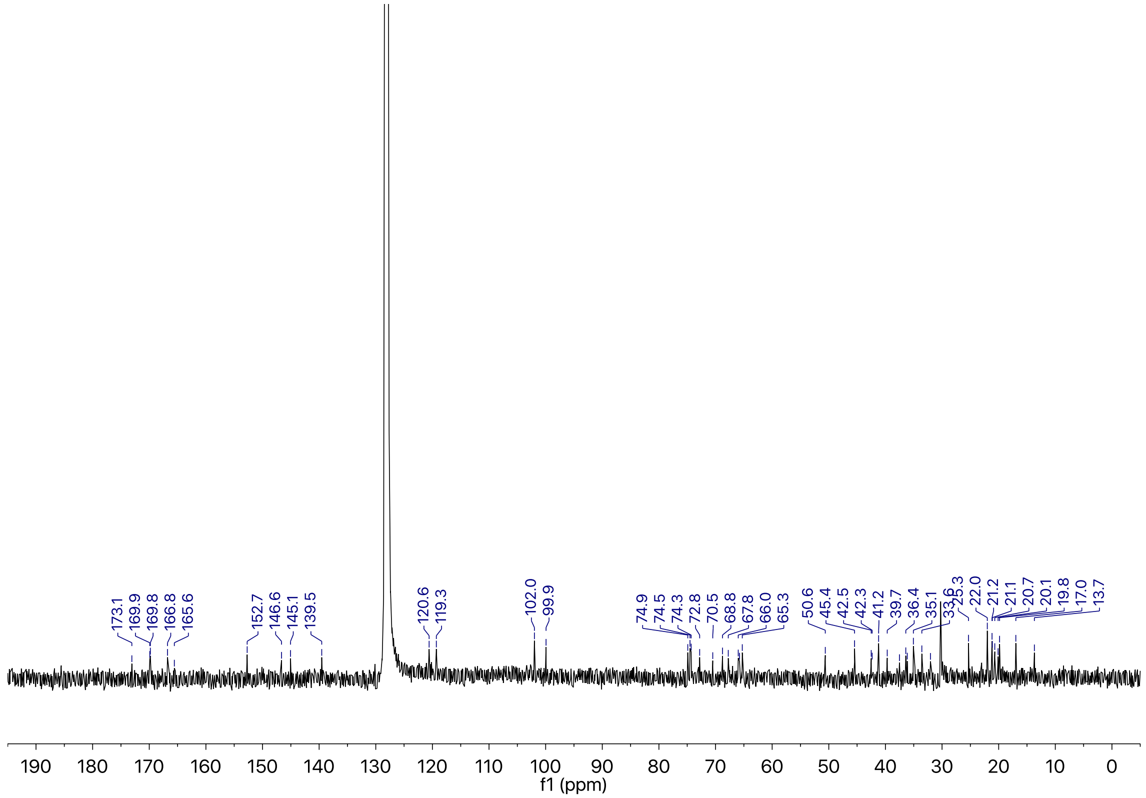

Supplementary Figure 27. ^{13}C NMR (125 MHz, C_6D_6) of SUW207.

SUW208

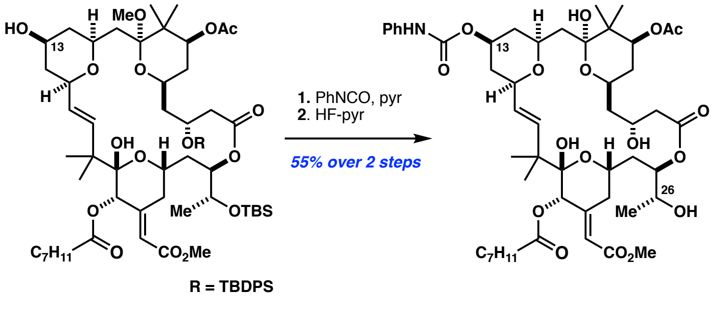

Ref: A. B. Smith et al., Design, Synthesis, and Evaluation of Carbamate-Substituted Analogues of (+)-Discodermolide. *Org. Lett.* **2005**, *7*, 311–314.

Acylation: To a solution of **SI-4** (15 mg, 0.012 mmol, 1 equiv) in 2:1 CH_2Cl_2 / pyridine (1.23 mL, 0.01M) was added phenyl isocyanate (40 uL, 0.369 mmol, 30 equiv). After 48h, the reaction mixture was quenched by adding saturated aqueous NH_4Cl (2 mL). The layers were separated, and the aqueous layer was extracted with 80% EtOAc/Hex (3x5 mL). The combined organic layers were dried over $NaSO_4$, filtered, and concentrated. Purification was accomplished by silica gel flash column chromatography (20% EtOAc/Hex) affording the C13 carbamate (assume quantitative yield). **LRMS** calculated for $C_{74}H_{105}NNaO_{17}Si_2$ $[M+Na]^+$: 1358.7; found 1359.0 (TOF ESI+).

Global deprotection: To a cooled (0 °C) solution of this carbamate (assume 0.012 mmol, 1 equiv) in 1:1 THF / pyridine (1.31 mL) was added 70% HF-pyridine (328 uL), affording a 0.0075M solution of 1:2:2 HF-pyr / THF / pyridine. The reaction mixture was allowed to warm to room temperature. After 20h, H_2O (328 uL) was added, and the reaction mixture was heated to 40 °C. After 2h, the reaction mixture was cooled to 0 °C, diluted with 50% EtOAc/Hex (5 mL), and slowly quenched by adding saturated aqueous $NaHCO_3$ dropwise until bubbling ceased. The layers were separated, and the aqueous layer was extracted with 80% EtOAc/Hex (5x5 mL). The combined organic layers were dried over $NaSO_4$, filtered, and concentrated. Purification was accomplished by silica gel flash column chromatography (25-50% EtOAc/Hex), followed by RP-HPLC (60-100% MeCN/H_2O) affording **SUW208** (6.6 mg, 55% yield over 2 steps).

Characterization data for SUW208

TLC R_f = 0.36 (60% EtOAc/Hex, UV active, stains in CAM)

$[\alpha]^{23}_D$ = 6.9° (*c* = 0.10, CH_2Cl_2)

IR (thin film): 3455 (bs), 2927, 1715, 1601, 1444, 1366, 1313, 1233, 1158, 1055 cm^{-1}

^{1}H-NMR (500 MHz, $CDCl_3$) δ 7.42 – 7.32 (m, 3H, $C_{42}H$), 7.30 (t, *J* = 7.9 Hz, 2H), 7.09 – 7.03 (m, 1H), 6.58 (t, *J* = 11.3 Hz, 1H, $C_{41}H$), 6.55 (s, 1H), 6.09 (dt, *J* = 14.7, 7.1 Hz, 1H, $C_{43}H$), 6.02 (d, *J* = 1.9 Hz, 1H, $C_{34}H$), 5.80 (d, *J* = 15.8 Hz, 1H, $C_{17}H$), 5.55 (d, *J* = 11.3 Hz, 1H, $C_{40}H$), 5.28 (dd, *J* = 15.8, 8.4 Hz, 1H, $C_{16}H$), 5.22 (s, 1H), 5.23 – 5.17 (m, 1H, $C_{25}H$), 5.19 (s, 1H), 5.15 (dd, *J* = 11.7, 4.7 Hz, 1H, C_7H), 5.04 – 4.94 (m, 1H, $C_{13}H$), 4.33 – 4.12 (m, 4H, C_3-OH, C_5H, C_3H, $C_{15}H$), 4.08 – 4.00 (m, 1H, $C_{23}H$), 3.90 – 3.79 (m, 2H, $C_{11}H$, $C_{26}H$), 3.73 – 3.63 (m, 1H, $C_{22}H_{eq}$), 3.68 (s, 3H), 2.70 (bs, 1H, C_9-OH), 2.57 – 2.46 (m, 2H, C_2H_2), 2.04 (s, 3H, C_7-OAc), 1.12 (s, 3H), 1.01 (s, 3H), 0.99 (s, 3H), 0.94 – 0.90 (m, 6H)

^{13}C-NMR (125 MHz, $CDCl_3$, 2 signals obscured by solvent) δ 172.6, 171.0, 167.2, 165.7, 152.0, 151.0, 146.5, 145.7, 139.4, 137.8, 129.2, 129.1, 128.5, 123.7, 119.8, 118.7, 101.9, 99.2, 74.1, 73.9, 73.0, 70.3, 69.4, 68.6, 65.9, 64.8, 51.3, 45.0, 42.5, 42.0, 41.1, 40.0, 39.7, 38.2, 35.9, 35.2, 33.5, 24.7, 22.0, 21.3, 21.1, 19.9, 19.8, 17.0, 13.8

HRMS calculated for $C_{51}H_{71}NaNO_{17}$ $[M+Na]^+$: 992.4614; found 992.4585 (TOF ESI+)

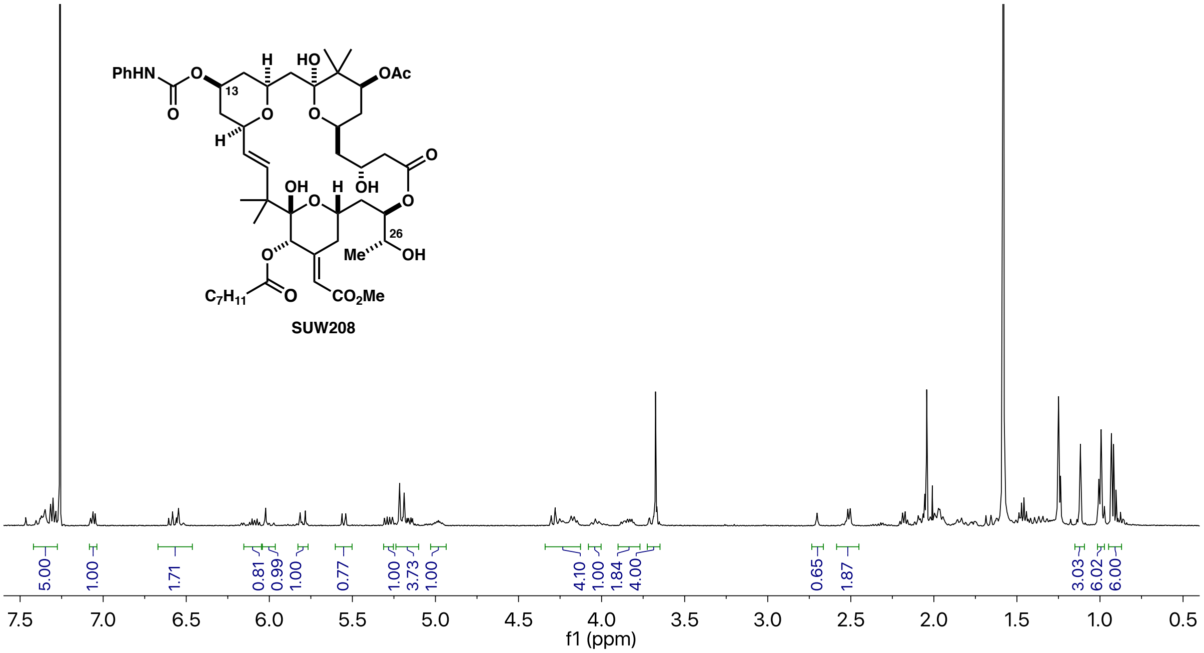

Supplementary Figure 28. ^{1}H-NMR (500 MHz, $CDCl_3$) of SUW208.

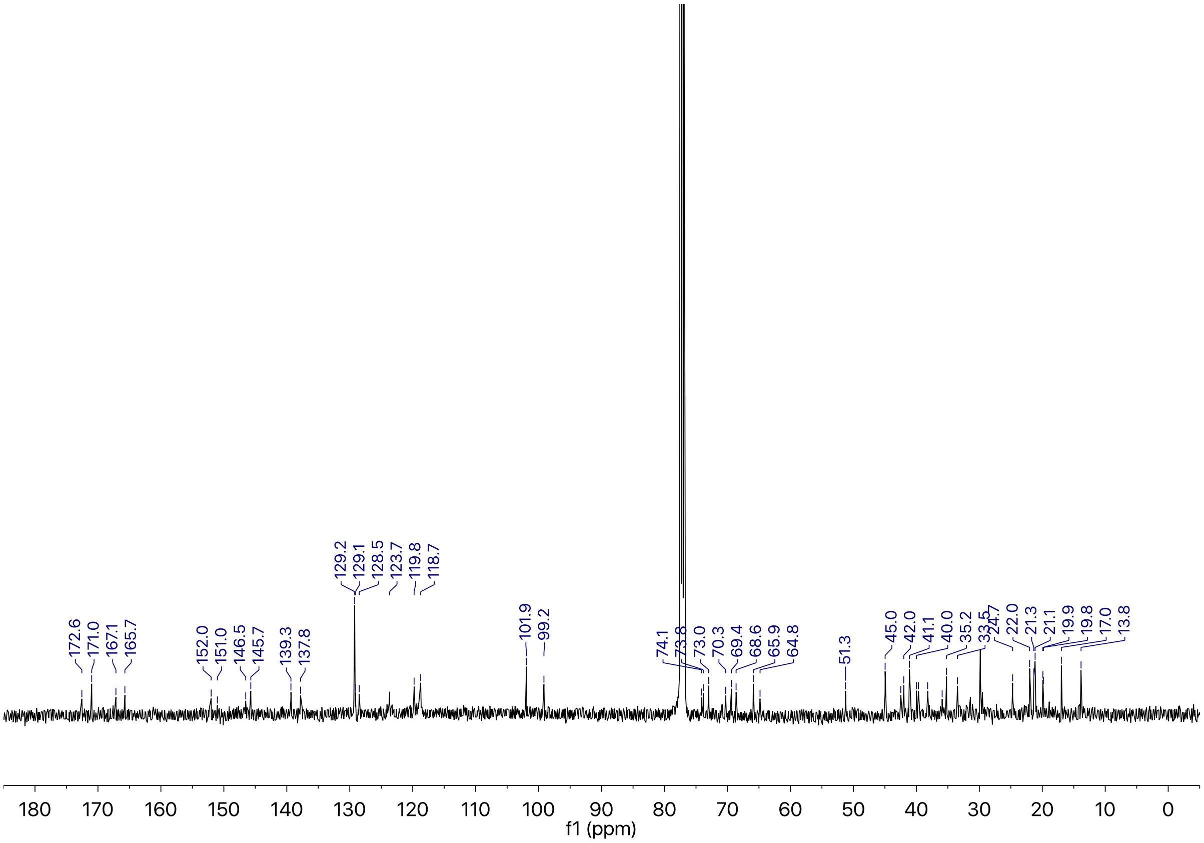

Supplementary Figure 29. ^{13}C-NMR (125 MHz, $CDCl_3$) of SUW208.

SUW209

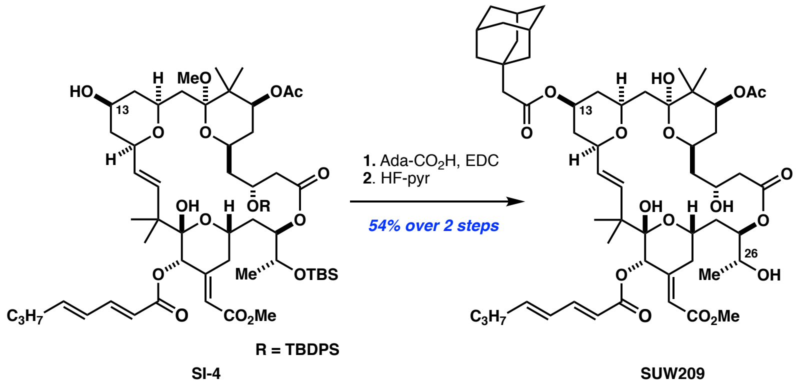

Acylation: To a solution of alcohol **SI-4** (10 mg, 0.0082 mmol, 1 equiv) in CH_2Cl_2 (1 mL) was sequentially added 1-adamantaneacetic acid (8 mg, 0.041 mmol, 5 equiv), EDC-HCl (7.9 mg, 0.041 mmol, 5 equiv), and DMAP (1 crystal). After 20h, TLC analysis indicated complete conversion of starting material. The reaction mixture was directly flashed via silica gel flash column chromatography (10% EtOAc/Hex) affording the C13-adamantyl ester in quantitative yield.

Global deprotection: To a cooled (0 °C) solution of this ester (1 equiv) in 1:1 THF / pyridine (1.5 mL) was added 70% HF-pyridine (375 uL), affording a 1:2:2 HF-pyr / THF / pyridine solution. The reaction mixture was allowed to warm to room temperature. After 24h, H_2O (300 uL) was added, and the reaction mixture was heated to 30 °C. After 4h, the reaction mixture was cooled to 0 °C, diluted with EtOAc (10 mL), and slowly quenched by adding saturated aqueous $NaHCO_3$ dropwise until bubbling ceased. The layers were separated, and the aqueous layer was extracted with 80% EtOAc/Hex (5x10 mL). The combined organic layers were dried over $NaSO_4$, filtered, and concentrated. Purification was accomplished by silica gel flash column chromatography (40-100% EtOAc/Hex), followed by RP-HPLC (70-100% MeCN/H_2O) affording **SUW209** (4.5 mg, 54% yield).

Characterization data for SUW209

TLC R_f = 0.33 (50% EtOAc/Hex, UV active, dark blue spot in *p*-anisaldehyde)

$[\alpha]^{24}_{D}$ = 12.5° (*c* = 0.125, CH_2Cl_2)

IR (thin film): 3461 (bs), 2906, 2849, 1720, 1640, 1366, 1245, 1158, 1137, 1100, 1058, 1003 cm^{-1}

^{1}H-NMR (600 MHz, $CDCl_3$) δ 6.20 – 6.09 (m, 2H, $C_{42}H$, $C_{43}H$), 6.00 (bs, 1H, $C_{34}H$), 5.79 (d, *J* = 15.6 Hz, 1H), 5.78 (d, *J* = 15.6 Hz, 1H), 5.31 – 5.12 (m, 5H), 5.02 – 4.94 (m, 1H, $C_{13}H$), 4.31 – 4.10 (m, 4H), 4.07 – 3.98 (m, 1H), 3.86 – 3.78 (m, 2H), 3.73 – 3.64 (m, 1H, $C_{22}H_{eq}$), 3.67 (s, 3H), 2.04 (s, 3H, C_7-OAc), 2.02 (s, 2H), 1.97 (s, 3H), 1.59 (s, 6H), 1.57 (s, 6H), 1.24 (d, *J* = 6.4 Hz, 3H, $C_{27}H_3$), 1.13 (s, 3H), 0.99 (s, 6H), 0.96 – 0.87 (m, 6H)

^{13}C-NMR (125 MHz, $CDCl_3$, 1 signal obscured by solvent) δ 172.5, 171.3, 170.9, 167.1, 165.7, 152.1, 146.5, 145.7, 139.3, 129.1, 128.5, 119.8, 118.8, 102.0, 99.1, 74.1, 73.9, 72.9, 70.2, 69.5, 69.4, 68.6, 65.9, 64.8, 51.2, 49.3, 45.0, 42.6 (3C), 42.0, 41.1, 40.0, 39.6, 38.1, 36.9 (3C), 35.9, 35.2, 33.5, 33.0, 31.4, 28.7 (3C), 24.7, 22.2, 22.0, 21.3, 21.2, 19.9, 19.8, 17.0, 13.8

HRMS calculated for $C_{56}H_{82}NaO_{17}$ $[M+Na]^+$: 1049.5444; found 1049.5413 (TOF ESI+).

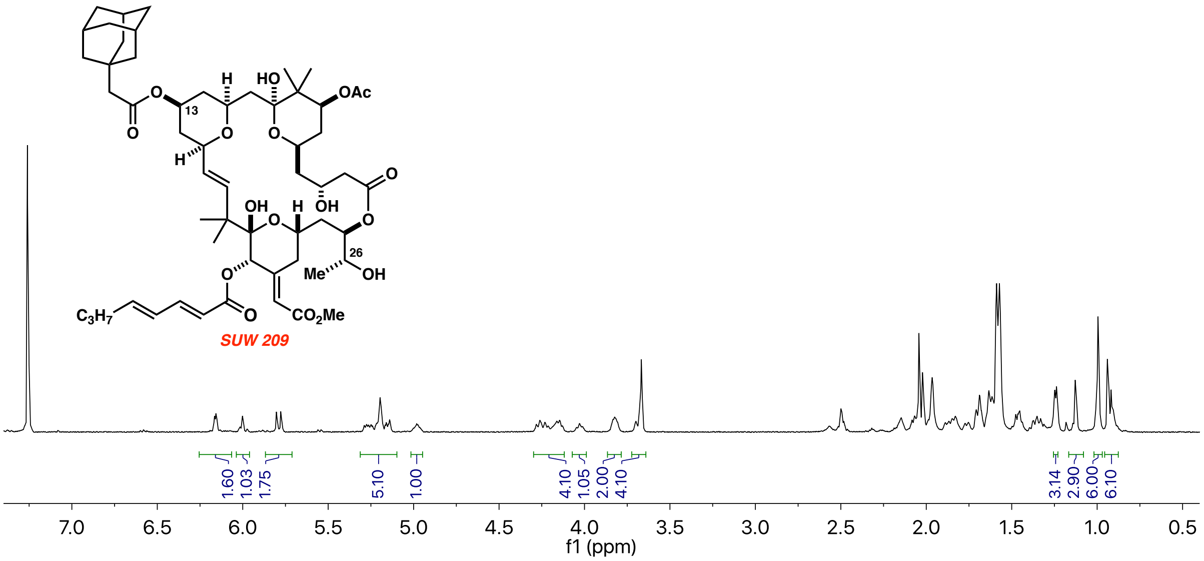

Supplementary Figure 30. ^{1}H NMR (600 MHz, $CDCl_3$) of SUW209.

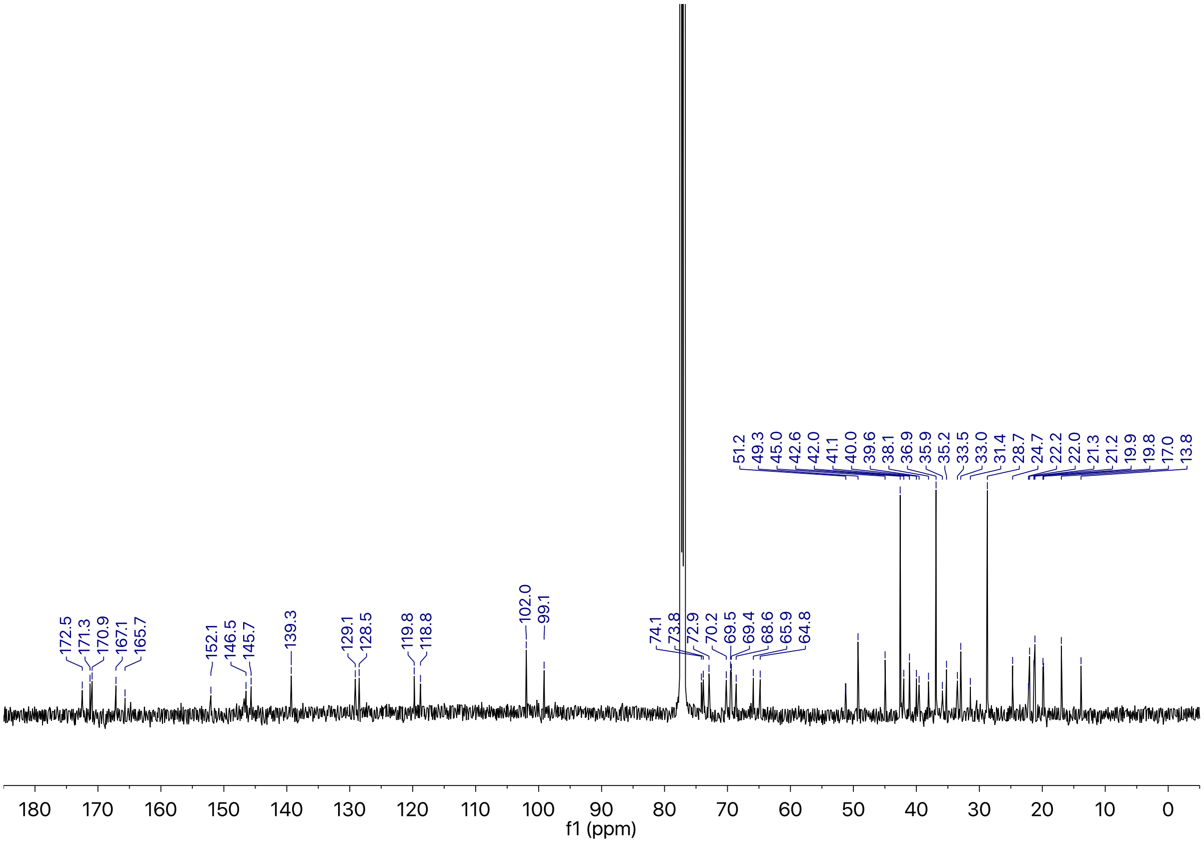

Supplementary Figure 31. ^{13}C NMR (125 MHz, $CDCl_3$) of SUW209.

SUW210

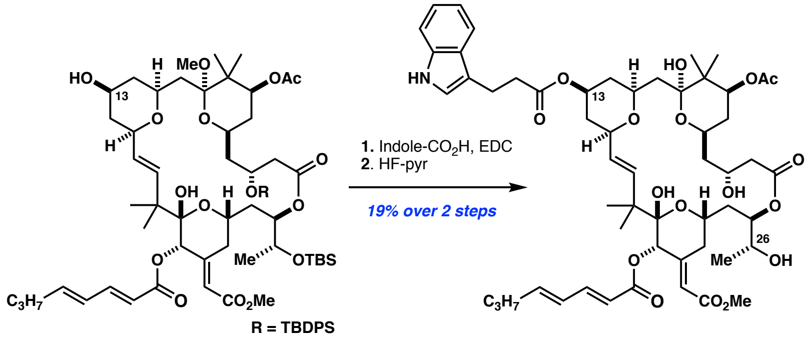

SI-4 SUW210

Acylation: To a solution of alcohol **SI-4** (10 mg, 0.008 mmol, 1 equiv) in CH_2Cl_2 (0.5 mL) was sequentially added indole-3-propionic acid (8 mg, 0.041 mmol, 5 equiv), EDC-HCl (8 mg, 0.0041 mmol, 5 equiv), and DMAP (1 crystal). After 24h, the reaction mixture was directly flashed via silica gel flash column chromatography (20% EtOAc/Hex) affording the C13-indole ester (assume quantitative yield).

Global deprotection: To a cooled (0 °C) solution of this ester (assume 0.008 mmol, 1 equiv) in 1:1 THF / pyridine (400 uL) was added 70% HF-pyridine (200 uL), affording a 1:2:2 HF-pyr / THF / pyridine solution. The reaction mixture was allowed to warm to room temperature. After 36h, H_2O (200 uL) was added, and the reaction mixture was heated to 40 °C. After 4h, the reaction mixture was cooled to 0 °C, diluted with EtOAc (10 mL), and slowly quenched by adding saturated aqueous $NaHCO_3$ dropwise until bubbling ceased. The layers were separated, and the aqueous layer was extracted with 80% EtOAc/Hex (5x10 mL). The combined organic layers were dried over $NaSO_4$, filtered, and concentrated. Purification was accomplished by silica gel flash column chromatography (50-80% EtOAc/Hex), followed by RP-HPLC (60-100% MeCN/H_2O) affording **SUW210** (1.6 mg, 19% over 2 steps).

Characterization data for SUW210

TLC R_f = 0.53 (75% EtOAc/Hex, UV active, purple spot in *p*-anisaldehyde)

$[\alpha]^{24}_D$ = 11.2° (*c* = 0.085, CH_2Cl_2)

IR (thin film): 3454 (bs), 2922, 2851, 1721, 1461, 1366, 1260, 1158, 1098, 1027 cm^{-1}

^{1}H-NMR (600 MHz, $CDCl_3$) δ 7.96 (bs, 1H, NH), 7.59 (d, *J* = 7.8 Hz, 1H), 7.35 (d, *J* = 8.0 Hz, 1H), 7.18 (app. t, *J* = 7.1 Hz, 1H), 7.11 (app. t, *J* = 7.4 Hz, 1H), 7.00 (s, 1H), 6.21 – 6.11 (m, 2H, $C_{42}H$, $C_{43}H$), 6.00 (d, *J* = 2.0 Hz, 1H, $C_{34}H$), 5.78 (app. t, *J* = 15.1 Hz, 2H, $C_{40}H$, $C_{17}H$), 5.25 (dd, *J* = 15.8, 8.4 Hz, 1H, $C_{16}H$), 5.24 – 5.17 (m, 1H), 5.20 (s, 1H, $C_{20}H$), 5.15 (dd, *J* = 11.8, 4.8 Hz, 1H), 5.01 – 4.93 (m, 1H, $C_{13}H$), 4.23 (t, *J* = 11.8 Hz, 1H), 4.20 – 4.14 (m, 1H), 4.11 (t, *J* = 10.0 Hz, 1H), 4.07 – 3.99 (m, 1H), 3.89 – 3.80 (m, 1H), 3.81 – 3.74 (m, 1H), 3.72 – 3.64 (m, 1H), 3.67 (s, 3H), 3.08 (t, *J* = 7.5 Hz, 2H), 2.68 (t, *J* = 7.6 Hz, 2H), 2.05 (s, 3H, C7-OAc), 1.12 (s, 3H), 0.99 (s, 6H), 0.94 – 0.90 (m, 6H)

^{13}C-NMR (125 MHz, $CDCl_3$, 1 signal obscured by solvent) δ 173.0, 172.6, 171.0, 167.1, 165.7, 152.0, 146.6, 145.8, 139.2, 136.4, 129.1, 128.5, 127.3, 122.2, 121.6, 119.8, 119.5, 118.9, 118.7, 115.0, 111.2, 101.9, 99.2, 74.1, 73.9, 73.0, 70.3, 69.9, 69.3, 68.6, 65.9, 64.8, 51.3, 45.0, 42.5, 42.0, 41.1, 40.0, 39.3, 37.9, 35.9, 35.4, 35.2, 33.5, 31.4, 24.7, 22.0, 21.3, 21.1, 20.8, 19.9, 19.8, 17.0, 13.9

HRMS calculated for $C_{55}H_{75}NaNO_{17}$ $[M+Na]^+$: 1044.4927; found 1044.4897 (TOF ESI+)

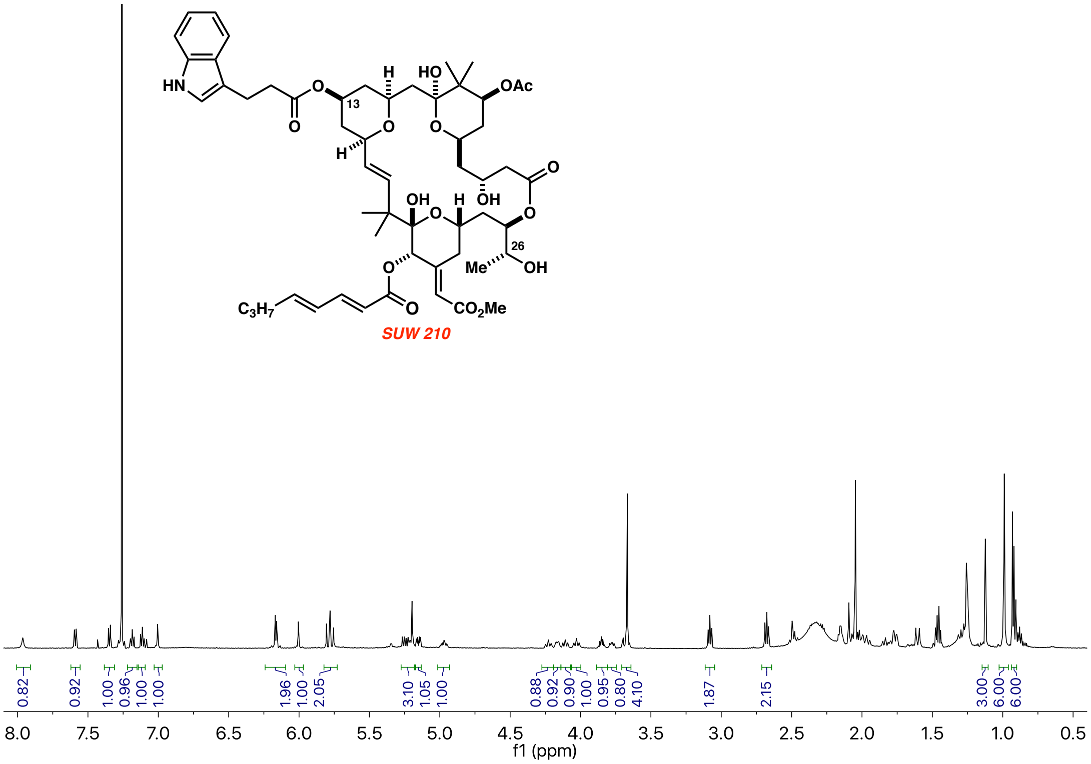

Supplementary Figure 32. ^{1}H-NMR (600 MHz, $CDCl_3$) of SUW210.

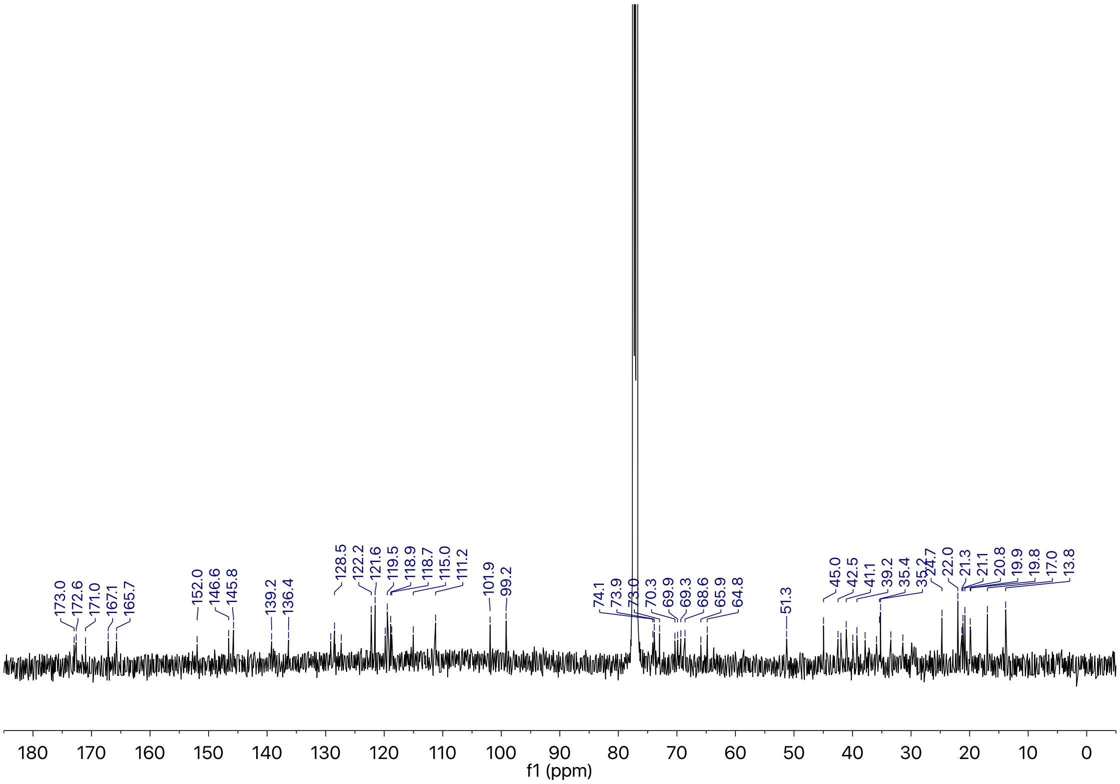

Supplementary Figure 33. ^{13}C-NMR (125 MHz, $CDCl_3$) of SUW210.

SUW211

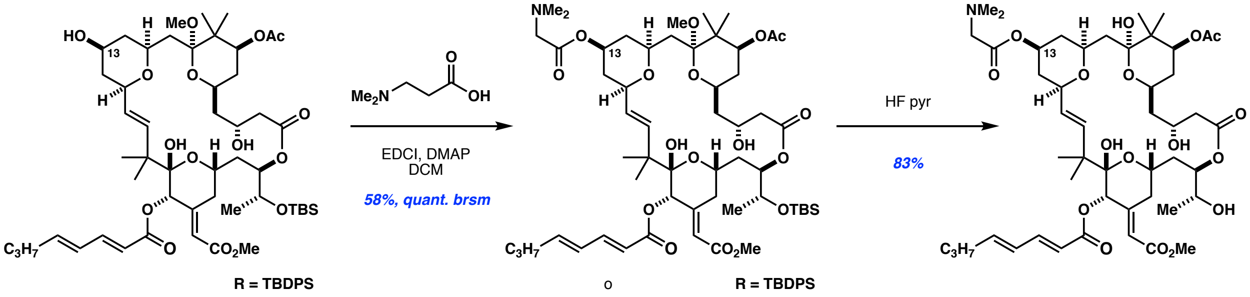

SI-4 SI-5 SUW211

Acylation: To a vial containing **SI-4** (16 mg, 0.016 mmol, 1 equiv) in DCM (0.5 mL) was added dimethylglycine (8.4 mg, 0.082 mmol, 5 equiv), EDCI (16 mg, 0.016 mmol, 5 equiv) and DMAP (10 mg, .082 mmol, 5 equiv). After stirring at room temperature for 16 h, the mixture was partitioned between DCM and saturated $NaHCO_3$. After extraction with DCM (2x), the combined organics were dried over Na_2SO_4, filtered and concentrated. Flash chromatography (30-40% EtOAc/hexane) provided glycinate **SI-5** as a white residue (10 mg, 58% yield, quant. brsm), which was carried forward to the next reaction.

Deprotection: Glycinate **SI-5** was dissolved in 1:1 THF:pyridine (0.6 mL) in a polypropylene vial. HF:pyridine (0.2 mL) was added and the reaction mixture was stirred at 40° C for 20 h, whereupon water (0.2 mL) was added and the resulting mixture stirred at the same temperature for 2.5 h. The reaction was quenched with sat. $NaHCO_3$, extracted with EtOAc (2x) and the combined organics dried over Na_2SO_4. Flash chromatography (5-10% MeOH/DCM) provided **SUW211** as a white residue (6 mg, 83% yield).

Characterization data for SUW211

TLC R_f = 0.5 (10% MeOH/DCM, UV active, purple spot in *p*-anisaldehyde)

$[\alpha]^{22.7}_{D}$ = 8.2° (*c* = 0.23, CH_2Cl_2)

IR (thin film): 3457, 3396, 3376, 2957, 2927, 1735, 1720, 1655, 1407, 1324, 1244, 1079, 1003 cm-1

^{1}H NMR (600 MHz, $CDCl_3$) δ 7.39 – 7.30 (m, 1H), 6.14 (d, *J* = 5.5 Hz, 2H), 5.98 (s, 1H), 5.76 (dd, *J* = 15.5, 4.0 Hz, 2H), 5.27 (s, 1H), 5.24 (dd, *J* = 15.8, 8.3 Hz, 1H), 5.17 (m, 3H), 5.11 (m, 2H), 4.26 (d, *J* = 12.1 Hz, 1H), 4.12 (m, 1H), 4.00 (t, *J* = 11.2 Hz, 1H), 3.89 (t, *J* = 9.6 Hz, 1H), 3.80 – 3.76 (m, 1H), 3.69 – 3.59 (m, 4H), 3.46 (m, 1H), 2.67 (bs, 4H), 2.51 – 2.46 (m, 2H), 2.13 (m, 2H), 2.02 (m, 5H), 1.98 (d, *J* = 13.2 Hz, 1H), 1.91 (t, *J* = 13.0 Hz, 2H), 1.83 (m, 2H), 1.73 (m, 1H), 1.68 (d, *J* = 15.1 Hz, 1H), 1.59 (m, 1H), 1.47 – 1.40 (m, 4H), 1.35 (d, *J* = 11.7 Hz, 1H), 1.29 – 1.26 (m, 1H), 1.24 – 1.21 (m, 5H), 1.10 (s, 3H), 0.97 (m, 5H), 0.90 (m, 6H), 0.86 (t, *J* = 7.1 Hz, 2H).

^{13}C NMR (126 MHz, $CDCl_3$) δ 171.17, 167.27, 165.80, 152.16, 146.62, 145.82, 141.22, 139.52, 138.54, 129.19, 128.62, 119.89, 118.86, 102.13, 99.23, 74.21, 74.07, 73.13, 70.32, 69.41, 68.76, 65.93, 64.90, 51.35, 45.06, 44.44, 42.73, 42.03, 41.28, 40.13, 39.14, 37.97, 35.33, 34.39, 33.56, 31.53, 29.98, 24.85, 22.61, 22.13, 21.48, 21.32, 19.95, 19.79, 17.16, 14.34, 13.96.

HRMS calculated for $C_{48}H_{73}NO_{17}Na$ [M+Na]+: 936.4951; found 936.4950 (TOF ESI+)

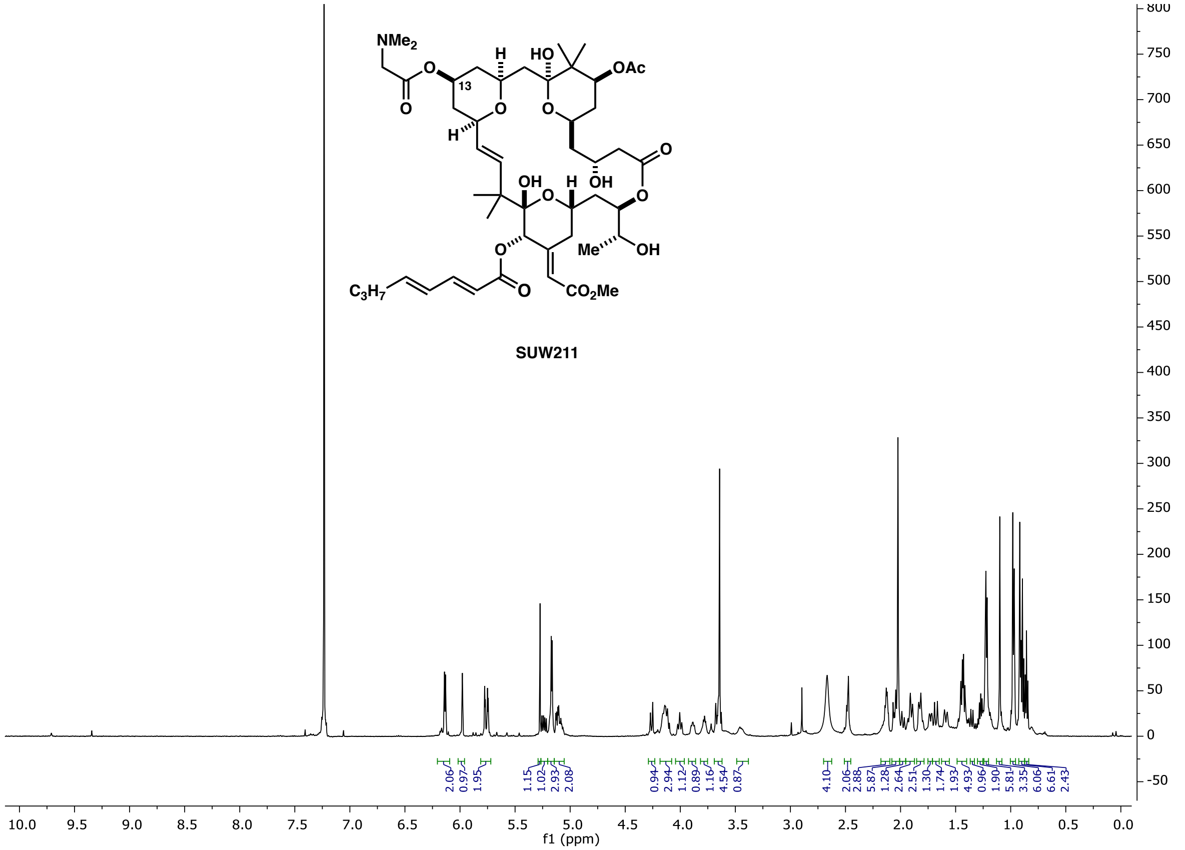

Supplementary Figure 34. ^1H NMR (600 MHz, $CDCl_3$) of SUW211.

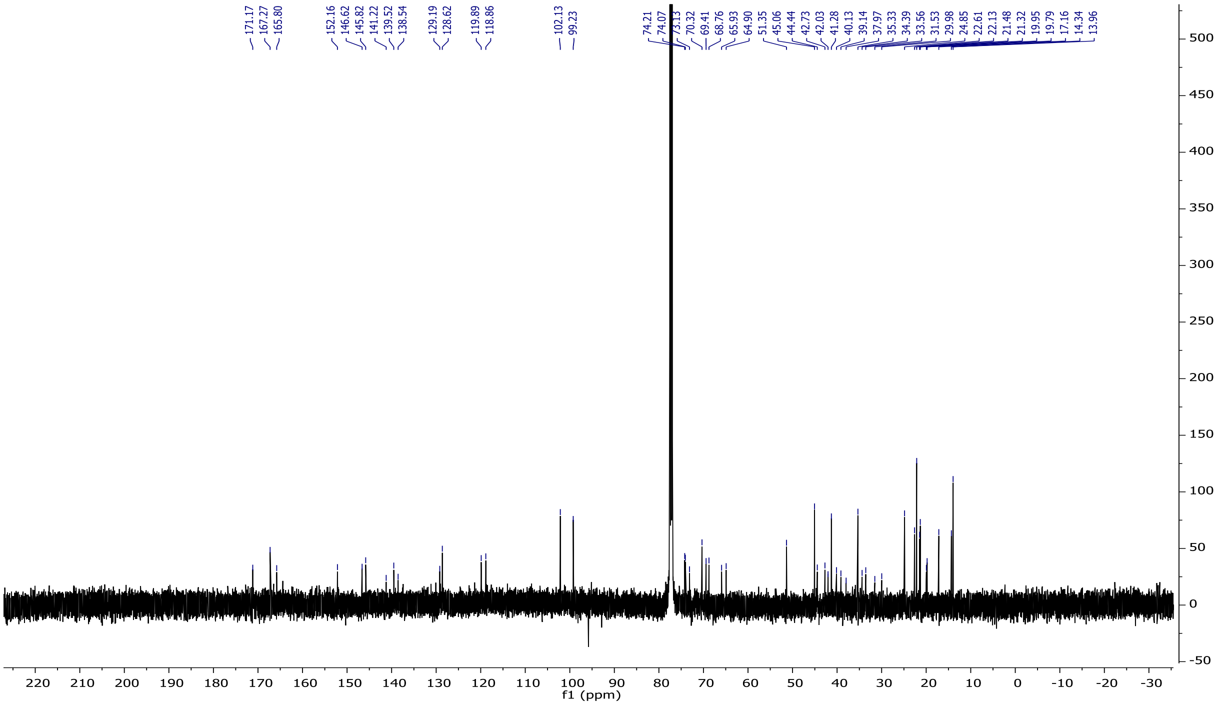

Supplementary Figure 35. ^{13}C NMR (126 MHz, $CDCl_3$) of SUW211.

SUW212

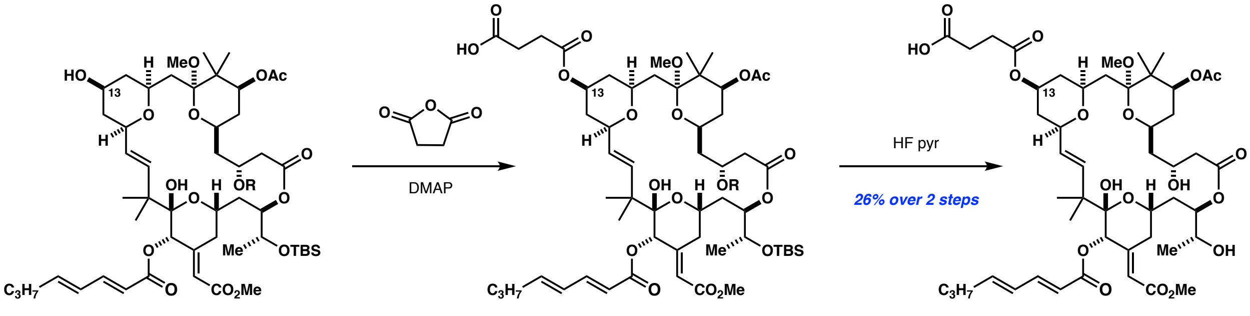

R = TBDPS
SI-4

R = TBDPS
SI-6

SUW212

Acylation: To a vial charged with **SI-4** (11 mg, 0.011 mmol, 1 equiv) in DCM (0.5 mL) was added succinic anhydride (3.4 mg, 0.034 mmol, 3 equiv) and DMAP (4.1 mg, 0.034 mmol, 3 equiv). After 16 h of stirring, the reaction was partitioned between DCM and saturated NH_4Cl. After extraction with DCM (2x), the combined organics were dried over Na_2SO_4, filtered and concentrated. Flash chromatography (50% EtOAc/hexane) provided succinate **SI-6** as a white residue (8 mg).

Deprotection: Succinate **SI-6** was dissolved in 1:1 THF:pyridine (0.48 mL) in a polypropylene tube. HF-pyridine (0.12 mL) was added down the side of the tube. The reaction mixture was heated at 40 °C in an oil bath for 20 h, at which point H_2O (0.1 mL) was added. After an additional 2.5 h of stirring at 40 C, the reaction was partitioned between H_2O and EtOAc. Following extraction with EtOAc (3x), the combined organics were concentrated and purified by flash chromatography (10% MeOH/DCM) to provide **SUW212** (2.7 mg, 26% over 2 steps).

Characterization data for SUW212

TLC R_f = 0.6 (10% MeOH/DCM, UV-active, purple spot in *p*-anisaldehyde)

$[\alpha]^{23.1}_D$ = 3.0° (*c* = 0.27, CH_2Cl_2)

IR (thin film): 3461, 2956, 2927, 1734, 1717, 1636, 1617, 1559, 1364, 1245, 1159, 1100, 1002 cm-1

^{1}H NMR (600 MHz, $CDCl_3$) δ 7.39 – 7.30 (m, 1H), 6.19 – 6.10 (m, 2H), 5.98 (d, *J* = 2.0 Hz, 1H), 5.74 (dd, *J* = 29.8, 15.5 Hz, 2H), 5.27 (s, 1H), 5.24 (dd, *J* = 15.8, 8.3 Hz, 1H), 5.20 – 5.11 (m, 4H), 4.97 (td, *J* = 11.1, 5.6 Hz, 1H), 4.33 (m, 1H), 4.20 – 4.10 (m, 2H), 4.07 (t, *J* = 9.9 Hz, 1H), 4.01 (t, *J* = 11.1 Hz, 1H), 3.95 – 3.89 (m, 1H), 3.78 (p, *J* = 6.4 Hz, 1H), 3.64 (m, 4H), 2.75 – 2.51 (m, 4H), 2.44 (d, *J* = 12.2 Hz, 1H), 2.13 (q, *J* = 7.1 Hz, 2H), 2.08 – 1.94 (m, 7H), 1.90 (t, *J* = 11.9 Hz, 1H), 1.86 – 1.77 (m, 3H), 1.75 – 1.69 (m, 1H), 1.67 (d, *J* = 15.0 Hz, 1H), 1.60 – 1.52 (m, 1H), 1.49 – 1.40 (m, 3H), 1.35 – 1.25 (m, 3H), 1.24 – 1.20 (m, 6H), 1.10 (s, 3H), 0.98 (s, 3H), 0.96 (s, 3H), 0.94 (s, 3H), 0.89 (t, *J* = 7.4 Hz, 3H), 0.86 (t, *J* = 7.2 Hz, 2H).

^{13}C NMR (126 MHz, $CDCl_3$) δ 171.74, 171.32, 167.33, 165.84, 152.27, 146.67, 146.63, 145.82, 129.63, 128.65, 119.88, 118.92, 102.17, 99.27, 95.83, 74.27, 74.21, 73.35, 70.23, 69.40, 68.81, 65.81, 64.91, 51.38, 46.59, 45.05, 42.63, 41.33, 40.22, 39.20, 38.13, 35.78, 35.35, 33.63, 31.57, 30.00, 28.86, 24.89, 22.59, 22.15, 21.52, 21.42, 20.04, 19.65, 18.15, 17.22, 13.98.

HRMS calculated for $C_{48}H_{70}O_{19}$ [M+Na]+: 973.4404; found 973.4384 (TOF ESI+)

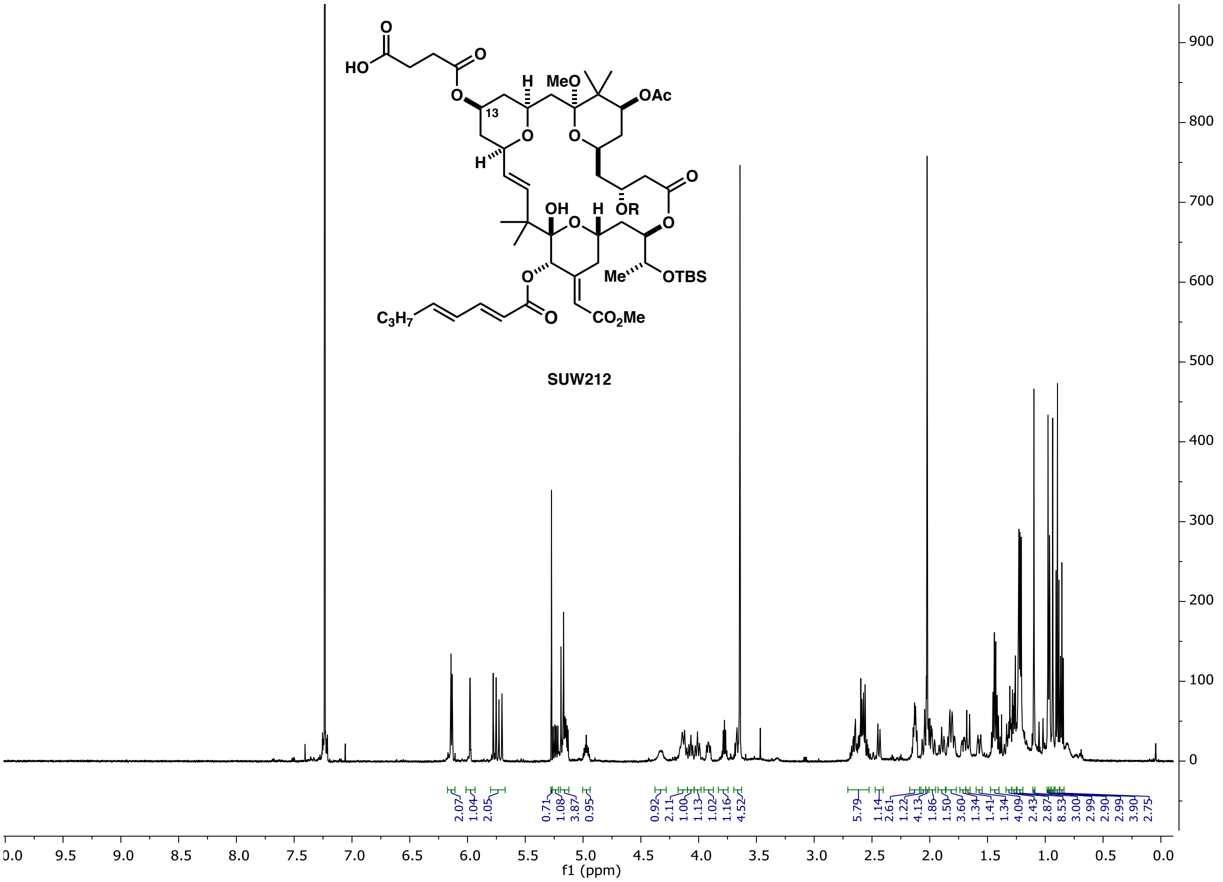

Supplemntary Figure 36. ^{1}H NMR (600 MHz, $CDCl_3$) of SUW212.

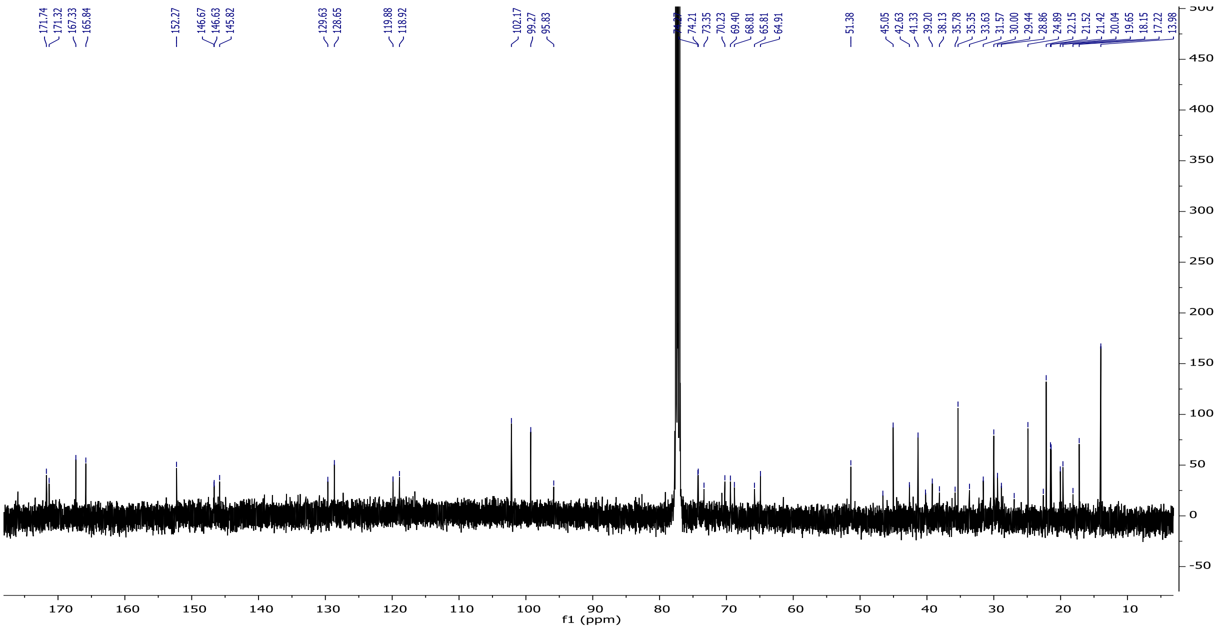

Supplementary Figure 37. ^{13}C NMR (126 MHz, $CDCl_3$) of SUW212.

PKC Binding Assay Protocol

The protein kinase C (PKC) affinity of bryostatin 1 and bryostatin analogs was performed via competition with ^{3}H-phorbol-12,13-dibutyrate (^{3}H-PDBu) as described below. This procedure entails a glass-fiber filtration method to determine bound radioligand.

Preparation of PKC binding assay buffer
To a 50 mL polypropylene tube was added Tris-HCl (pH 7.4, 1 M, 1 mL), KCl (1 M, 2 mL), $CaCl_2$ (0.1 M, 30 µL), and bovine serum albumin (BSA, 40 mg, Sigma-Aldrich). This mixture was diluted to 20 mL with deionized H_2O and mixed gently. The buffer was stored on ice until use. The final concentration of these constituents is shown in the following table:

Supplementary Table1. PKC binding assay buffer composition.

Constituent	Stock concentration	Quantity	Final Concentration
pH 7.4 Tris-HCl	1.0 M	1.0 mL	50 mM
KCl	1.0 M	2.0 mL	100 mM
$CaCl_2$	0.10 M	30 µL	0.15 mM
BSA	-	40 mg	2 mg/mL
Deionized H_2O	-	Final vol of 20 mL	-

Preparation of phosphatidylserine (PS) vesicles
For every two assays, 3.5 mg phosphatidylserine (Avanti Polar Lipids, porcine, 25 mg/mL $CHCl_3$ solution) was concentrated by removing chloroform under a stream of nitrogen followed by reduced pressure. The solid PS was suspended as vesicles in freshly prepared PKC binding assay buffer (3.5 mL) by sonicating six times for 30 sec, with a 30 sec rest between sonications (Branson Sonifier 250, power = 2, 50% duty cycle). The resulting milky cloudy mixture (1 mg/mL) was stored on ice until use.

Preparation of PKC isoform solution
Assay PKC was prepared by dissolving a 4 µg aliquot of the indicated recombinant human PKC isoform (Invitrogen) into 11.6 mL of PKC binding assay buffer (this amount is sufficient for two assays). The diluted PKC was stored on ice for immediate use.

Preparation of ^{3}H-PDBu solution
^{3}H-PDBu (American Radiolabeled Chemicals, Inc.; 1 mCi/mL acetone solution; specific activity: 20 µCi/mmol) was diluted 10-fold with DMSO. The resulting 500 nM stock solution was further diluted with DMSO to 30 nM.

Preparation of analog compound dilutions
Compound dilutions were prepared by serially diluting from a chosen "high" concentration by factors of 3 or 4. For each analog compound, seven concentrations were used to define the inhibition curve (i.e. for SUW200, the analog concentrations used were 3000 nM, 750 nM, 188 nM, 46.9 nM, 11.7 nM, 2.93 nM, and 0.73 nM).

"Master Mix" Solution

To a polypropylene tube was added 3.3 mL of 1 mg/mL PS vesicles solution, 11 mL of PKC isoform solution, and 1.1 mL of 30 nM ^{3}H-PDBu solution were added. The resulting solution was vortexed to mix and stored on ice.

PKC binding assay protocol

Materials:

- Glass-fiber filters (Whatman GF/B) were prepared by soaking them in a solution of aqueous polyethyleneimine (10% by vol, 18 mL) in deionized water (600 mL) for ≥1h.
- 500 mL "rinsing buffer" of 20 mM Tris, pH 7.4 was cooled on ice for the duration of the incubation period and for the remainder of the assay.

Triplicate data points were obtained for each analog concentration. For each data point, 280 µL of "Master Mix" Solution and 20 µL of analog compound at a specified concentration were added to a polypropylene tube. Non-specific ^{3}H-PDBu binding was assessed in triplicate by substitution of the analog compound with unlabeled PDBu (20 µL of a 75 µM stock, assay concentration: 5 µM). Maximal ^{3}H-PDBu binding was assessed in triplicate by substitution of the analog compound with 20 µL DMSO. The solutions were vortexed to mix, incubated at 37 °C for 10 min, and incubated on ice for at least 30 min prior to filtration. Using a Brandel Harvester, the assay contents from each polypropylene tube were vacuum-filtered through polyethylenimine-soaked filters, washing with rinsing buffer (3X) and drying first under vacuum for 5 min and then under ambient conditions for ≥ 2h. The resulting filters had circular perforations for each data point, which were removed with forceps and placed in a scintillation vial. Scintillation vials were filled with Bio-Safe II scintillation fluid (5 mL) and measured for radioactivity using a Beckman LS 6000SC scintillation counter. Counts per minute (cpm) were averaged for each triplicate dilution. The data were plotted – cpm vs. log(concentration) – using Prism® by GraphPad Software and an IC_{50} was determined using that program's built-in one-site competition least squares regression function. K_i values were calculated using the equation: $K_i = IC_{50} / (1 + ([^3\text{H-PDBu}] / K_d))$. The K_d of ^{3}H-PDBu for PKC isoforms was measured separately via saturation binding experiments under identical conditions.

PKCδ-GFP Translocation Assay Protocol

Cell Culture
Chinese hamster ovary factor K1 (CHO-k1, ATCC) cells were cultured in F-12 Kaighn's media (Hyclone, 10% fetal bovine serum, 1% penicillin/streptomycin added, reffered to as F-12 +/+ below) at 37 °C in an incubator (5% CO_2). Cell cultures were maintained by splitting cells 1:3 when they reached ~75-100% confluence (every 2-3 days) as follows:

Media was aspirated (taking care not to disturb adherent cells) and 3 mL of 0.25% trypsin EDTA (Gibco) was used to remove the cells from the culture flask (T75, Falcon). 1 mL of the cell suspension was then added to 9 mL of fresh F-12 +/+ and the sample was sub-cultured until reaching confluence (~2-3 days).

Cell Plating
A confluent culture of CHO-k1 cells was detached from a T75 flask with 3 mL 0.25% trypsin EDTA. Cells were counted using a Countess II Automated Cell Counter (Fisher). The cell suspension was diluted to 240,000 cells/mL with fresh F-12 +/+ and 2.5 mL of this diluted stock was added to one well in a 6-well plate. The cells were then cultured for 24 hours.

Transfection
Cells were transfected with Lipofectamine 2000 reagent (Invitrogen) or DA 13:11 at a 10:1 charge ratio as previously described by McKinlay *et al*. (*PNAS* **2017**, *114*, E448-E456).

Lipofectamine 2000
F-12 +/+ was aspirated and cells were washed with F-12 -/-. 2 mL of fresh F-12 -/- was then added to each well, taking care not to disturb adherent cells. For each well of CHO-k1 cells, 12.5 μL Lipofectamine 2000 reagent (Invitrogen) was added to 250 μL Opti-MEM reduced serum media (Invitrogen) in a polypropylene tube and incubated for 20 minutes at RT. Meanwhile, for each well, 4 μg of PKCδ-GFP pDNA and 250 μL Opti-MEM reduced serum media was added to a separate polypropylene tube. 250 μL of the Lipofectamine 2000 suspension was added to the DNA suspension and the solution incubated for 30 minutes at RT. 500 μL of the Lipofectamine/DNA suspension was added to the respective wells of the 6-well plate. The cells were then incubated at 37 °C (5% CO2) for ~24 hrs.

DA 13:11
F-12 +/+ was aspirated and cells were washed with F-12 -/-. 2.4 mL of fresh F-12 -/- was then added to each well, taking care not to disturb adherent cells. A 4 μg aliquot of PKCδ-GFP pDNA was added to PBS (pH 5.5, final volume 100 μL). 5.6 μL of DA 13:11 (2 mM stock in DMSO) was then added to the DNA solution, and the mixture was gently mixed (by flicking) for 20 seconds, at which point it was added directly to the respective wells of the 6-well plate. The cells were then incubated at 37 °C (5% CO2) for ~24 hrs.

Plating on Chambered Coverglass Slides

After incubation, the media was aspirated and cells were washed with PBS (2.0 mL) and trypsinized (500 μL). The cell suspension was then diluted with 2.0 mL F-12 +/+. 200 μL aliquots were added to 3 wells of a Lab-Tek II 4-well chambered coverglass slide (Fisher), producing 4 slides in total, each with three wells of cells. The cell suspension was directly diluted with 600 μL of additional F-12 +/+. The resulting samples were incubated for ~24 hrs. prior to imaging.

Dosing an acquiring data

Fluorescent images were obtained using a Leica SP8 White Light Confocal microscope and the Leica AF software package. Prior to analysis, media was aspirated and 800 μL of PBS (Hyclone, without Ca^{2+} or Mg^{2+}) supplemented with glucose (10 mM) was added to each well of the chambered coverglass slide. Bryostatin and bryostatin analogs were diluted to the appropriate concentration in 200 μL of 10 mM glucose in PBS. Cells were located for imaging and data was recorded for three wells in parallel, imaging at predetermined positions in each well using adaptive focus control. Cells were imaged at 30 second intervals following the addition of compound (set to t = 0) for 20-40 minutes. Data were recorded at room temperature. Images were exported as .lif files and fluorescence intensity was analyzed using FIJI (NIH) software. To monitor the translocation, small cytosolic regions of interest were selected in each cell, and fluorescence intensity values were plotted vs. time following background subtraction and normalization. Graphed data represents the average of at least replicates.

CD22 Surface Expression Protocol – NALM6 Cells

Cell culture

NALM6, clone G5 cells (ATCC) were cultured RPMI-1640 (Hyclone, + L-glutamine, + 10 mM HEPES, 10% fetal bovine serum added, 1% penicillin/streptomycin added, reffered to as RPMI-1640 unless otherwise noted below) at 37 °C in an incubator (5% CO_2). Cell cultures were maintained between 4 x 10^5 and 3 x 10^6 cells/mL according to vendor instructions (link below).

https://www.atcc.org/products/all/CRL-3273.aspx#culturemethod

Plating and dosing

Cell suspension from a confluent T75 flask (Fisher) was transferred to a 15 mL falcon tube and centrifuged at 1100 rpm for 7 minutes. The supernatant was aspirated, and the cell pellet was resuspended in ~5 mL of fresh RPMI-1640. Cells were counted using a Countess II Automated Cell Counter (Fisher). A 5.2 mL stock of 1 x 10^6 cells/mL was prepared by diluting an aliquot of the cell suspension with additional RPMI-1640. 199 μL of this stock was added to 24 wells (enough for triplicate measurements for 8 different experimental conditions) in a 96-well plate. Dosing was performed in triplicate for DMSO, bryostatin 1, and bryostatin analogs. DMSO (negative control), 10 nM bryostatin 1 (positive control), and untreated samples (negative control) were included in each experiment. 1 μL of DMSO or the appropriate stock solution of compound in DMSO was added to each well. Cells were incubated for 24 hours, at which point the cell suspensions were transferred to 1.5 mL Eppendorf tubes and diluted with 1.0 mL of PBS (Hyclone, without Ca^{2+} or Mg^{2+}). Samples were centrifuged at 2000 rpm for 5 min at RT. The supernatant was aspirated, and cell pellets were resuspended in 400-600 μL RPMI-1640. Cells were sub-cultured between 2 x 10^5 and 3 x 10^6 cells/mL in 24-well plates for an additional 24 hrs – 7 days, at which point CD22 surface expression was assayed by flow cytometry.

Flow cytometry

Cells from one well were counted using a Countess II Automated Cell Counter (Fisher). ~200,000 – 300,000 cells from each well were added to 1.5 mL Eppendorf tubes containing PBS (final volume ~1.2 mL). The cell suspensions were centrifuged at 1500 rpm for 7 minutes at 4 °C. The supernatant was aspirated, and the cell pellet was resuspended in 99 μL of pre-chilled FACS buffer (0.5% w/v BSA in PBS). 1 μL of PE Mouse Anti-Human CD22 (5 μL/1 x 10^6 cell test, BD Biosciences, Catalog No. 562859) was added and the solution was incubated at 4 °C for 30-45 minutes. Samples were then diluted with an additional 1.0 mL of FACS buffer and centrifuged at 1500 rpm for 7 minutes at 4 °C. The supernatant was aspirated, the cell pellets were resuspended in 200 μL FACS buffer, and the resulting suspensions were transferred to FACS tubes (Fisher, Catalog No. 352058). Cells were stained with DAPI and CD22 surface expression was analyzed using the FACScan Analyzer at the Stanford Shared FACS Facility. Data analysis was performed using FlowJo and Microsoft Excel.

CD22 Surface Expression Protocol – JB and 2F7 Cells

Culture Conditions. AIDS-NHL cell lines were incubated in IF10 medium, consisting of IMDM medium (Life technologies) containing 10% fetal bovine serum (FBS, Omega Scientific), 100 units/mL of penicillin, and 100 µg/ml of streptomycin (Invitrogen). Cells were incubated at 37 °C in 5% CO2.

AIDS-NHL cell lines activation Procedures. AIDS-NHL cells were cultured in a u-bottomed 96-well tissue culture plates with a cell density of 200,000 cells/well in a 200 µL volume of IF10 media containing the indicated equimolar concentration of bryostatin 1, SUW201 or SUW229. Cells were exposed to compound for the either 24 or 48 h before staining and flow cytometric analysis of receptor levels.

Flow Cytometry. Cells in each well were washed with phosphate buffered saline (PBS) containing 2% FBS, then cells were centrifuged for 9 min at 233 xg and resuspended in 50 µL of a 1:1 dilution of phosphate buffered saline (PBS): Human AB serum (Sigma). Cells were stained with anti-human CD22 (Biolegend, clone S-HCL-1, 363506) and were incubated at 4 ◦C for 25 minutes then cells were washed and fixed in 2% paraformaldehyde. Stained samples were stored at 4 °C. Flow cytometry samples were analyzed using a FACSCelesta (BD Biosciences) flow cytometer and data were analyzed using FlowJo software (version 10).

Biological data for individual bryostatin analogs

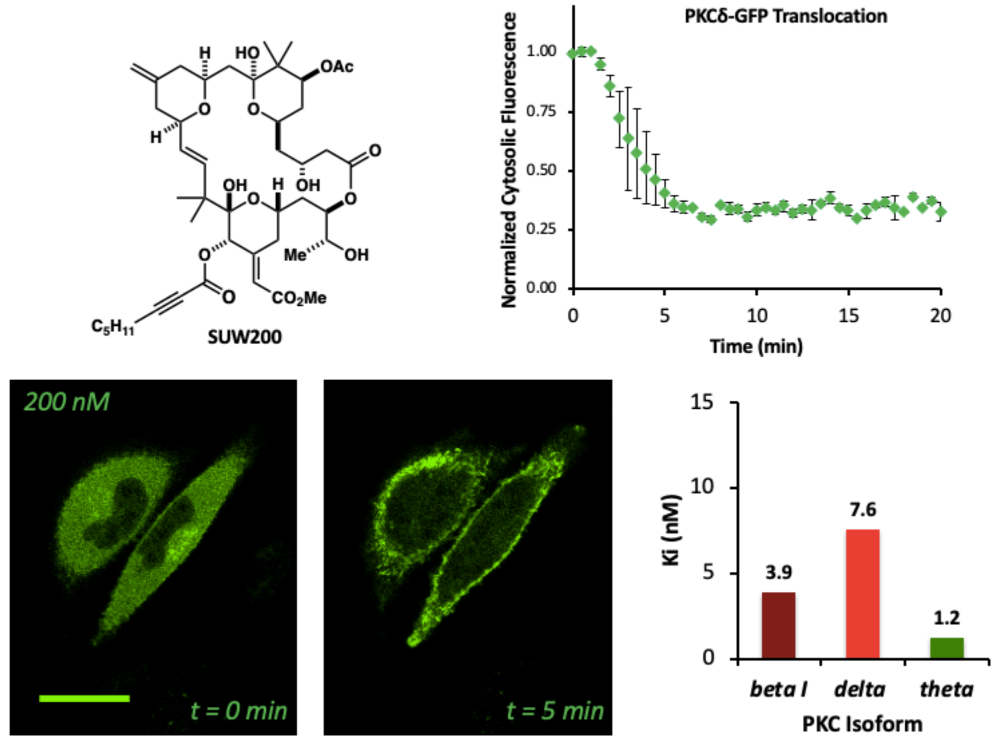

Supplementary Figure 38. Biological data for SUW200. Cytosolic fluorescence data presented as mean of n = 3 biological replicates with error bars = ±SE. Scale bar represents 25 µm.

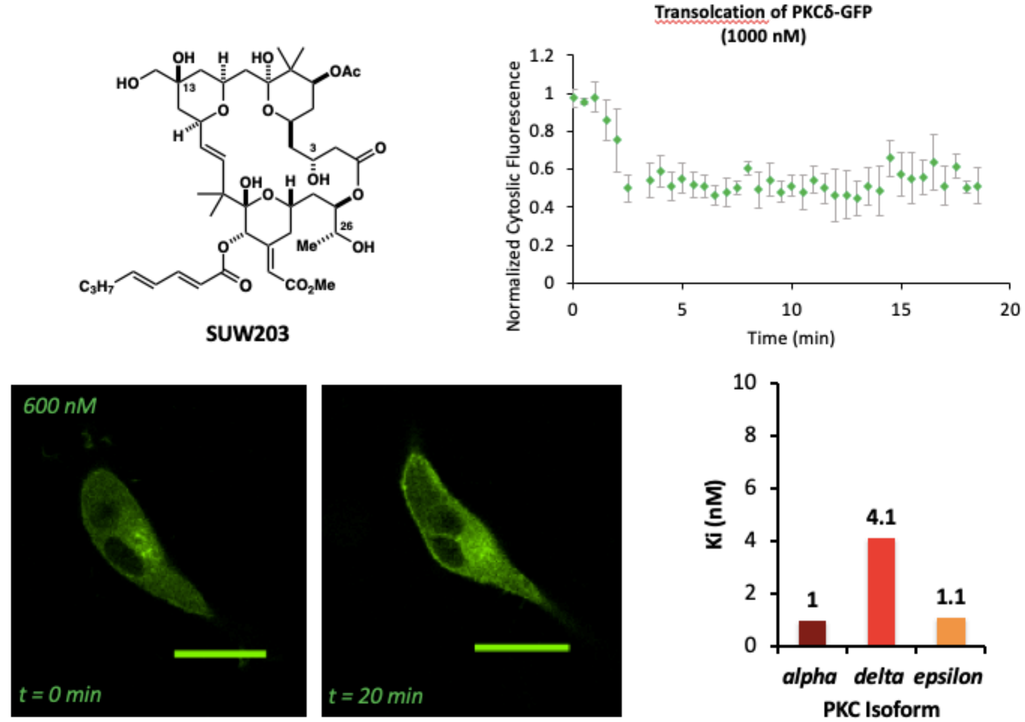

Supplementary Figure 39. Biological data for SUW203. Cytosolic fluorescence data presented as mean of n = 3 biological replicates with error bars = ±SE. Scale bar represents 25 µm.

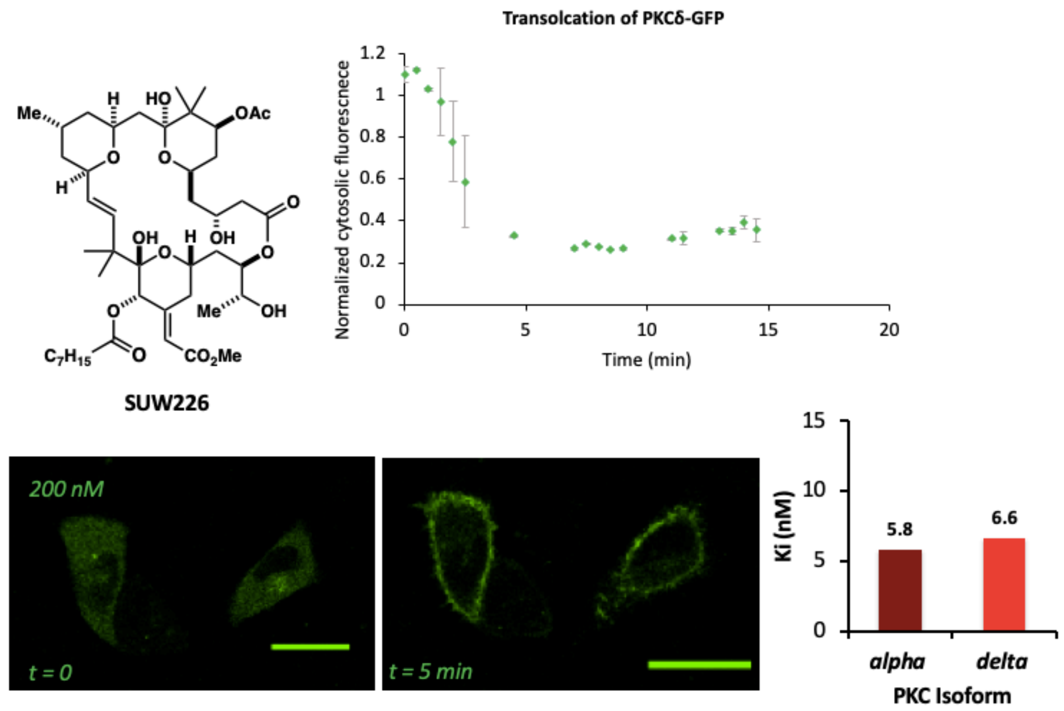

Supplementary Figure 40. Biological data for SUW226. Cytosolic fluorescence data presented as mean of n = 3 biological replicates with error bars = ±SE. Scale bar represents 25 µm.

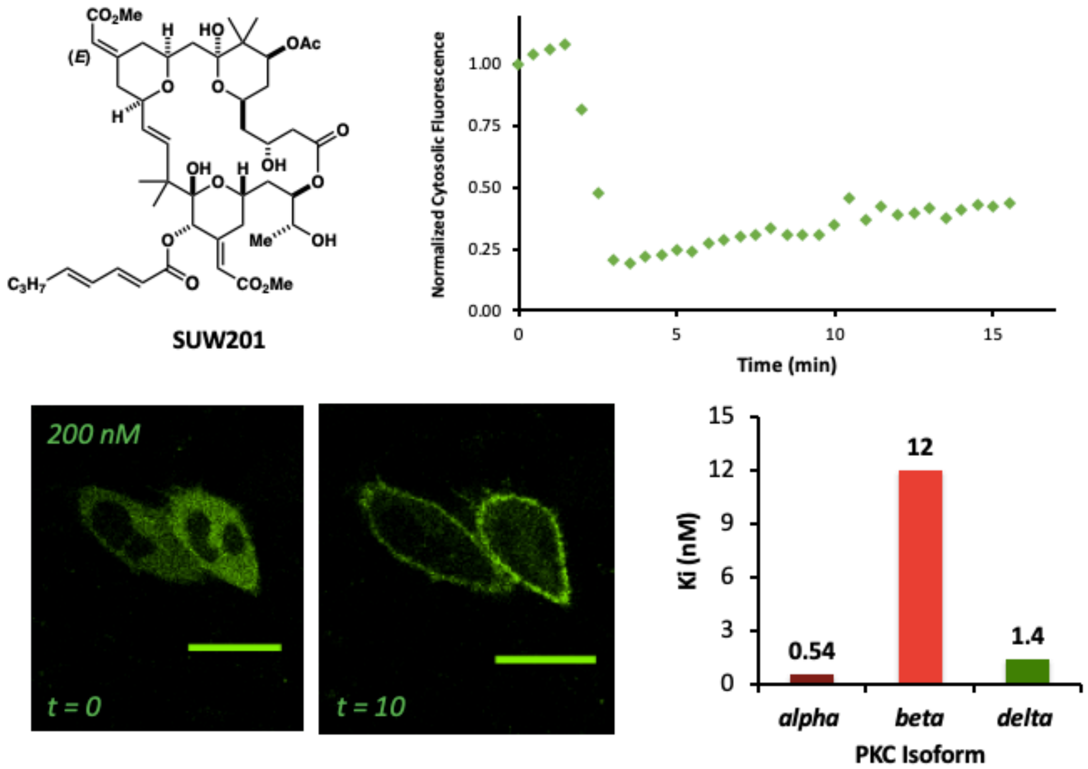

Supplementary Figure 41. Biological data for SUW201. Cytosolic fluorescence data presented as mean of n = 3 biological replicates with error bars = ±SE. Scale bar represents 25 µm.

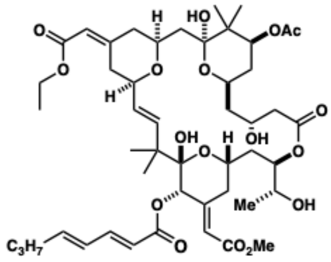

SUW217

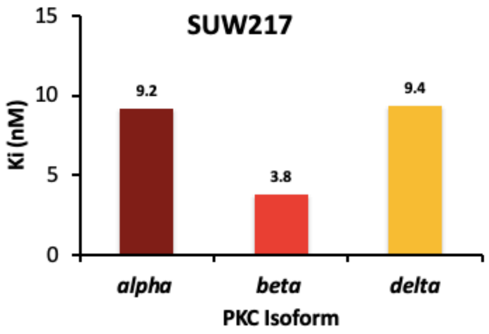

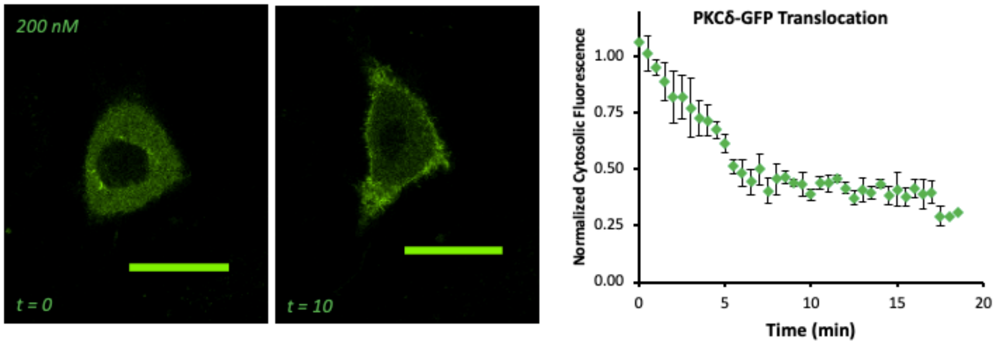

Supplementary Figure 42. Biological data for SUW217. Cytosolic fluorescence data presented as mean of n = 3 biological replicates with error bars = ±SE. Scale bar represents 25 µm.

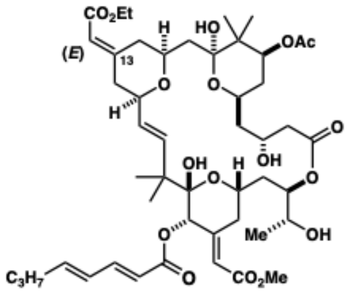

SUW218

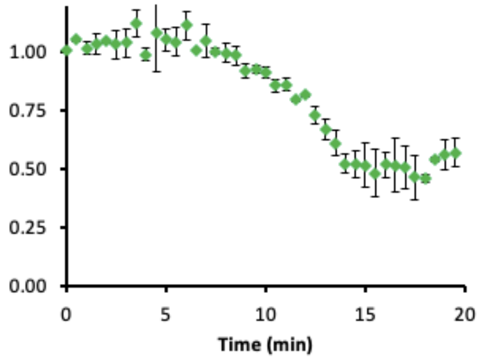

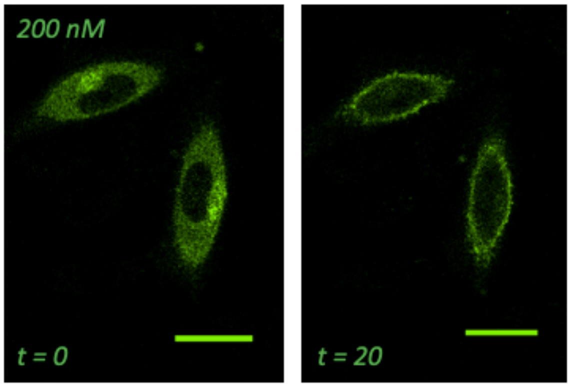

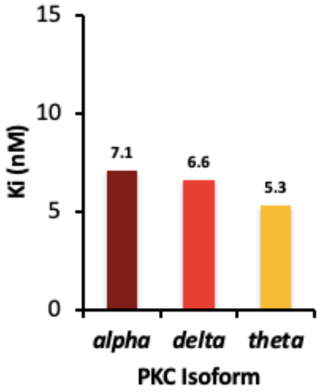

Supplementary Figure 43. Biological data for SUW218. Cytosolic fluorescence data presented as mean of n = 3 biological replicates with error bars = ±SE. Scale bar represents 25 µm.

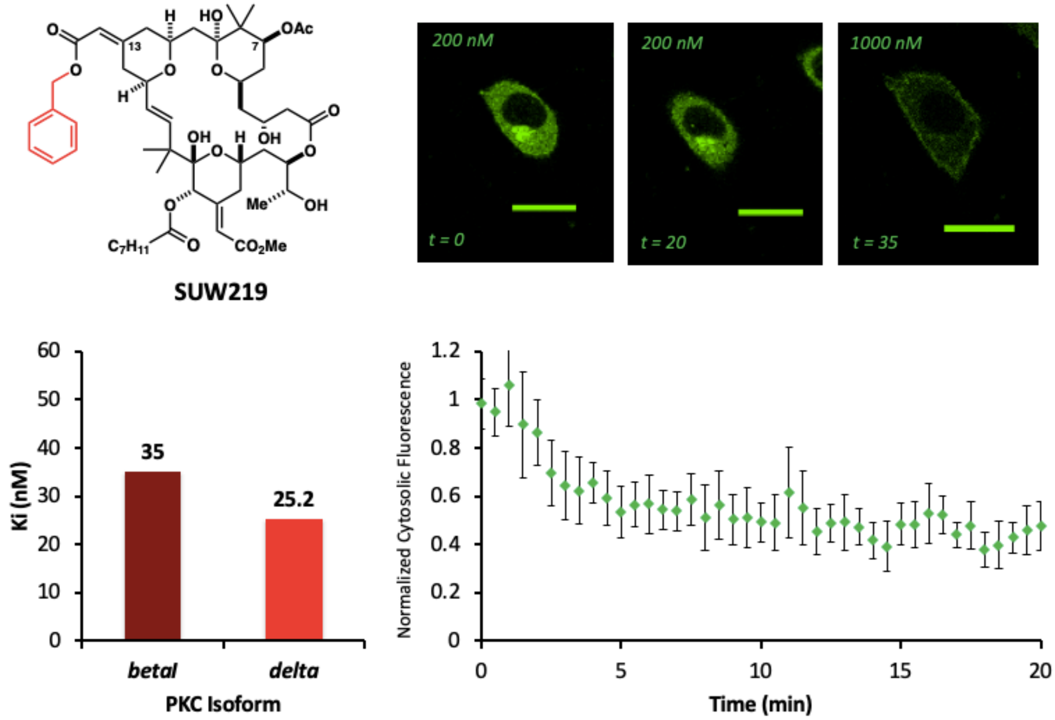

Supplementary Figure 44. Biological data for SUW219. Cytosolic fluorescence data presented as mean of n = 3 biological replicates with error bars = ±SE. Scale bar represents 20 µm.

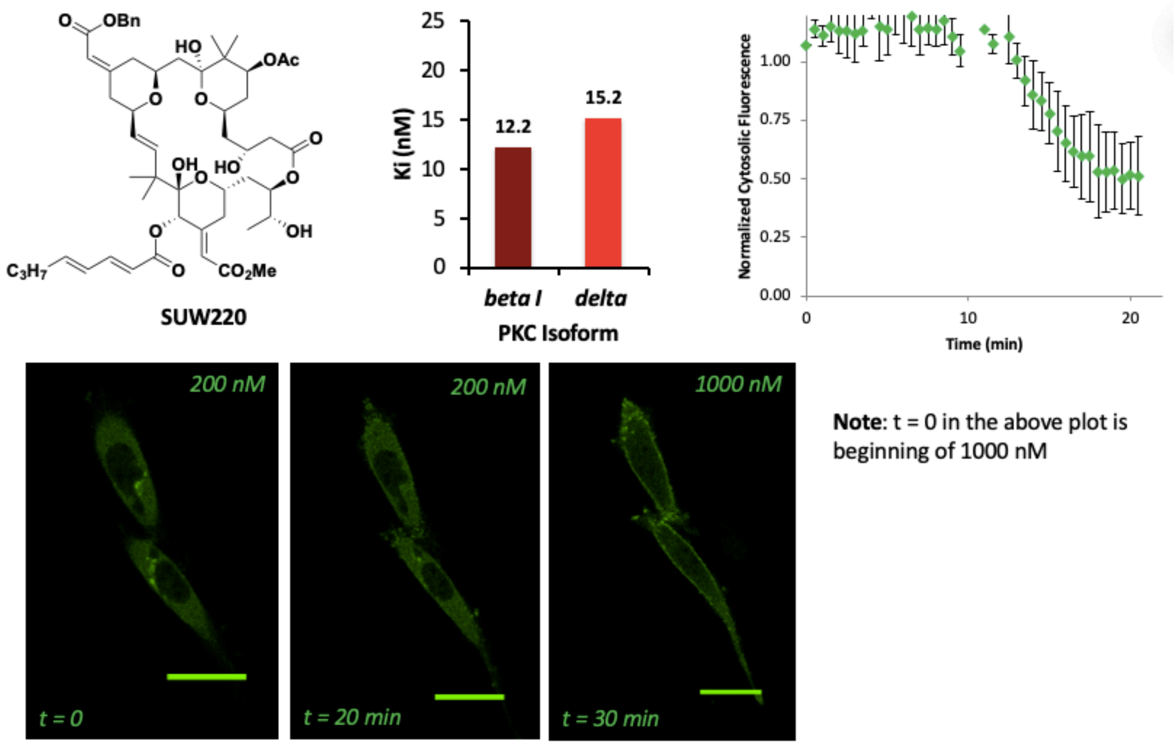

Supplementary Figure 45. Biological data for SUW220. Cytosolic fluorescence data presented as mean of n = 3 biological replicates with error bars = ±SE. Scale bar represents 25 µm.

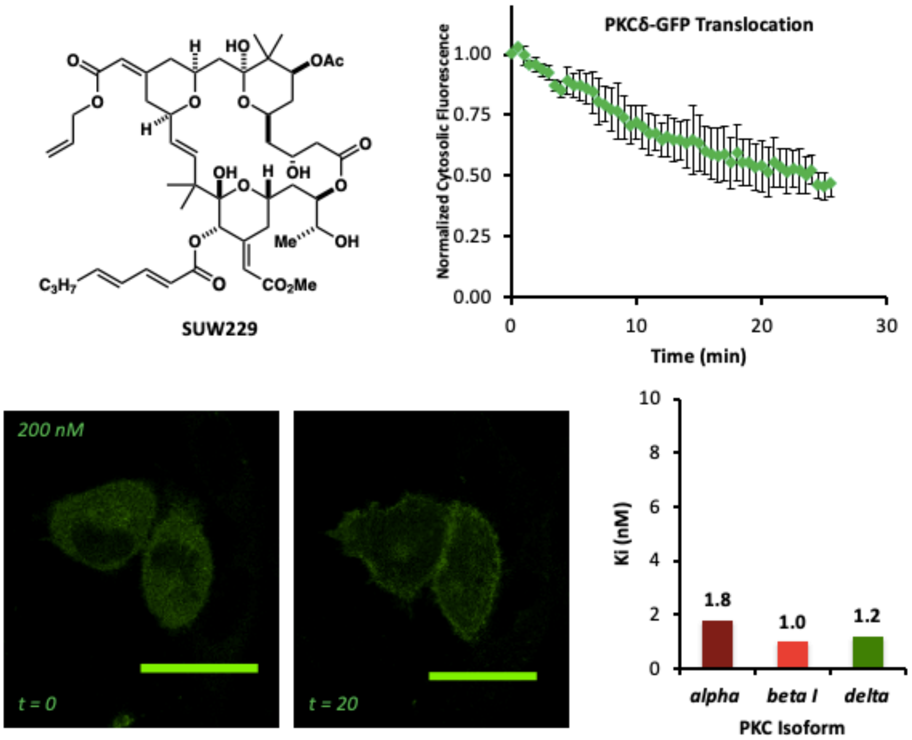

Supplementary Figure 46. Biological data for SUW229. Cytosolic fluorescence data presented as mean of n = 3 biological replicates with error bars = ±SE. Scale bar represents 25 µm.

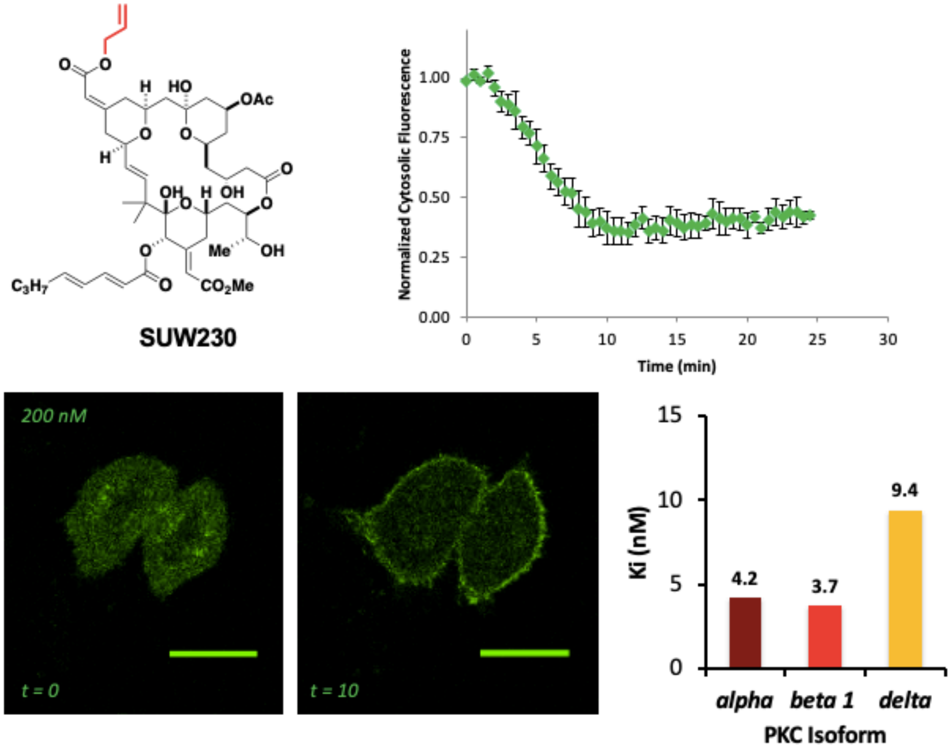

Supplementary Figure 47. Biological data for SUW230. Cytosolic fluorescence data presented as mean of n = 3 biological replicates with error bars = ±SE. Scale bar represents 20 µm.

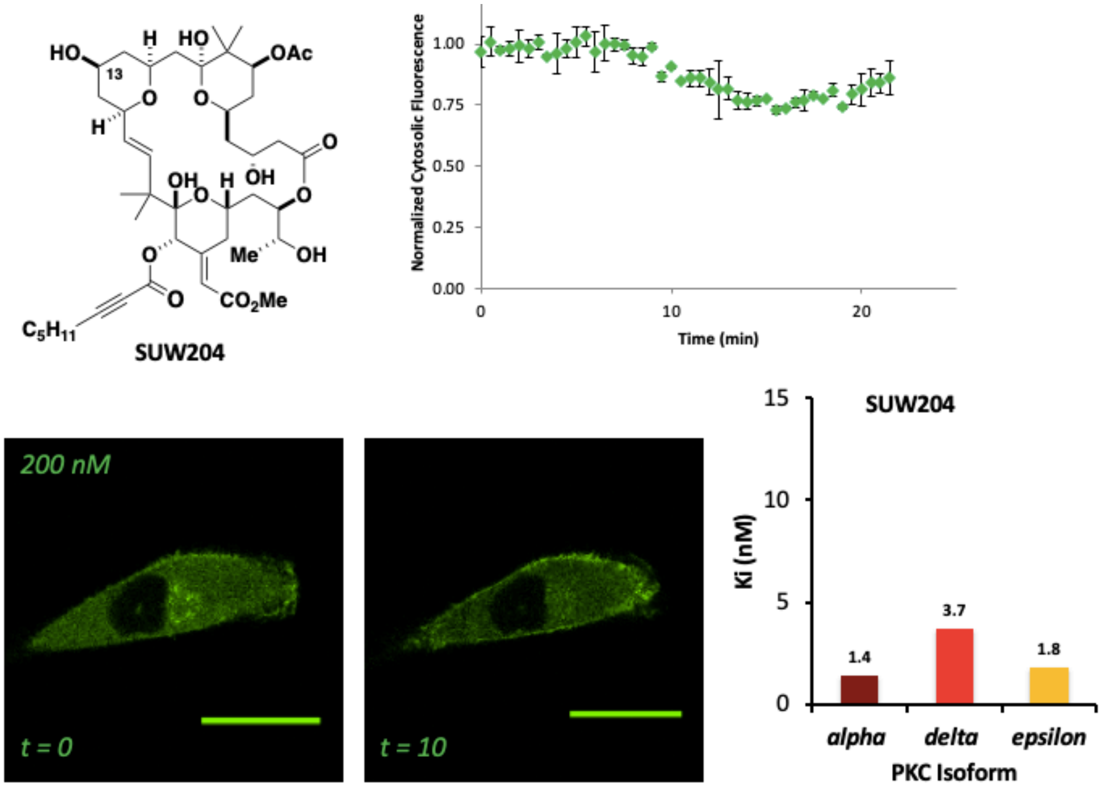

Supplementary Figure 48. Biological data for SUW204. Cytosolic fluorescence data presented as mean of n = 3 biological replicates with error bars = ±SE. Scale bar represents 25 μm.

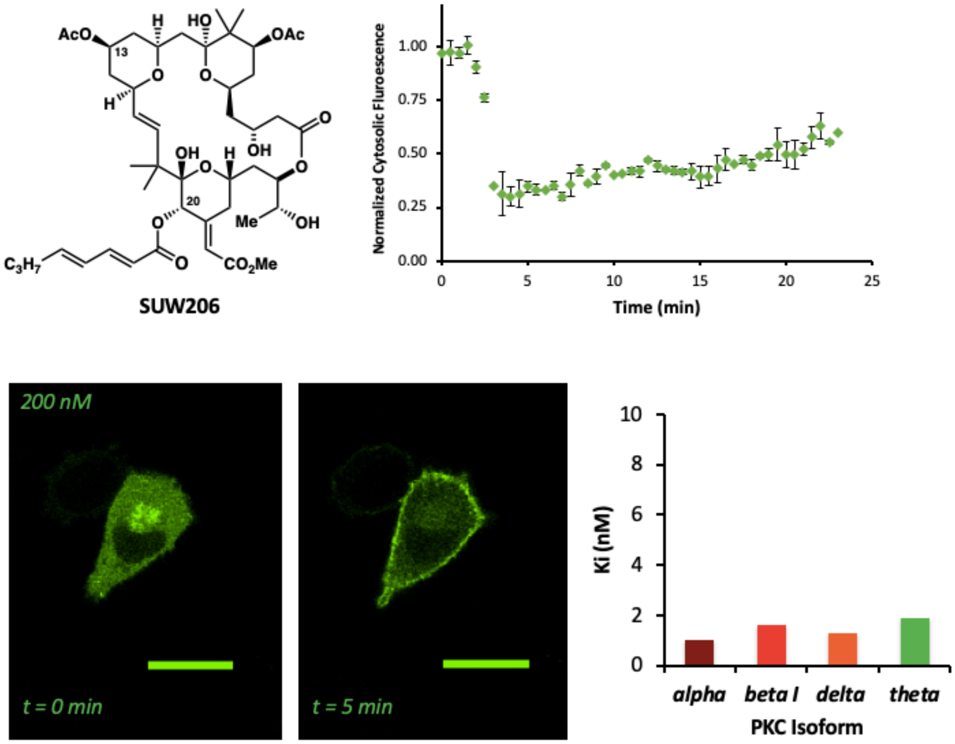

Supplementary Figure 49. Biological data for SUW206. Cytosolic fluorescence data presented as mean of n = 3 biological replicates with error bars = ±SE. Scale bar represents 20 μm.

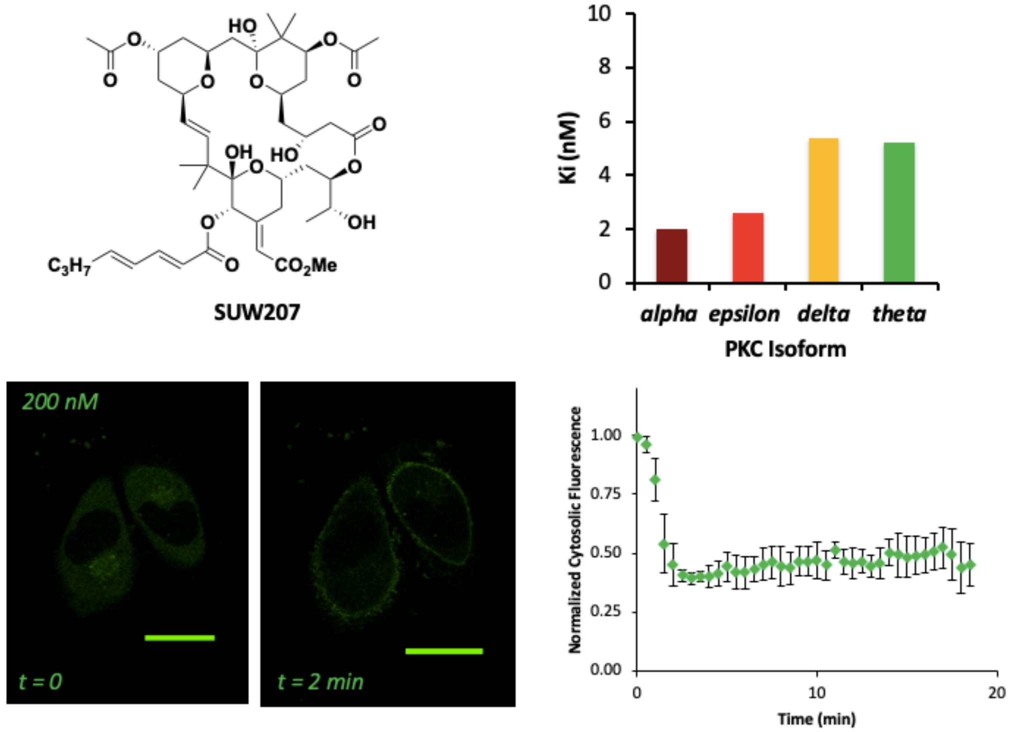

Supplementary Figure 50. Biological data for SUW207. Cytosolic fluorescence data presented as mean of n = 3 biological replicates with error bars = ±SE. Scale bar represents 25 μm.

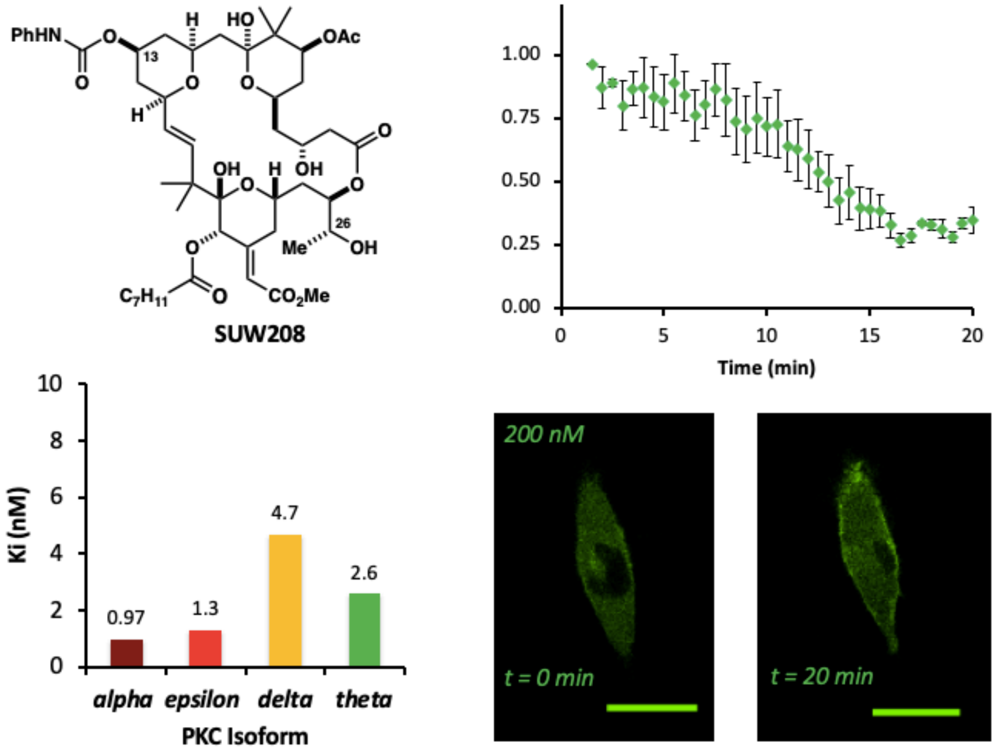

Supplementary Figure 51. Biological data for SUW208. Cytosolic fluorescence data presented as mean of n = 3 biological replicates with error bars = ±SE. Scale bar represents 25 μm.

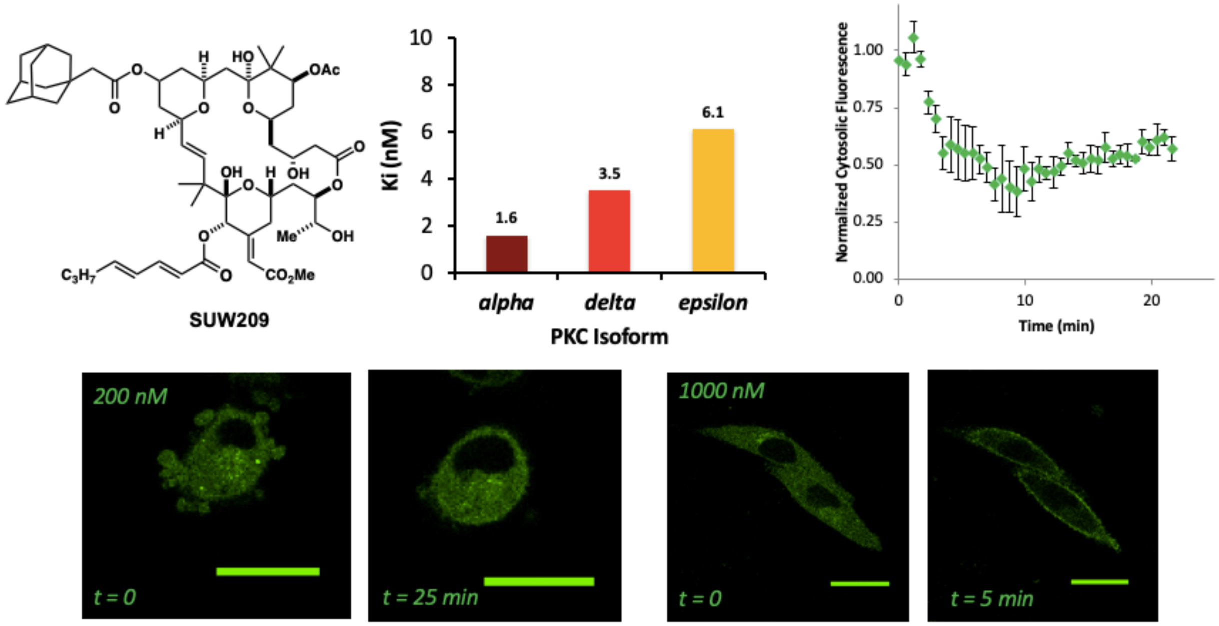

Supplementary Figure 52. Biological data for SUW209. Cytosolic fluorescence data presented as mean of n = 3 biological replicates with error bars = ±SE. Scale bar represents 20 µm.

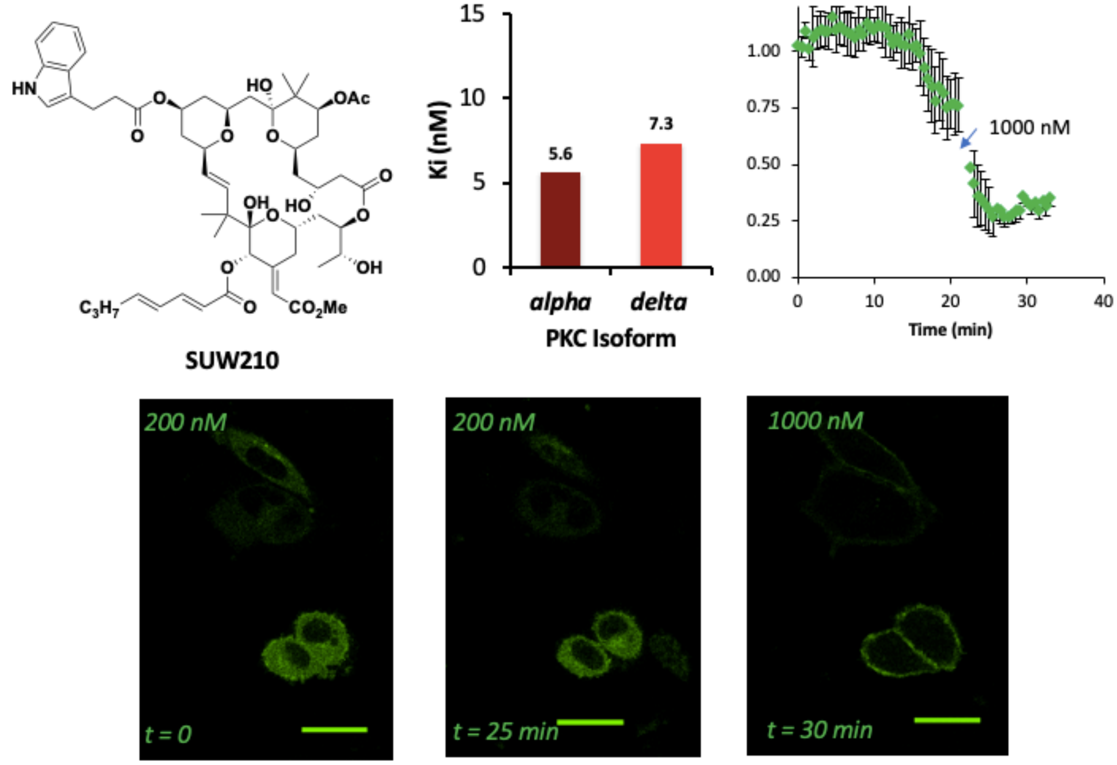

Supplementary Figure 53. Biological data for SUW210. Cytosolic fluorescence data presented as mean of n = 3 biological replicates with error bars = ±SE. Scale bar represents 25 µm.

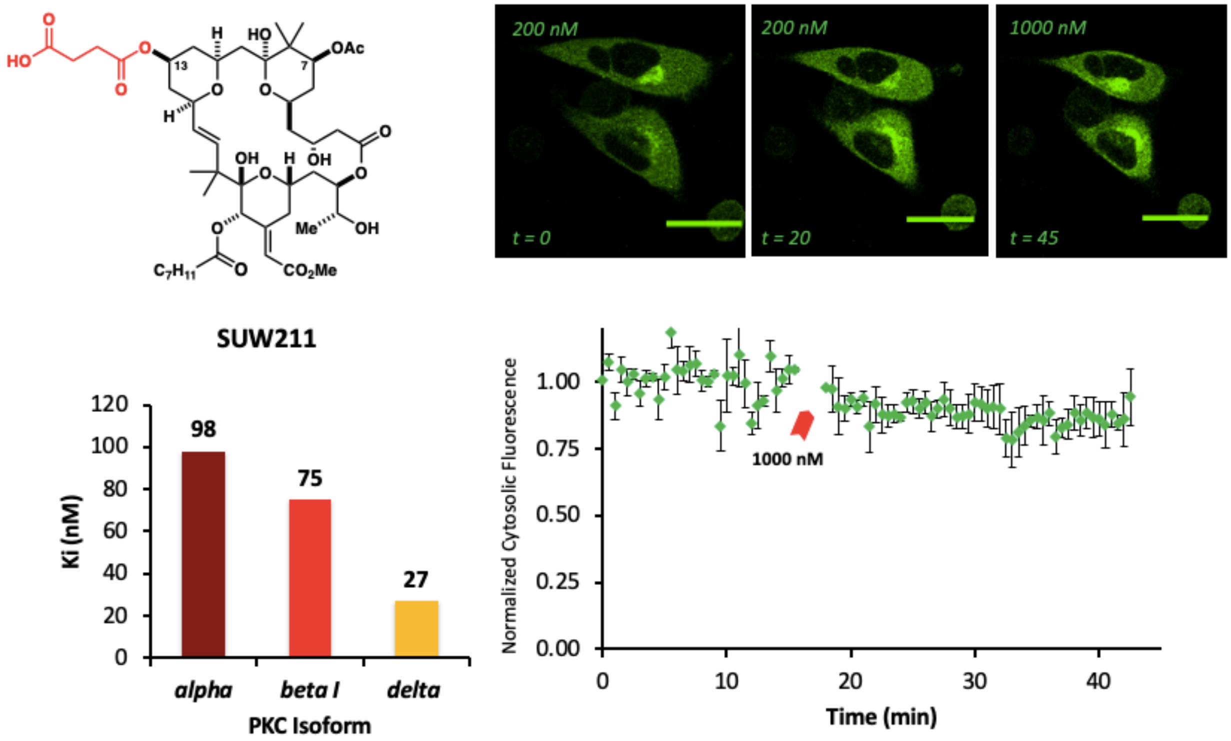

Supplementary Figure 54. Biological data for SUW211. Cytosoic fluorescence data presented as mean of n = 3 biological replicates with error bars = ±SE. Scale bar represents 25 µm.

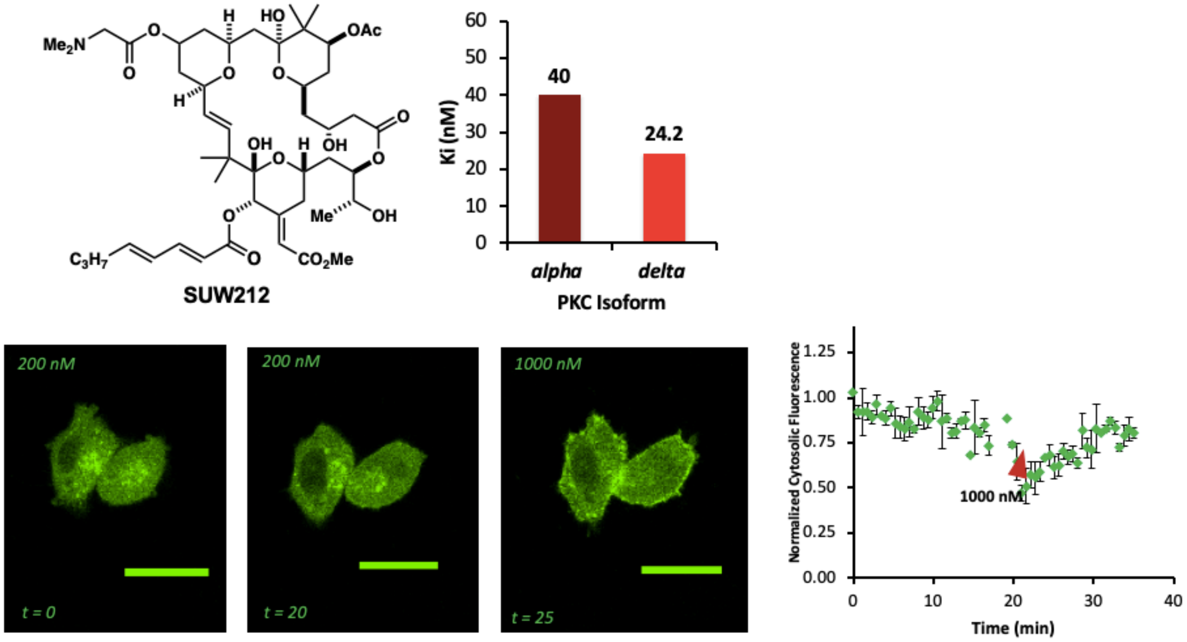

Supplementary Figure 55. Biological data for SUW212. Cytosolic fluorescence data presented as mean of n = 3 biological replicates with error bars = ±SE. Scale bar represents 20 µm.

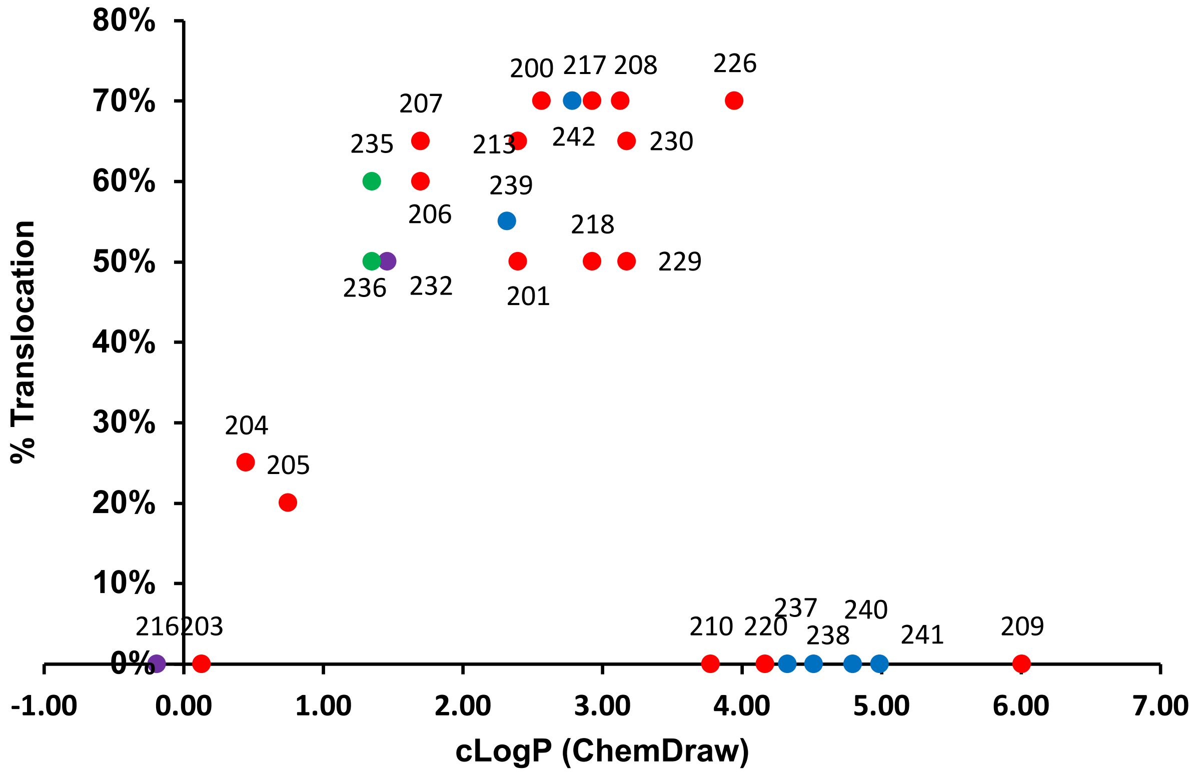

Supplementary Figure 56. Scatter plot of cLogP (calculated using ChemDraw) vs. % translocation at 200 nM. Red = C13-modified compound. Blue = C7-modified compound. Green = C20-modified compound. Purple = C13 and C7-modified compound. Note that not all compounds appear in the body of the paper.

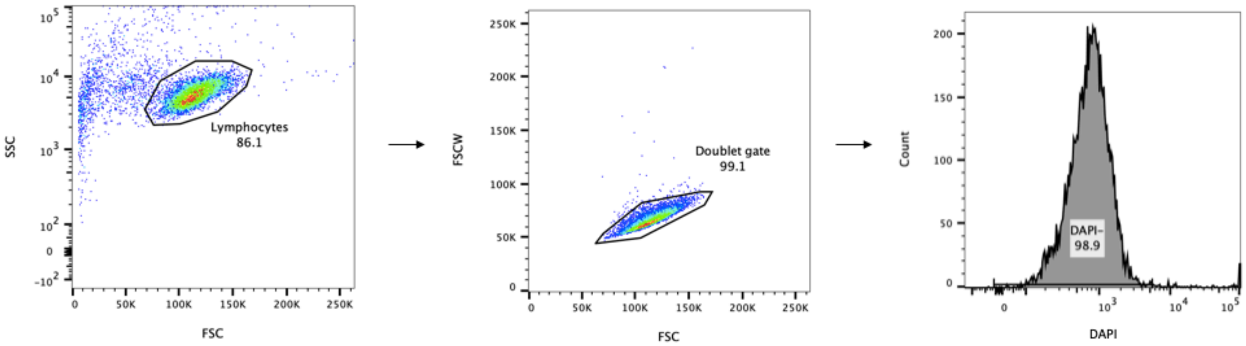

Supplementary Figure 57. Flow cytometry gating strategy. Gating strategy applies to flow data presented in Figure 6. FSC/SSC gates were set to eliminate debris. Doublet cells were eliminated by gating on FSCW/FSC to select for singlets. Dead cells were then eliminated by gating on DAPI negative cells. The geometric mean of fluorescence intensity for the fluorochrome on the anti-CD22 antibody was then recorded as a measurement of CD22 expression.
