# Supplementary material for: Synthesis and evaluation of designed PKC modulators for enhanced cancer immunotherapy
Source: Nat Commun. 2020 Apr 20;11:1879. doi: 10.1038/s41467-020-15742-7 (PMC7170889; doi:10.1038/s41467-020-15742-7)
Supplement: Supplementary file 2 — Reporting Summary [file 41467_2020_15742_MOESM2_ESM.pdf]

nature research

Corresponding author(s): Paul Wender

Last updated by author(s): Mar 6, 2020

Reporting Summary

Nature Research wishes to improve the reproducibility of the work that we publish. This form provides structure for consistency and transparency in reporting. For further information on Nature Research policies, see Authors & Referees and the Editorial Policy Checklist .

Statistics

For all statistical analyses, confirm that the following items are present in the figure legend, table legend, main text, or Methods section.

n/a	Confirmed	
☐	☒	The exact sample size (*n*) for each experimental group/condition, given as a discrete number and unit of measurement
☐	☒	A statement on whether measurements were taken from distinct samples or whether the same sample was measured repeatedly
☒	☐	The statistical test(s) used AND whether they are one- or two-sided<br>*Only common tests should be described solely by name; describe more complex techniques in the Methods section.*
☒	☐	A description of all covariates tested
☒	☐	A description of any assumptions or corrections, such as tests of normality and adjustment for multiple comparisons
☐	☒	A full description of the statistical parameters including central tendency (e.g. means) or other basic estimates (e.g. regression coefficient) AND variation (e.g. standard deviation) or associated estimates of uncertainty (e.g. confidence intervals)
☒	☐	For null hypothesis testing, the test statistic (e.g. *F*, *t*, *r*) with confidence intervals, effect sizes, degrees of freedom and *P* value noted<br>*Give P values as exact values whenever suitable.*
☒	☐	For Bayesian analysis, information on the choice of priors and Markov chain Monte Carlo settings
☒	☐	For hierarchical and complex designs, identification of the appropriate level for tests and full reporting of outcomes
☒	☐	Estimates of effect sizes (e.g. Cohen's *d*, Pearson's *r*), indicating how they were calculated

Our web collection on statistics for biologists contains articles on many of the points above.

Software and code

Policy information about availability of computer code

Data collection	Confocal data (Figure 5) was collected using a Leica SP8 White Light Confocal microscope and the Leica Application Suite X (version 3.5.19976.5).<br>Flow cytometry data was collected using the FACScan Analyzer at the Stanford Shared FACS Facility (Figure 6a, 6b) and a FACSCelesta (BD Biosciences) flow cytometer (Figure 6c, 6d).
Data analysis	PKC binding data was analyzed using GraphPad Prism (version 4.0a)<br>Confocal images were analyzed using the Fiji software (NIH, version Fiji for Mac OS X) and Microsoft Excel (version 16.34)<br>All flow cytometry data was analyzed using FlowJo (version 10), Microsoft Excel (version 16.34) and Prism (version 8.3.1).

For manuscripts utilizing custom algorithms or software that are central to the research but not yet described in published literature, software must be made available to editors/reviewers. We strongly encourage code deposition in a community repository (e.g. GitHub). See the Nature Research guidelines for submitting code & software for further information.

Data

Policy information about availability of data

All manuscripts must include a data availability statement. This statement should provide the following information, where applicable:

- Accession codes, unique identifiers, or web links for publicly available datasets
- A list of figures that have associated raw data
- A description of any restrictions on data availability

All data supporting the findings of this study is available within the Article and its Supplementary Information, or from the corresponding author upon request. Raw data associated with Table 1 and Figures 5 and 6 is also available upon request.

Field-specific reporting

Please select the one below that is the best fit for your research. If you are not sure, read the appropriate sections before making your selection.

☒ Life sciences ☐ Behavioural & social sciences ☐ Ecological, evolutionary & environmental sciences

For a reference copy of the document with all sections, see nature.com/documents/nr-reporting-summary-flat.pdf

Life sciences study design

All studies must disclose on these points even when the disclosure is negative.

Sample size	Sample sizes for confocal and flow cytometry experiments (n = 3) were chosen such that central tendencies (means) and variation (+/- SE) could be determined for each experimental condition and this information could be used to draw conclusions relative to appropriate controls.
Data exclusions	No data was excluded from analysis.
Replication	All confocal (Figure 5) and flow cytometry (Figure 6) experiments were performed in n = 3 biological replicates. All attempts at replication were successful.
Randomization	Samples of commercial and non-commercial cell lines were randomly segregated in to experimental groups (n = 3 biological replicates) for both confocal and flow cytometry studies.
Blinding	Blinding was not relevant to our study as all samples analyzed were homogeneous cell lines which were randomly segregated in to experimental groups.

Reporting for specific materials, systems and methods

We require information from authors about some types of materials, experimental systems and methods used in many studies. Here, indicate whether each material, system or method listed is relevant to your study. If you are not sure if a list item applies to your research, read the appropriate section before selecting a response.

Materials & experimental systems

n/a	Involved in the study	
☐	☒	Antibodies
☐	☒	Eukaryotic cell lines
☒	☐	Palaeontology
☒	☐	Animals and other organisms
☒	☐	Human research participants
☒	☐	Clinical data

Methods

n/a	Involved in the study	
☒	☐	ChIP-seq
☐	☒	Flow cytometry
☒	☐	MRI-based neuroimaging

Antibodies

Antibodies used	PE Mouse Anti-Human CD22 (Supplier: BD Biosciences, Catalog No. 562859, Clone name: HIB22, 25 µg/mL) used in Figure 6a, 6b<br>Anti-human CD22 (Supplier: Biolegend, Catalog number: 363506, Clone name: S-HCL-1, 25 µg/mL) used in Figure 6c, 6d
Validation	PE Mouse Anti-Human CD22 (Supplier: BD Biosciences, Catalog No. 562859, Clone name: HIB22):<br>The manufacturer's website (https://www.bdbiosciences.com/us/applications/research/b-cell-research/surface-markers/human/pe-mouse-anti-human-cd22-hib22/p/562859) provides histograms comparing the staining of whole blood with PE Mouse Anti-Human CD22 and PE Mouse IgG1, κ Isotype Control demonstrating specific affinity for human CD22.<br><br>Anti-human CD22 (Supplier: Biolegend, Catalog number: 363506, Clone name: S-HCL-1)<br>The manufacturers website states, "Each lot of this antibody is quality control tested by immunofluorescent staining with flow cytometric analysis."

Eukaryotic cell lines

Policy information about cell lines

Cell line source(s)	CHO-k1, NALM6, and 2F7 cells were obtained from ATCC.<br>The JB cell line was obtained from Ashlee Moses at Oregon Health Sciences University.

Authentication	Cell lines were obtained from ATCC (CHO-k1, NALM6, 2F7) or directly from the original investigator who isolated them (JB), but were not subjected to additional authentication.
Mycoplasma contamination	CHO-k1 and NALM6 cells were not tested for mycoplasma contamination.<br>JB and 2F7 cells tested negative for mycoplasma contamination.
Commonly misidentified lines<br>(See ICLAC register)	No commonly misidentified cell lines were used in this study.

Flow Cytometry

Plots

Confirm that:

- [x] The axis labels state the marker and fluorochrome used (e.g. CD4-FITC).
- [x] The axis scales are clearly visible. Include numbers along axes only for bottom left plot of group (a 'group' is an analysis of identical markers).
- [x] All plots are contour plots with outliers or pseudocolor plots.
- [x] A numerical value for number of cells or percentage (with statistics) is provided.

Methodology

Sample preparation	NALM6 cells: a detailed protocol describing the sample preparation for NALM6 cells can be found on page SI-56 of the Supplementary Information.<br><br>JB and 2F7 cells: a detailed protocol describing the sample preparation for JB and 2F7 cells can be found on page SI-57 of the Supplementary Information.
Instrument	CD22 expression in NALM6 cells was analyzed using the FACScan Analyzer at the Stanford Shared FACS Facility (https://facs.stanford.edu/instruments/scanford)<br>CD22 expression in JB and 2F7 cells was analyzed using a FACSCelesta flow cytometer (BD Biosciences, model number 660344).
Software	FlowJo (version 10) was used to analyze flow cytometry data.
Cell population abundance	Cells were not sorted as part of this study. Homogeneous populations of the indicated cell lines were analyzed for CD22 expression as outlined in the Supplementary Information. 10,000 cells/event were recorded.
Gating strategy	FSC/SSC gates were set to eliminate debris. Doublet cells were eliminated by gating on FSCW/FSC to select for singlets. Dead cells were then eliminated by gating on DAPI negative cells. The geometric mean of fluorescence intensity for the fluorochrome on the anti-CD22 antibody was then recorded as a measurement of CD22 expression.

- [x] Tick this box to confirm that a figure exemplifying the gating strategy is provided in the Supplementary Information.
